# Photoperiod and Vernalization Control of Flowering-Related Genes: A Case Study of the Narrow-Leafed Lupin (*Lupinus angustifolius* L.)

**DOI:** 10.3389/fpls.2020.572135

**Published:** 2020-10-26

**Authors:** Sandra Rychel-Bielska, Piotr Plewiński, Bartosz Kozak, Renata Galek, Michał Ksia̧żkiewicz

**Affiliations:** ^1^Department of Genetics, Plant Breeding and Seed Production, Wrocław University of Environmental and Life Sciences, Wrocław, Poland; ^2^Department of Genomics, Institute of Plant Genetics, Polish Academy of Sciences, Poznań, Poland

**Keywords:** vernalization, photoperiod, flowering, expression, *FLOWERING LOCUS T*, duplication, deletion

## Abstract

Narrow-leafed lupin (*Lupinus angustifolius* L.) is a moderate-yielding legume crop known for its high grain protein content and contribution to soil improvement. It is cultivated under photoperiods ranging from 9 to 17 h, as a spring-sown (in colder locations) or as an autumn-sown crop (in warmer regions). Wild populations require a prolonged cold period, called vernalization, to induce flowering. The key achievement of *L. angustifolius* domestication was the discovery of two natural mutations (named *Ku* and *Jul*) conferring vernalization independence. These mutations are overlapping deletion variants in the promoter of *LanFTc1*, a homolog of the *Arabidopsis thaliana FLOWERING LOCUS T* (*FT*) gene. The third deletion, named here as *Pal*, was recently found in primitive germplasm. In this study, we genotyped *L. angustifolius* germplasm that differs in domestication status and geographical origin for *LanFTc1* alleles, which we then phenotyped to establish flowering time and vernalization responsiveness. The *Ku* and *Jul* lines were vernalization-independent and early flowering, wild (*ku*) lines were vernalization-dependent and late flowering, whereas *the Pal* line conferred intermediate phenotype. Three lines representing *ku*, *Pal*, and *Ku* alleles were subjected to gene expression surveys under 8- and 16-h photoperiods. *FT* homologs (*LanFTa1*, *LanFTa2*, *LanFTc1*, and *LanFTc2*) and some genes selected by recent expression quantitative trait loci mapping were analyzed. Expression profiles of *LanFTc1* and *LanAGL8* (*AGAMOUS-like 8*) matched observed differences in flowering time between genotypes, highlighted by high induction after vernalization in the *ku* line. Moreover, these genes revealed altered circadian clock control in *Pal* line under short days. *LanFD* (*FD*) and *LanCRLK1* (*CALCIUM/CALMODULIN-REGULATED RECEPTOR-LIKE KINASE 1*) were negatively responsive to vernalization in *Ku* and *Pal* lines but positively responsive or variable in *ku*, whereas *LanUGT85A2* (*UDP-GLUCOSYL TRANSFERASE 85A2*) was significantly suppressed by vernalization in all lines. Such a pattern suggests the opposite regulation of these gene pairs in the vernalization pathway. *LanCRLK1* and *LanUGT85A2* are homologs of *A. thaliana* genes involved in the *FLOWERING LOCUS C* (*FLC*) vernalization pathway. Lupins, like many other legumes, do not have any *FLC* homologs. Therefore, candidate genes surveyed in this study, namely *LanFTc1*, *LanAGL8, LanCRLK1*, and *LanUGT85A2*, may constitute anchors for further elucidation of molecular components contributing to vernalization response in legumes.

## Introduction

Narrow-leafed lupin (*Lupinus angustifolius* L.) is a legume plant that is cultivated as “green” manure or as a grain crop for animal feed or human consumption (Kurlovich, 2002). The exploitation of *L. angustifolius* as a crop is advantageous in many respects. First, *L. angustifolius* cultivation has a positive influence on soil fertility due to the mobilization of soil-bound phosphorus and diazotrophic nitrogen fixation (Evans et al., 1987; Lambers et al., 2013). Moreover, *L. angustifolius* grains are characterized by high protein content and the use of them in livestock farming systems has many benefits in terms of the economic and environmental impact (Abraham et al., 2019). As seed alkaloid content was reduced by breeding below 0.01% of seed dry weight, a bitter taste, a typical feature of lupins, was fully eliminated from modern cultivars (Kamel et al., 2016). Additionally, the postharvest stubble can be now safety grazed by livestock, because the risk of lupinosis disease was diminished by the introduction of Phomospis stem blight resistance genes (Mulholland et al., 1976; Jago et al., 1982; Cowling et al., 1987; Williamson et al., 1994; Shankar et al., 2002; Ksia̧żkiewicz et al., 2020). Furthermore, *L. angustifolius* is currently being promoted in human food markets as a result of its nutritional, metabolomic, and other health benefits (Foyer et al., 2016; Kouris-Blazos and Belski, 2016).

Cultivated *L. angustifolius* germplasm primarily originated from the western Mediterranean basin (Mousavi-Derazmahalleh et al., 2018b). Natural adaptation of wild *L. angustifolius* populations to the Mediterranean climate is the requirement of the prolonged cold period (i.e., vernalization) during germination or juvenile phase to induce flowering (Gladstones and Hill, 1969; Rahman and Gladstones, 1972; Landers, 1995; Adhikari et al., 2012). The selection of early phenology was based on the removal of the vernalization requirement, a major achievement in the domestication of *L. angustifolius*, enabling temperature-independent sowing (French and Buirchell, 2005). *L. angustifolius* is grown in various environments ranging from the subtropics (Australia) and Mediterranean regions (including Morocco, Spain, southern France, and Italy), through temperate oceanic climates (such as the United Kingdom, northern France, and Benelux), humid continental climates (for example in Germany, the Baltic countries, and Ukraine), though to the subarctic zone, localized as far north as 60° latitude (Russia). In warmer regions it is usually autumn-sown, whereas in colder regions it is exclusively spring-sown (Annicchiarico and Carroni, 2009).

Lupin crops are cultivated in various photoperiod conditions, ranging from about 9 h (autumn sowing in Australia) to 16–17 h (spring sowing in Baltic countries and Russia) (Ksia̧żkiewicz et al., 2017). The vernalization requirements of wild populations are so demanding that they can only be completely fulfilled by spring sowing in northern locations (Adhikari et al., 2012). Worldwide, *L. angustifolius* cultivation is based on vernalization independence selected from only two natural mutations (named *Ku* and *Jul*) that were discovered in domesticated germplasm just a little over half a century ago (Mikołajczyk, 1966; Gladstones and Hill, 1969). The use of only two donors of early flowering in *L. angustifolius* breeding, followed by a strong selection of key agronomic traits, have resulted in a lack of phenological diversity in domesticated germplasm (Stefanova and Buirchell, 2010; Berger et al., 2012; Cowling, 2020). Therefore, further adaptation of this crop to new agronomic conditions resulting from a rapidly changing climate, may only be possible with the incorporation of novel genetic resources of intermediate phenology.

During recent years *L. angustifolius* was supplemented with numerous molecular resources, including bacterial artificial chromosome libraries carrying nuclear DNA inserts, consecutively improved linkage maps with sequence-defined markers, transcriptome assemblies, and a progressively updated genome sequence of the reference cultivar Tanjil (Kasprzak et al., 2006; Nelson et al., 2006; Gao et al., 2011; Yang et al., 2013; Kamphuis et al., 2015; Wyrwa et al., 2016; Hane et al., 2017; Zhou et al., 2018; Kozak et al., 2019). These resources were harnessed to reveal the genetic identity of *Ku*, which was found to be a homolog of a *FLOWERING LOCUS T* (*FT*) gene, named *LanFTc1* (Ksia̧żkiewicz et al., 2016; Nelson et al., 2017). *FT* gene is a well-recognized floral integrator gene, promoting flowering in response to environmental conditions signaled by photoperiod, vernalization, and circadian clock pathways (Turck et al., 2008). The functional mutation underlying the domesticated *Ku* allele in *L. angustifolius* was assigned to the 1423 bp deletion in the promoter region of *LanFTc1*, carrying potential binding sites for several transcription factors acting as *FT* gene repressors in *Arabidopsis thaliana* (Nelson et al., 2017). Interestingly, the second *L. angustifolius* early phenology mutation, *Jul*, was recently revealed to be the third *LanFTc1* allele, in the form of a 5162 bp deletion in the promoter region, fully encompassing that 1423 bp *Ku* deletion (Taylor et al., 2019). Screening of germplasm resources with the *LanFTc1* markers resulted in the identification of a fourth *LanFTc1* allele in wild population line originating from Palestine (a country annotated as Israel in Australian collection or Egypt in Polish gene bank), carrying 1208 bp deletion partially overlapping with domesticated *Ku* deletion (Rychel, 2018; Taylor et al., 2019). This Palestinian allele was named here as *Pal*.

Some genes involved in flowering time regulation are under strict circadian clock control and their expression levels fluctuate during a day (Shim et al., 2017). One such example in *Arabidopsis* is the *FT* gene, which expression is correlated with the stability of CONSTANS (CO) protein and shows two peaks, the first in the morning (lower) and the second in the evening (higher) (Turck et al., 2008). This CO-dependent regulation is facilitated by enhancer protein binding to specific sites in the promoter sequence, located approximately 5 kbp upstream of the first codon, which results in forming a DNA loop bringing all the components together (Cao et al., 2014). Interestingly, *L. angustifolius* allelic variants of the *LanFTc1* promoter have the distal binding sequences (CCAAT-boxes) preserved (Ksia̧żkiewicz et al., 2016; Nelson et al., 2017; Taylor et al., 2019). This study sought to explore whether the altered length of the *LanFTc1* promoter is associated with the altered circadian clock control of *LanFTc1* and other related genes.

Taking into consideration the (i) role of *FT* genes in integrating key environmentally responsive pathways, (ii) demonstrated allelic variability of *LanFTc1* gene associated with flowering time, and (iii) wide range of environmental variables occurring at lupin cultivation sites, we decided to explore the photoperiod and vernalization responsiveness in *L. angustifolius.* Here, the *L. angustifolius* germplasm differing in domestication status and geographical origin was genotyped for *LanFTc1* alleles and phenotyped for flowering time and vernalization responsiveness. Then, the transcriptomic response of candidate genes from the vernalization pathway in early (*ku*), intermediate (*Pal*), and late flowering (*Ku*) *L. angustifolius* germplasm was assayed under contrasting photoperiod and vernalization conditions, accounting the influence of circadian rhythm control. This study discusses the hypothetical function of these genes in terms of flowering time regulation and response to vernalization.

## Materials and Methods

### Plant Material

Ninety-two *L. angustifolius* lines were subjected to genotyping and phenotyping. Lines were derived from the European Lupin Gene Resources Database maintained by the Poznań Plant Breeding Ltd. station located in Wiatrowo. The lines originated from 13 countries, including Spain (32 lines), Poland (15), Australia (11), Russia (11), Germany (7), Italy (4), Belarus (3), Israel (2), Algeria, Morocco, Palestine, Portugal and Republic of South Africa (1). Accession numbers and information on the domestication status and country of origin are provided in Supplementary Table 1.

### Identification of *LanFTc1* Alleles

Young leaf tissue from three biological replicates per plant was sampled. Frozen (−80°C) plant tissue (50 mg) was homogenized using TissueLyser II (Qiagen, Hilden, Germany) and two stainless steel beads (ø 5 mm) in 2 ml tubes (Eppendorf, Hamburg, Germany). DNA was isolated using DNeasy Plant Mini Kit (Qiagen). PCR was performed using GoTaq Long PCR Master Mix (Promega, Mannheim, Germany) and published LanFTc1_INDEL2 primers (Taylor et al., 2019), provided here for reference in Supplementary Table 2. PCR conditions were as follows: initial denaturation (94°C for 2 min), then 35 cycles composed of denaturation (94°C for 30 s), annealing (62°C for 30 s), and elongation (72°C for 5 min), followed by the final extension (72°C for 10 min). Products were resolved by agarose gel electrophoresis and SYBR Safe DNA staining (Invitrogen, Carlsbad, CA, United States) and visualized on UV-transilluminator (Uvitec, Thermo Fisher Scientific, Waltham, MA, United States). Wild P27255 allele (*ku*, without deletion) was encoded as “A,” Palestinian allele (*Pal*, 1208 bp deletion) as “B,” 83A:476 allele (*Ku*, 1423 bp deletion) as “C,” and Krasnolistny allele (*Jul*, 5162 bp deletion) as “D.”

### Evaluation of Vernalization Responsiveness in Greenhouse

Vernalization was carried out by placing imbibed seeds for 21 days at 5°C in darkness on moist filter paper in Petri dishes. Non-vernalized control plants were sown five days before the end of the vernalization procedure and kept at ∼21°C to maintain a similar thermal time (Huyghe, 1991). Plants were cultivated in a greenhouse maintained by the Institute of Plant Genetics, the Polish Academy of Sciences, Poznań, Poland (52°26′N 16°54′E) during growing seasons of 2014 (sowing of vernalized plants on 14.05) and 2015 (sowing of vernalized plants on 25.03) under ambient long day photoperiod (12–17 h). The greenhouse was equipped with automatic heating to keep the minimum air temperature above 18°C. Passive cooling was maintained by a temperature-dependent ventilation system (activated at 22°C). Air temperature (daily mean and maximum) and daily sunshine hours recorded by the nearby localized meteorological station (Poznań-Ławica, 5.1 km) as well as the theoretical photoperiod hours calculated for this latitude (covering 120 days from sowing date for both years) were provided for reference in Supplementary Tables 3, 4. Flowering time was recorded as the number of days from sowing date of vernalized plants until the first fully colored petal was observed. The average number of plants sampled in 2014 was 5.6 for the non-vernalized variant (min. 3, max. 6) and 6.9 for the vernalized variant (min. 4, max. 7), whereas in 2015 it was 4.7 for the non-vernalized variant (min. 4, max. 5) and 5.0 for the vernalized variant.

### Controlled Environment Experiment for Gene Expression Profiling

Based on the results of *LanFTc1* allele genotyping and vernalization responsiveness phenotyping, three accessions were selected for gene expression profiling: P27255 from Morocco (96,234, carrying wild allele *ku*), 83A:476 from Australia (96,233, carrying domesticated allele *Ku*), and Palestyna from Palestine (95,799, carrying intermediate allele *Pal*). The vernalization of lines was performed as described above. Non-vernalized control plants were sown five days before the end of the vernalization procedure. Plants from both variants were cultivated in climatic chambers with controlled humidity (40–50% day, 70–80% night) and temperature (22°C day, 18°C night). Two types of photoperiods were studied, short day (SD, 8 h, from 8 AM to 4 PM) and long day (LD, 16 h, from 4 AM to 8 PM). Young leaves from five biological replicates were sampled every week at two times of day to follow a circadian rhythm, at 9 AM, and 3 PM under SD or at 7 AM and 6 PM under LD. Plant material was immediately frozen in liquid nitrogen and stored at −80°C. Three replicates with similar plant phenology (growth rate and time to flowering) were subjected to gene expression profiling. Taking into consideration observed flowering time, from 2 to 4 terms were selected for gene expression, representing the period from about 2 weeks before flowering to the flowering date and/or few days after flowering (Supplementary Table 5).

### RNA Isolation and cDNA Synthesis

Frozen young leaf tissue (50 mg) was homogenized using TissueLyser II (Qiagen) and two stainless steel beads (ø 5 mm) in 2 ml tubes (Eppendorf). RNA isolation was performed using the SV Total RNA Isolation System (Promega) without any alterations to the protocol. RNA concentration and purity were measured using NanoDrop 2000 (Thermo Fisher Scientific) and A260/A280 ratio. RNA integrity was visualized by 1% agarose gel electrophoresis of denatured samples in 1 × TAE buffer. RNA concentration was equalized to 1000 ng/μl in nuclease-free water. First-strand cDNA synthesis was performed using GoScript (TM) Reverse Transcription System (Promega) and 5 μg of total RNA per sample.

### Selection of Genes for Quantitative PCR

The set of genes selected for quantitative PCR included, among others, four *L. angustifolius* representatives of *FT* clade, namely *LanFTa1* (Lup021189, XM_019571501.1), *LanFTa2* (XM_019596455.1), *LanFTc1* (Lup015264, XM_019601808.1), and *LanFTc2* (Lup005674, XM_019565316.1) (Ksia̧żkiewicz et al., 2016; Nelson et al., 2017). First names in parentheses correspond to gene names provided in the *L. angustifolius* pseudochromosome assembly paper (Hane et al., 2017), whereas the second names address *L. angustifolius* NCBI Reference Sequences LupAngTanjil_v1.0. Moreover, our assay endeavored also some candidate genes revealed by recent *L. angustifolius* (eQTL mapping) study (Plewiński et al., 2019) to be associated with the vernalization response, as follows: *LanUGT85A2* (Lup002110, XM_019574900.1), *LanCRLK1* (Lup011808, XM_019603391.1), *LanAGL8* (Lup018485, XM_019583439.1), and *LanFD* (Lup018024, XM_019567853.1). Alignment of coding sequences to the *L. angustifolius* genome assembly provided evidence that *LanAGL8* and *LanFD* are present in single copies.

Moreover, some major *A. thaliana* components of the flowering induction pathway were considered, as follows: *LEAFY* (*LFY*, AT5G61850), *APETALA1* (*AP1*, AT1G69120), *VERNALIZATION INSENSITIVE 3* (*VIN3*, AT5G57380), and *VERNALIZATION 5* (*VRN5*, AT3G24440). Multiple sequence alignment revealed that all these genes have putatively three copies in the *L. angustifolius* genome, namely Lup006312 (XM_019602464.1), Lup012189 (XM_019558971.1) and Lup027481 (XM_019607325.1) for *LFY*; Lup021855 (XM_019605469.1), Lup024348 (XM_019588203.1) and Lup006876 (XM_019572960.1) for *AP1*; Lup009440 (XM_019586787.1), Lup013437 (XM_019598860.1) and Lup026125 (XM_019608083.1) for *VIN3*; and Lup009144 (XM_019591910.1), Lup018692 (XM_019567058.1) and Lup032778 (XM_019590742.1) for *VRN5*. Analysis of leaf transcriptome data obtained for the *L. angustifolius* linkage mapping population, covering developmental phases from juvenile to generative growth after partial vernalization (Plewiński et al., 2019), revealed negligible expression of all *LFY* and *AP1* homologs (∼0.07 reads per kilobase million, RPKM), very low expression of *VRN5* (∼0.56 RPKM) and high expression of VIN3 copies (∼25.07 RPKM) (Supplementary Table 6). Therefore, *VRN5* and *VIN3* homologs were selected for the expression assay, whereas *LFY* and *AP1* homologs were discarded. Reference genes validated in previous *L. angustifolius* quantitative gene expression studies were selected for this assay, namely *LanDExH7* (Lup023733, XM_019579367.1), and *LanTUB6* (Lup032899, XM_019581544.1) (Taylor et al., 2016, 2019; Nelson et al., 2017). Primers were designed in Geneious Prime (Auckland, New Zealand) using Primer3 (Kearse et al., 2012; Untergasser et al., 2012). Due to the high similarity between particular copies, all three *VRN5* homologs were analyzed together using one primer pair. The remaining genes were profiled on one by one basis. Designed primers and expected product sizes are provided in Supplementary Table 2.

### Quantitative Gene Expression Analysis

In prior experiments, a CFX Connect Real-Time PCR Detection System (Bio-Rad Polska, Warsaw, Poland) was calibrated using Melt Calibration Kit (Bio-Rad Polska) according to the manufacturer’s protocol. A standard curve was developed to assess the performance of the quantitative PCR assay and its dynamic range following recent recommendations (Svec et al., 2015). Analyzed genes were amplified using GoTaq G2 Flexi DNA Polymerase (Promega) and subjected to 1% agarose gel electrophoresis. Amplicons were excised from a gel, extracted with the aid of QIAquick Gel Extraction Kit (Qiagen), quantified using NanoDrop 2000 (Thermo Fisher Scientific), and outsourced (Genomed Ltd., Warsaw, Poland) for direct Sanger sequencing on ABI PRISM 3130 Genetic Analyzer XL (Applied Biosystems, Hitachi). A series of dilutions in concentrations ranging from 1 to 10^–10^ of the original templates were prepared for every gene using an initial volume of 20 μl to reduce the sampling error. 6 replicates per each concentration were performed using the iTaq Universal SYBR Green Supermix (Bio-Rad Polska). A two-step PCR protocol was exploited according to the protocol. A calculation of *R*^2^ and PCR efficiency values was done in Bio-Rad CFX Manager 3.1. The values obtained are provided in Supplementary Table 7.

The quantitative PCR analysis of gene expression was performed using 96-well PCR plates and two reference genes (*LanDExH7* and *LanTUB6*). Inter-run calibration sample (*LanTUB6*) and no template control were used on all plates. Three biological replicates were analyzed for each time point, and all samples were run in 3 technical repeats. High-resolution PCR product melting in the range of temperature from 65 to 85°C was performed after PCR to control the specificity of amplification. Melt profiles were inspected for the amplification of unspecific products, highlighted by the presence of melting peaks at different temperatures than those obtained during standard curve preparation. Calculations of ΔΔCq were performed in Bio-Rad CFX Manager 3.1 taking into consideration PCR efficiency values and results obtained for both reference genes. The final computations (mean value and standard deviation) and visualization (graphs) were performed in Microsoft Excel 2010.

### Statistical Analysis

Calculations were performed to check the influence of circadian clock (expression in the evening divided by expression in the morning), growth phase (maximum expression at analyzed date divided by maximum expression at the first date), vernalization (fold change of expression after vernalization), and genotype (comparison of expression levels observed in studied lines, including *Ku*/*Pal*, *Ku*/*ku*, and *Pal*/*Ku*, performed for all data points). The values obtained are provided in Supplementary Data Sheet 1. The statistical significance of these quotients was tested using a t test for the mean ratio as proposed by Hauschke et al. (1999), Tamhane and Logan (2004)). Calculations were made in R (R Core Team, 2013) with custom script using “t.test.ratio” function from the ratios package. In the first step, the equal variance was tested. If this condition was satisficed classical t-test formula was used, otherwise, Welch’s *t*-test formula was used (Welch, 1947). *P*-values were rounded up to four decimal places and are provided in the Supplementary Data Sheet 1.

## Results

### European Lupin Gene Bank Preserves the *L. angustifolius* Donors of Four Alleles of LanFTc1 Promoter

**FIGURE 1:**
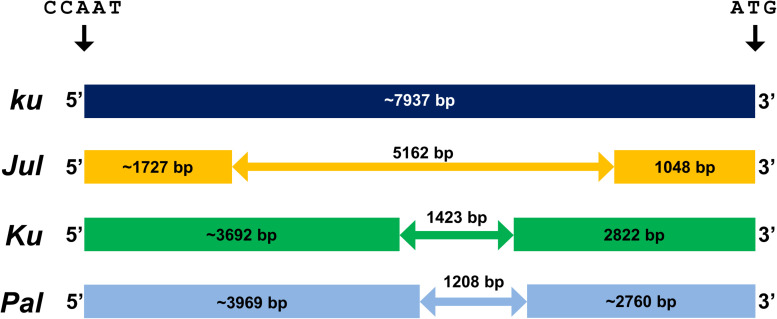
Indel variation in the promoter sequence of *LanFTc1* gene controlling flowering induction in *L. angustifolius*. Allele *ku* is typical for wild populations, alleles *Jul* and *Ku* are present only in domesticated germplasm, whereas allele *Pal* was found only in wild germplasm from Palestine. The position is given in relation to the first nucleotide of CCAAT-box (Ksia̧żkiewicz et al., 2016; Nelson et al., 2017; Taylor et al., 2019).

Indel variation in the promoter region of the major flowering time gene *LanFTc1* (Figure 1) was recently revealed to be associated with flowering time and vernalization responsiveness in *L. angustifolius* (Nelson et al., 2017; Taylor et al., 2019). To evaluate allelic composition in germplasm exploited by European lupin breeders, ninety-two *L. angustifolius* accessions, encompassing 43 primitive populations or landraces, 23 cross derivatives or breeding lines, 25 cultivars, and one mutant, derived from the European Lupin Gene Resources Database, were screened with primers flanking polymorphic *LanFTc1* promoter region (Table 1). As expected, wild *LanFTc1* allele (*ku*) was found mainly in primitive accessions collected in the Mediterranean Basin as well as in a few old domesticated materials originating from Russia, Poland, Germany, and the Republic of South Africa. One wild population line, Palestyna originating from Palestine, was found to carry the shortest variant of deletion (1208 bp), named here as *Pal*. Alleles *Ku* (1423 bp deletion) and *Jul* (5162 bp deletion) were found only in domesticated germplasm – the first one was present mostly in released cultivars, whereas the second one was typically in breeding materials at a different stage of improvement. Accessions carrying *Ku* originated primarily from Australia, Germany, and Poland, whereas those carrying *Jul* from Poland and Russia. Results of marker screening are provided in Supplementary Table 8.

**TABLE 1:** Distribution of *LanFTc1* alleles across wild and domesticated *L. angustifolius* germplasm.

*LanFTc1* allele	Lines	XC	CV	MU	WP
*ku*, without deletion	48	3	3	–	42
*Pal*, 1208 bp deletion	1	–	–	–	1
*Ku*, 1423 bp deletion	24	7	17	–	–
*Jul*, 5162 bp deletion	19	13	5	1	–

*XC, cross derivative or breeding line; CV, cultivar; MU, mutant; WP, wild population or primitive landrace.*

### Palestinian *LanFTc1* Allele Confers Intermediate Flowering Time Moderately Responsive to Vernalization

Ninety-two *L. angustifolius* accessions with known *LanFTc1* promoter allele composition were evaluated for phenotypic response to vernalization in two consecutive years, 2014 and 2015 (Table 2). Lines carrying wild *ku* allele revealed the longest vegetative phase, ranging from 61.2 ± 0.9 to 116.2 ± 3.0 days in ‘2014 and from 79.0 ± 5.8 to 101.5 ± 3.9 days in ‘2015. These lines demonstrated also high vernalization responsiveness, highlighted by the acceleration of flowering after vernalization by 13.9 to 66.4 days in 2014, and by 11.6 to 35.6 days in 2015. The line carrying *Pal* allele showed intermediate phenology and flowered about 20-35 days quicker than the average *ku* genotype, namely after ∼53 days from sowing in ‘2014 and ∼68 days in ‘2015. The vernalization procedure advanced flowering induction in this line by about two weeks. Accessions carrying domesticated *Ku* allele were very early and started flowering after 37.2 ± 0.4 to 46.2 ± 8.9 days in 2014, and after 54.0 ± 0.0 to 59.6 ± 2.6 days in 2015. This set contained germplasm low-responsive to vernalization, which accelerated flowering time by up to ∼8.6 days, as well as some truly thermoneutral accessions, which flowered at the same time regardless of the vernalization procedure. Lines carrying domesticated European *Jul* allele also revealed very early phenology, manifested by the onset of flowering after 37.0 ± 1.3 to 41.0 ± 5.9 days in 2014, and after 52.2 ± 1.5 to 60.8 ± 1.8 days in 2015. Most *Jul* lines were fully thermoneutral. On average, *Jul* lines flowered earlier than *Ku* lines in both years, however taking into consideration standard errors resulting from variability between biological replicates, differences between mean values were not statistically significant. Results of time to flowering and vernalization responsiveness in relation to LanFTc1_INDEL2 marker polymorphism are provided in Supplementary Table 8 (2014) and Supplementary Table 9 (2015).

**TABLE 2:** Comparison of flowering time and vernalization responsiveness of *L. angustifolius* germplasm carrying *Jul*, *Ku*, *Pal*, and *ku LanFTc1* alleles, cultivated in a greenhouse under natural long days (LD).

*LanFTc1* allele	Days to flowering ‘2014	Vernalization response ‘2014	Days to flowering ‘2015	Vernalization response ‘2015
*ku*, without deletion	88.8 ± 15.6^a^	−41.1 ± 13.8	88.0 ± 5.9	−21.8 ± 6.0
*Pal*, 1208 bp deletion	53.5 ± 1.9	−14.5 ± 2.7	68.2 ± 5.6	−14.8 ± 3.4
*Ku*, 1423 bp deletion	40.5 ± 2.6	−3.3 ± 2.6	56.7 ± 2.1	−3.0 ± 2.5
*Jul*, 5162 bp deletion	38.8 ± 1.1	−0.2 ± 2.2	55.6 ± 1.9	−2.2 ± 2.2

*^a^Standard deviation.*

Based on the results of LanFTc1_INDEL2 marker screening and vernalization responsiveness, three lines were selected for vernalization response phenotyping under 8-h (SD) and 16-h (LD) photoperiods: 84A:476 carrying domesticated *Ku* allele (early flowering, thermoneutral), Palestyna carrying wild *Pal* allele (moderately flowering and responsive to vernalization), and P27255 carrying wild *ku* allele (late flowering and highly responsive to vernalization). 83A:476 was revealed to be the earliest line in both photoperiods, followed by Palestyna (Table 3). Under SD, 83A:476 accelerated transition between phases in response to vernalization by about 5 days, whereas under LD by about 3 days. These responses were higher in Palestyna, amounting to about 12-19 days and 3-5 days, respectively. P27255 did not flower during the experiment (90 days) in all variants except vernalized plants under LD.

**TABLE 3:** Number of days to first bud, flower, and pod in *L. angustifolius* germplasm carrying *Ku*, *Pal*, and *ku LanFTc1* alleles, cultivated under 8- and 16-h photoperiods.

*LanFTc1* allele	Vernalization variant	Days to first bud	Days to first flower	Days to first pod
**8-h photoperiod**				
*Ku*	-	51.3 ± 1.6^a^	57.2 ± 4.3	63.3 ± 9.8
	+	45.7 ± 2.9	52.0 ± 4.2	58.1 ± 2.5
*Pal*	-	65.3 ± 3.7	71.2 ± 3.8	83.5 ± 2.7
	+	52.9 ± 3.0	55.9 ± 2.6	64.7 ± 4.6
*ku*	-	–	–	–
	+	–	–	–
**16-h photoperiod**				
*Ku*	-	33.6 ± 1.1	38.7 ± 1.3	44.1 ± 1.4
	+	29.8 ± 2.7	35.3 ± 1.0	41.2 ± 1.0
*Pal*	-	36.7 ± 0.5	43.8 ± 0.4	49.0 ± 0.5
	+	40.0 ± 0.0	48.4 ± 1.4	52.0 ± 0.8
*ku*	-	–	–	–
	+	55.1 ± 1.3	59.0 ± 1.2	69.0 ± 2.4

*^a^Standard deviation.*

### *LanFTc1* Expression Was High and Thermoneutral in *Ku*, High and Partially Vernalization-Independent in *Pal*, Whereas Low and Positively Responsive to Vernalization in *ku*

83A:476 (*Ku*), Palestyna (*Pal*), and P27255 (*ku*) grown under SD and LD conditions were used for gene expression profiling (Supplementary Data Sheet 1). The vernalization responsiveness of analyzed genes was calculated as a mean fold change of expression in vernalized plants compared to non-vernalized control (averaged across all day terms and dates measured for a particular line), as summarized in Table 4. The circadian clock responsiveness were calculated as a mean fold change of expression occurring between the morning and the evening terms (averaged across all dates measured for a particular line) and are provided in Table 5. The trend in expression level during plant growth was calculated as a fold change of expression occurring between the first and the last term (based on maximum daily values) and is provided in Table 6.

**TABLE 4:** Vernalization responsiveness of analyzed genes in *L. angustifolius* germplasm carrying *Ku*, *Pal*, and *ku* alleles, cultivated under 8-h (SD) and 16-h (LD) photoperiods.

Gene name	Mean change of expression after vernalization under SD			Mean change of expression after vernalization under LD		
	*Ku*	*Pal*	*ku*	*Ku*	*Pal*	*ku*
*LanAGL8*	1.6^a^	2.2	13.0	1.8	6.1	138.6
*LanCRLK1*	0.7	0.6	2.6	0.6	1.3	1.0
*LanFD*	0.5	0.5	3.6	0.3	0.7	1.0
*LanFTa1*	9.9	1.3	0.9	1.8	2.1	14.7
*LanFTa2*	1.1	0.3	13.3	3.2	1.1	1.2
*LanFTc1*	1.3	0.9	4.3	1.3	3.6	144.4
*LanFTc2*	0.7	0.7	3.5	1.1	3.2	1.4
*LanUGT85A2*	0.3	0.2	0.4	0.2	0.2	0.4
*LanVIN3-1*	0.9	0.9	1.9	0.8	1.0	1.1
*LanVIN3-2*	0.7	1.1	2.3	0.7	1.3	1.6
*LanVIN3-3*	0.9	1.7	2.3	0.6	1.3	1.0
*LanVRN5*	0.8	1.0	2.3	0.9	1.0	0.8

*^a^Fold change of expression occurring in response to vernalization, averaged across all data points (day terms and dates).*

**TABLE 5:** Circadian clock responsiveness of analyzed genes in *L. angustifolius* germplasm carrying *Ku*, *Pal*, and *ku* alleles, cultivated under 8-h (SD) and 16-h (LD) photoperiods without vernalization and with vernalization.

Gene name	Mean change of expression during light phase under SD			Mean change of expression during light phase under LD		
	*Ku*	*Pal*	*ku*	*Ku*	*Pal*	*ku*
*LanAGL8*	1.6 | 1.0^a^	0.3 | 0.7	0.8 | 0.7	2.6 | 1.6	1.3 | 3.6	2.0 | 0.5
*LanCRLK1*	0.9 | 1.0	0.7 | 1.0	0.8 | 0.7	0.8 | 0.5	0.8 | 1.3	1.0 | 1.0
*LanFD*	0.3 | 0.8	1.7 | 3.1	1.2 | 1.6	0.3 | 0.4	1.6 | 2.4	1.9 | 1.3
*LanFTa1*	1.0 | 1.7	0.6 | 0.6	0.7 | 0.8	0.8 | 2.7	0.8 | 4.5	0.8 | 0.7
*LanFTa2*	0.6 | 0.9	0.9 | 0.4	0.4 | 0.4	0.4 | 2.2	0.7 | 0.4	0.6 | 0.6
*LanFTc1*	4.8 | 3.1	0.5 | 0.8	1.2 | 2.4	1.8 | 2.9	1.2 | 1.1	2.7 | 4.3
*LanFTc2*	2.0 | 4.3	2.0 | 11.1	5.6 | 24.3	2.7 | 1.7	2.0 | 0.5	3.4 | 4.2
*LanUGT85A2*	5.0 | 3.4	2.6 | 2.1	2.4 | 2.4	3.2 | 4.6	3.8 | 2.2	3.3 | 2.9
*LanVIN3-1*	1.2 | 1.5	0.7 | 1.1	1.4 | 0.9	1.4 | 1.4	1.9 | 2.7	1.5 | 1.5
*LanVIN3-2*	1.9 | 1.2	1.0 | 1.2	1.3 | 0.9	1.7 | 1.4	1.5 | 2.4	1.4 | 1.4
*LanVIN3-3*	1.4 | 1.3	0.8 | 1.0	1.2 | 0.9	2.1 | 1.5	1.5 | 2.0	1.4 | 1.7
*LanVRN5*	0.7 | 0.8	0.7 | 0.7	0.7 | 0.7	1.3 | 0.9	1.1 | 0.9	1.0 | 0.9

*^a^Fold change of expression occurring between the morning and the evening terms, averaged across all dates; the first number corresponds to the experiment without vernalization, the second number to the experiment with vernalization.*

**TABLE 6:** Change of expression level of analyzed genes in *L. angustifolius* germplasm carrying *Ku*, *Pal*, and *ku* alleles, during plant growth under 8-h (SD) and 16-h (LD) photoperiods.

Gene name	Change of expression during experiment under SD			Change of expression during experiment under LD		
	*Ku*	*Pal*	*ku*	*Ku*	*Pal*	*ku*
*LanAGL8*	1.5 | 2.3^a^	3.5 | 6.3	0.4 | 6.4	1.6 | 1.0	16.6 | 6.8	4.0 | 805.9
*LanCRLK1*	0.5 | 1.2	0.9 | 1.2	0.6 | 0.5	0.8 | 0.8	0.6 | 2.7	0.6 | 1.2
*LanFD*	0.2 | 0.6	0.7 | 1.2	0.4 | 1.9	0.4 | 0.2	0.3 | 0.4	1.7 | 4.7
*LanFTa1*	0.7 | 1.3	1.7 | 5.6	0.5 | 1.4	23.6 | 7.3	404.5 | 340.2	2.2 | 765.0
*LanFTa2*	0.2 | 0.4	7.6 | 1.6	0.09 | 9.2	0.05 | 0.5	0.4 | 1.9	0.2 | 2.6
*LanFTc1*	0.4 | 1.2	0.9 | 3.0	0.08 | 0.3	1.5 | 2.2	15.7 | 10.2	0.7 | 118.8
*LanFTc2*	0.4 | 1.5	1.2 | 2.5	0.5 | -	1.1 | 0.9	3.8 | 5.9	0.7 | 1.1
*LanUGT85A2*	0.1 | 0.1	0.3 | 0.3	0.4 | 1.2	0.3 | 0.1	0.02 | 0.02	0.5 | 0.08
*LanVIN3-1*	0.3 | 3.0	1.0 | 1.2	0.8 | 1.4	2.3 | 1.2	1.0 | 1.1	1.0 | 1.0
*LanVIN3-2*	0.4 | 2.6	1.3 | 1.3	0.9 | 1.9	3.3 | 1.1	1.0 | 1.8	0.9 | 1.9
*LanVIN3-3*	0.2 | 3.5	1.2 | 3.2	0.4 | 1.3	2.5 | 0.9	1.2 | 1.8	0.9 | 0.9
*LanVRN5*	0.3 | 1.5	1.4 | 1.1	0.3 | 0.9	3.7 | 2.2	1.1 | 1.0	1.0 | 0.8

*^a^Fold change in expression occurring between the first and the last term, based on the maximum daily values; the first number corresponds to the experiment without vernalization, the second number to the experiment with vernalization.*

First, we analyzed the expression of the *LanFTc1* gene, which is considered as the major controller of vernalization responsiveness and early flowering in *L. angustifolius* (Nelson et al., 2017; Taylor et al., 2019). The studied genotypes showed different patterns of *LanFTc1* expression in response to photoperiod, vernalization, and circadian rhythm.

**FIGURE 2:**
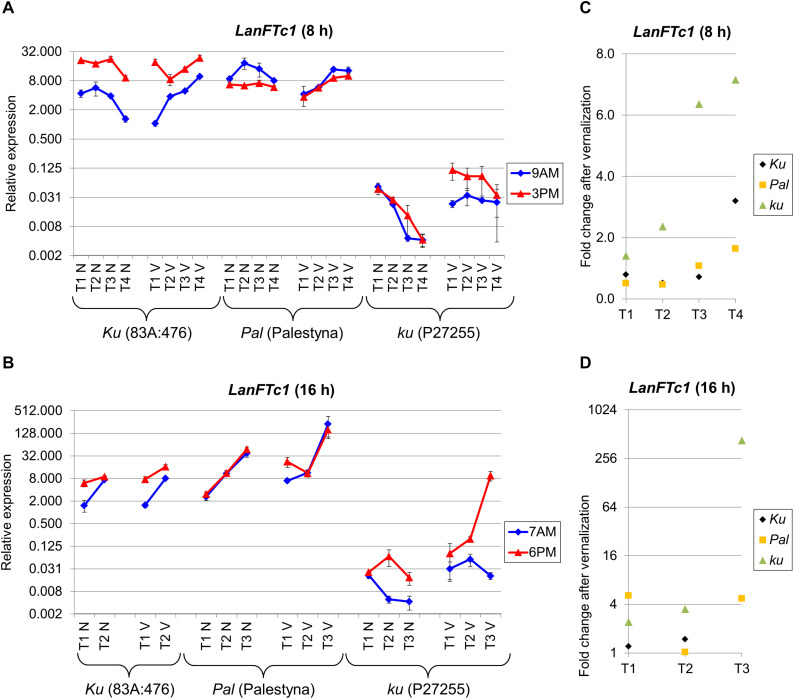
Gene expression profile of the *LanFTc1* gene in response to photoperiod and vernalization in three lines (83A:476, Palestyna, and P27255) carrying different *LanFTc1* alleles (*Ku*, *Pal*, and *ku*). **(A)** expression under an 8-h photoperiod, **(B)** expression under 16-h photoperiod, **(C)** vernalization response under 8-h photoperiod, **(D)** vernalization response under 16-h photoperiod. T1-T4 stands for sampling terms (Supplementary Table 5), V for vernalized plants, and N for non-vernalized plants. Timespan of photoperiods: 8-h from 4 AM to 8 PM, 16-h from 4 AM to 8 PM. Two references were used for normalization (*LanDExH7* and *LanTUB6*) and one sample (*LanTUB6*) for inter-run calibration. Error bars indicate a standard deviation of 3 biological replicates, each representing a mean of 3 technical replicates. A logarithmic scale was used to accommodate observed large differences in gene expression values.

Under SD, *LanFTc1* expression in non-vernalized plants was the highest in 83A:476 and the lowest in P27255 (Figure 2A). Indeed, *LanFTc1* expression in 83A:476 was up to 2.4 times higher than in Palestyna (*P* = 0.0016) and 460-2213 times higher than in P27255 (*P* = 0.0000). After vernalization, *LanFTc1* expression in 83A:476 was up to 4.5 times higher than in Palestyna (*P* = 0.0064) and up to 686 times higher than in P27255 (*P* = 0.0029). The difference of *LanFTc1* expression between 83A:476 and P27255 increased during plant development in both vernalization variants, whereas for the pair 83A:476 and Palestyna it was decreasing. Under LD, *LanFTc1* expression was the highest in Palestyna and the lowest in P27255 (Figure 2B). Namely, in non-vernalized plants, expression in Palestyna was 1.8-5.4 times higher than in 83A:476 (*P* = 0.0125) and 121-2642 times higher than in P27255 (P = 0.0085), whereas in vernalized plants it was 1.5-13.8 times higher in Palestyna than in 83A:476 (not significant, NS) and 24-276 times higher than in P27255 (*P* = 0.0094). The difference to Palestyna was increasing during plant growth for 83A:476 in both vernalization variants and P27255 in the absence of vernalization.

Vernalization influence on *LanFTc1* expression in P27255 was manifested by up to a 7.2-fold increase under SD (NS) (Figure 2C) and up to 427-fold increase under LD (*P* = 0.0125) (Figure 2D). The vernalization effect under SD was changing in 83A:476 from a 0.5-fold decrease (*P* = 0.0115) to a 3.2-fold increase (*P* = 0.0094) and similarly in Palestyna from a 0.5-fold decrease (*P* = 0.0291) to 1.6-fold increase (NS). This effect under LD was neutral in 83A:476 and positive in Palestyna, up to 5.1-fold increase (*P* = 0.0351).

The circadian clock regulation differed between genotypes and partially between environments. In 83A:476, *LanFTc1* expression was generally higher in the evening than in the morning in all combinations of the photoperiod and vernalization (up to 18.4-fold increase, *P* = 0.0008). In Palestyna, *LanFTc1* expression under SD was usually higher in the morning (up to 2.9-fold increase, *P* = 0.0172), whereas under LD this effect was variable. In P27255, *LanFTc1* expression was higher in the evening, especially under LD after vernalization (*P* = 0.0046).

### *LanFTa1* Expression Was Low and Uniform in All Lines Under SD, However, It Was Highly Induced Just Before Flowering, Especially in *Pal* and *ku* Genotypes Under LD

**FIGURE 3:**
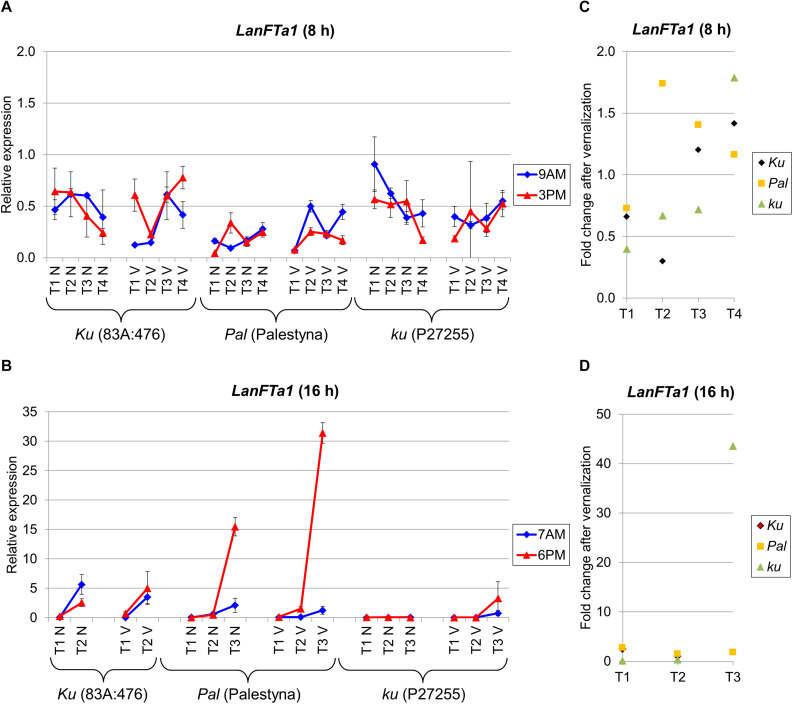
Gene expression profile of the *LanFTa1* gene in response to photoperiod and vernalization in three lines (83A:476, Palestyna, and P27255) carrying different *LanFTc1* alleles (*Ku*, *Pal*, and *ku*). **(A)** expression under 8-h photoperiod, **(B)** expression under 16-h photoperiod, **(C)** vernalization response under 8-h photoperiod, **(D)** vernalization response under 16-h photoperiod. T1-T4 stands for sampling terms (Supplementary Table 5), V for vernalized plants, and N for non-vernalized plants. Timespan of photoperiods: 8-h from 4 AM to 8 PM, 16-h from 4 AM to 8 PM. Two references were used for normalization (*LanDExH7* and *LanTUB6*) and one sample (*LanTUB6*) for inter-run calibration. Error bars indicate a standard deviation of 3 biological replicates, each representing a mean of 3 technical replicates.

Besides *LanFTc1*, three other *FT* genes, namely *LanFTa1*, *LanFTa2*, and *LanFTc2*, were analyzed to complement our perspective on *FT* clade transcriptional activity in *L. angustifolius* response to major environmental cues. *LanFTa1* gene expression under SD did not reveal any significant trend during plant growth and differences between genotypes were also usually not significant (Figure 3A). The relative level of expression was rather low (mean 0.38). A different pattern was observed under LD, when the expression was highly induced before flowering compared to the first term, namely 24-fold (*P* = 0.045) in non-vernalized 83A:476 (7-fold in vernalized, NS), 405-fold (*P* = 0.0051) in non-vernalized Palestyna (340-fold in vernalized, *P* = 0.0015), and 765-fold in vernalized P27255 (Figure 3B). In non-vernalized P27255 *LanFTa1* expression remained at a low level but this genotype did not flower in such conditions.

The vernalization effect on *LanFTa1* expression under LD in 83A:476 and Palestyna was slightly positive, up to 2.5-fold (NS) and 2.8-fold (*P* = 0.0011) increase, respectively (Figures 3C,D). In P27255, the vernalization influence was initially moderately negative (0.14-fold and 0.35-fold decrease, *P* = 0.0002) but became highly positive just before flowering (43.6-fold increase). The circadian regulation was not stable across terms and genotypes, however, when *LanFTa1* was induced before flowering in Palestyna under LD, its expression was significantly higher in the evening than in the morning (up to 25.1-fold increase, *P* = 0.0004).

### *LanFTa2* and *LanFTc2* Expression Was Low in Both Photoperiods in All Lines

*LanFTa2* gene expression was very low in both photoperiods, amounting to mean values of 0.095 under SD and 0.004 under LD (Supplementary Figures 1A,B). There was remarkable induction of expression in P27255 after vernalization under SD (up to 38-fold, *P* = 0.0002), however, the relative expression values achieved were much lower than those observed for *LanFTc1* and *LanFTa1* (Supplementary Figures 1C,D). This pattern was not recreated under LD.

*LanFTc2* expression was on a low level in all lines under both photoperiods (Supplementary Figures 2A,B). A moderate induction by vernalization in P27255 under SD (up to 7.3-fold increase, *P* = 0.0187) and in Palestyna under LD (up to 4.7-fold increase, *P* = 0.0023) was observed but obtained levels were less than half of the mean expression obtained for control genes (Supplementary Figures 2C,D).

### *LanAGL8* Expression Profile Reflected Observed Differences in Plant Phenology and Was Similar to *LanFTc1*

**FIGURE 4:**
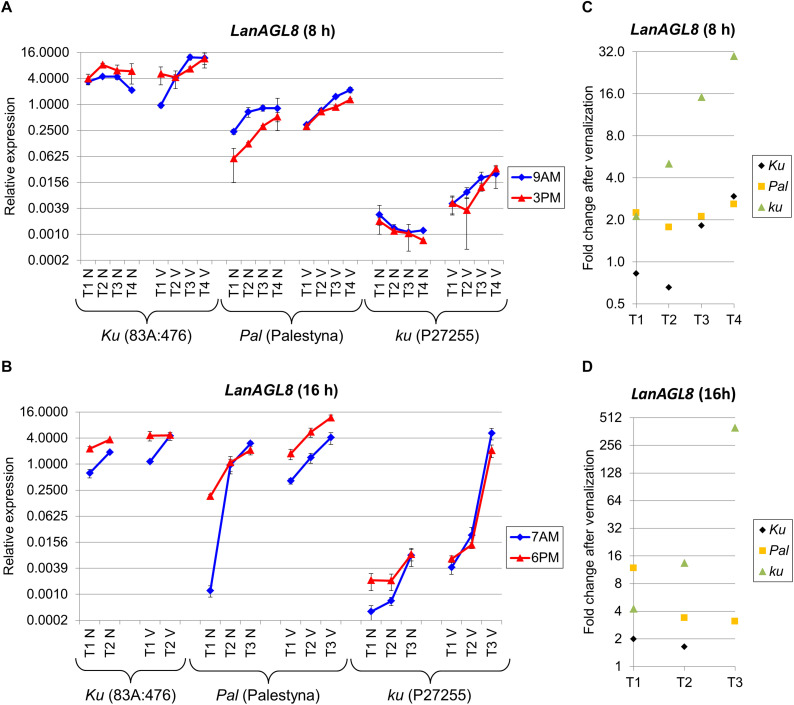
Gene expression profile of the *LanAGL8* gene in response to photoperiod and vernalization in three lines (83A:476, Palestyna, and P27255) carrying different *LanFTc1* alleles (*Ku*, *Pal*, and *ku*). **(A)** expression under 8-h photoperiod, **(B)** expression under 16-h photoperiod, **(C)** vernalization response under 8-h photoperiod, **(D)** vernalization response under 16-h photoperiod. T1-T4 stands for sampling terms (Supplementary Table 5), V for vernalized plants, and N for non-vernalized plants. Timespan of photoperiods: 8-h from 4 AM to 8 PM, 16-h from 4 AM to 8 PM. Two references were used for normalization (*LanDExH7* and *LanTUB6*) and one sample (*LanTUB6*) for inter-run calibration. Error bars indicate a standard deviation of 3 biological replicates, each representing a mean of 3 technical replicates. A logarithmic scale was used to accommodate observed large differences in gene expression values.

Besides homologs constituting the *L. angustifolius FT* clade (Ksia̧żkiewicz et al., 2016; Nelson et al., 2017), four novel candidate genes (*LanAGL8, LanFD, LanUGT85A2*, and *LanCRLK1*), recently considered to be putatively involved in *Ku*-based response (Plewiński et al., 2019), were profiled in this study. *LanAGL8* is an *L. angustifolius* homolog of the *A. thaliana FRUITFULL* gene participating in flowering time control, meristem identity, and fruit development (Mandel and Yanofsky, 1995; Gu et al., 1998). *LanAGL8* expression was consecutively increasing during plant growth for all studied combinations of lines, photoperiod, and vernalization variants except non-vernalized P27255 under SD. However, there were considerable differences in the observed expression levels between genotypes. Under SD without vernalization, expression of the *LanAGL8* gene in 83A:476 (*Ku*) was approximately 7-17 times higher (*P* = 0.0028) than in Palestyna (*Pal*) and 1420-6090 times higher (*P* = 0.0035) than in P27255 (*ku*) (Figure 4A). The influence of vernalization resulted in a reduction of differences in *LanAGL8* expression between 83A:476 and Palestyna by 11–23%, and between 83A:476 and P27255 by 30–92%. Under LD without vernalization, *LanAGL8* expression in 83A:476 was higher up to 12-fold (*P* = 0.0086) than in Palestyna and up to 1805-fold (*P* = 0.0001) than in P27255 (Figure 4B). The application of vernalization reduced these differences considerably. To summarize, Palestyna revealed an intermediate *LanAGL8* expression profile, however, it was much more like 83A:476 than P27255.

The *LanAGL8* gene revealed high positive responsiveness to vernalization in P27255 in both photoperiods, and this effect was consecutively increasing during plant growth until flowering. Namely, the change of *LanAGL8* expression in P27255 after vernalization was raised from 2.1-fold (NS) to 29.8-fold (*P* = 0.0175) under SD (Figure 4C), and from 4.3-fold (*P* = 0.0121) to 398.2-fold (*P* = 0.0004) under LD (Figure 4D). Vernalization was also inductive in Palestyna, providing an up to 2.6-fold increase (*P* = 0.0329) of *LanAGL8* expression under SD (effect stable across sampling terms) and up to 11.8-fold increase (*P* = 0.0297) under LD (effect consecutively decreasing). In the 83A:476 line, the influence of vernalization was the lowest, yielding up to a 2.9-fold increase of *LanAGL8* expression under SD (*P* = 0.0066) and up to a 2.0-fold increase under LD (*P* = 0.0315).

The *LanAGL8* gene revealed diversified circadian clock regulation between genotypes and environments. Under SD, its expression was higher in the morning than in the evening in Palestyna and P27255, whereas in 83A:476 this relation was the opposite. Moreover, vernalization partially diminished these differences. Under LD, *LanAGL8* expression was usually higher in the evening terms for all lines, however, during the growth of vernalized P27255 this trend reversed.

### *LanCRLK1* Expression Was Higher Under SD in All Lines and Negatively Responsive to Vernalization in Domesticated *Ku* Line

**FIGURE 5:**
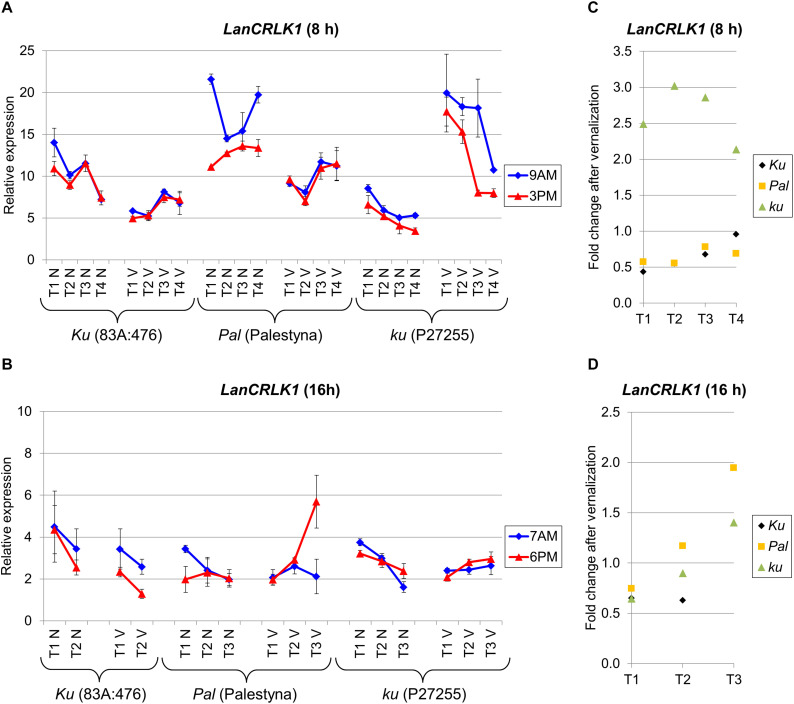
Gene expression profile of the *LanCRLK1* gene in response to photoperiod and vernalization in three lines (83A:476, Palestyna, and P27255) carrying different *LanFTc1* alleles (*Ku*, *Pal*, and *ku*). **(A)** expression under 8-h photoperiod, **(B)** expression under 16-h photoperiod, **(C)** vernalization response under 8-h photoperiod, **(D)** vernalization response under 16-h photoperiod. T1-T4 stands for sampling terms (Supplementary Table 5), V for vernalized plants, and N for non-vernalized plants. Timespan of photoperiods: 8-h from 4 AM to 8 PM, 16-h from 4 AM to 8 PM. Two references were used for normalization (*LanDExH7* and *LanTUB6*) and one sample (*LanTUB6*) for inter-run calibration. Error bars indicate a standard deviation of 3 biological replicates, each representing a mean of 3 technical replicates.

*LanCRLK1* is an *L. angustifolius* homolog of the *A. thaliana CRLK1* gene which participates in response to low temperature in a calcium-dependent manner (Yang et al., 2010). Changes in *LanCRLK1* expression level were usually not related to the progress of plant growth, except P27255 cultivated under SD, which revealed a stable decrease of *LanCRLK1* both in non-vernalized and vernalized variants. The expression of *LanCRLK1* was higher under SD than under LD. Moreover, under SD without vernalization, *LanCRLK1* expression was the highest in Palestyna (up to 2.7-fold increase compared to 83A:476, *P* = 0.0002) and the lowest in P27255 (about 0.4-fold decrease, *P* = 0.0099). However, after vernalization, expression was highest in P27255 (up to a 3.5-fold increase compared to 83A:476, *P* = 0.0005) and lowest in 83A:476 (Figure 5A). The differences in the *LanCRLK1* expression between genotypes under LD were generally not significant (Figure 5B).

The effect of vernalization on *LanCRLK1* expression under SD was negative (from 0.4-fold to 0.7-fold decrease, *P* = 0.0009) or neutral in 83A:476, negative in Palestyna (from 0.6-fold to 0.8-fold decrease, *P* = 0.0000), and positive in P27255 (up to 3.0 fold increase, *P* = 0.0000) (Figure 5C). Under LD, the vernalization effect was also negative in 83A:476 (0.6-fold decrease, NS), however, in the remaining two lines, the effect changed during plant growth from negative to positive: from a 0.7-fold decrease to 1.9-fold increase in Palestyna (all NS) and from 0.6-fold decrease (*P* = 0.0001) to 1.4-fold increase in P27255 (*P* = 0.0107) (Figure 5D). The influence of the circadian clock on *LanCRLK1* expression was variable in both photoperiods and usually not significant.

### *LanFD* Expression Was Negatively Responsive to Vernalization in *Ku* and *Pal* Lines but Positively Responsive or Variable in *ku*

**FIGURE 6:**
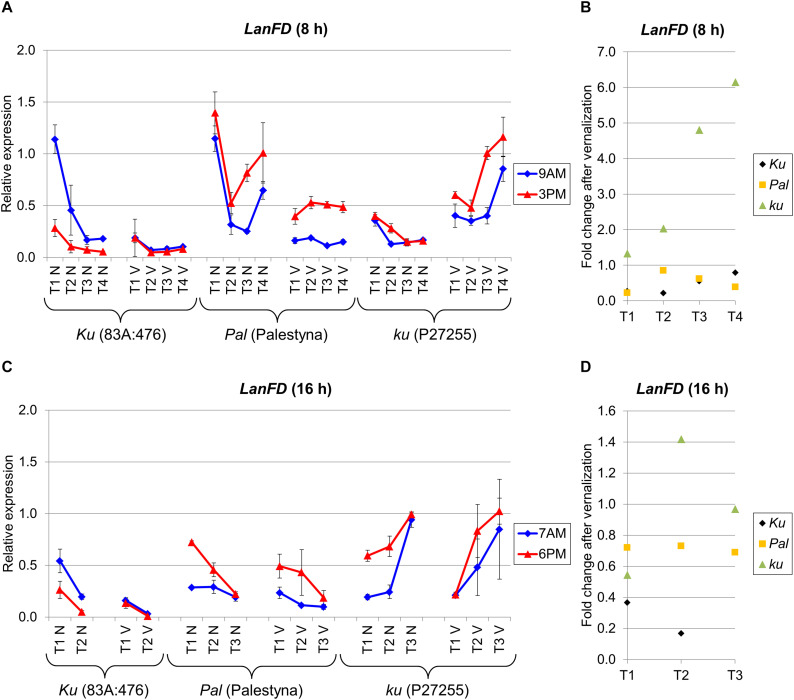
Gene expression profile of the *LanFD* gene in response to photoperiod and vernalization in three lines (83A:476, Palestyna, and P27255) carrying different *LanFTc1* alleles (*Ku*, *Pal*, and *ku*). **(A)** expression under 8-h photoperiod, **(B)** expression under 16-h photoperiod, **(C)** vernalization response under 8-h photoperiod, **(D)** vernalization response under 16-h photoperiod. T1-T4 stands for sampling terms (Supplementary Table 5), V for vernalized plants, and N for non-vernalized plants. Timespan of photoperiods: 8-h from 4 AM to 8 PM, 16-h from 4 AM to 8 PM. Two references were used for normalization (*LanDExH7* and *LanTUB6*) and one sample (*LanTUB6*) for inter-run calibration. Error bars indicate a standard deviation of 3 biological replicates, each representing a mean of 3 technical replicates. A logarithmic scale was used to accommodate observed large differences in gene expression values.

*LanFD* is an *L. angustifolius* homolog of the *A. thaliana FD* gene which triggers flowering based on FT-mediated signaling (Abe et al., 2005). *LanFD* expression in non-vernalized plants was usually the highest at the first sampling date and decreased during plant growth in both photoperiods, except in P27255 cultivated under LD. However, in vernalized plants, this decreasing trend was diminished or even reversed, as observed for P27255. Under SD, *LanFD* expression was the highest in Palestyna (1.1–5.6 times higher than in 83A:476, *P* = 0.0024) and the lowest in P27255 (0.3–0.9 times lower than in 83A:476, *P* = 0.0123) (Figure 6A). Interestingly, these relations were changed after vernalization as follows: *LanFD* expression in P27255 compared to 83A:476 was 3.2–12.2 times higher (*P* = 0.0011), whereas in Palestyna 2.1–7.4 times higher than in 83A:476 (*P* = 0.0001). Under LD without vernalization, differences in *LanFD* expression between genotypes were rather low, accounting for up to a 2.3-fold increase in Palestyna (*P* = 0.0205) and up to a 5.0-fold increase in P27255 (*P* = 0.0000) compared to 83A:476. However, vernalization exaggerated these contrasts and observed *LanFD* expression in P27255 was up to 25.7 times higher than in 83A:476 (*P* = 0.0078) and up to 5.5 times higher than in Palestyna (*P* = 0.0032) (Figure 6B).

The effect of vernalization on *LanFD* expression was negative in 83A:476 and Palestyna under both photoperiods (0.17-fold to 0.85-fold decrease), positive in P27255 under SD (up to 6.2-fold increase), and unstable in P27255 under LD (from 0.5-fold decrease to 1.4-fold increase) (Figures 6C,D). Genotypes differed in the circadian clock control of *LanFD* expression. Generally, in 83A:476 the levels were higher in the morning (up to 4.4 fold-increase), whereas in Palestine and P27255 in the evening (up to 4.5-fold and 3.1-fold increase, respectively).

### *LanUGT85A2* Expression Revealed a Growth-Dependent Decreasing Trend, Strong Circadian Clock Control, and Negative Response to Vernalization in All Lines Under Both Photoperiods

*LanUGT85A2* is considered as an *L. angustifolius* homolog of the *A. thaliana UDP-glycosyltransferase 85A2* gene. The expression of this gene was the highest at the first sampling date in all lines and consecutively decreased during the experiment. Palestyna was revealed to have the biggest decrease of *LanUGT85A2* during the transition from juvenile to generative phase, whereas P27255 had the lowest. Indeed, this decrease of *LanUGT85A2* expression during plant growth under SD reached approximately 0.1-fold for 83A:476 (*P* = 0.0114), 0.3-fold for Palestyna (*P* = 0.0033) and 0.4-fold for P27255 (*P* = 0.0003), whereas under LD about 0.3-fold for 83A:476 (*P* = 0.0501), 0.02-fold for Palestyna (*P* = 0.0182) and 0.5-fold for P27255 (*P* = 0.0196).

**FIGURE 7:**
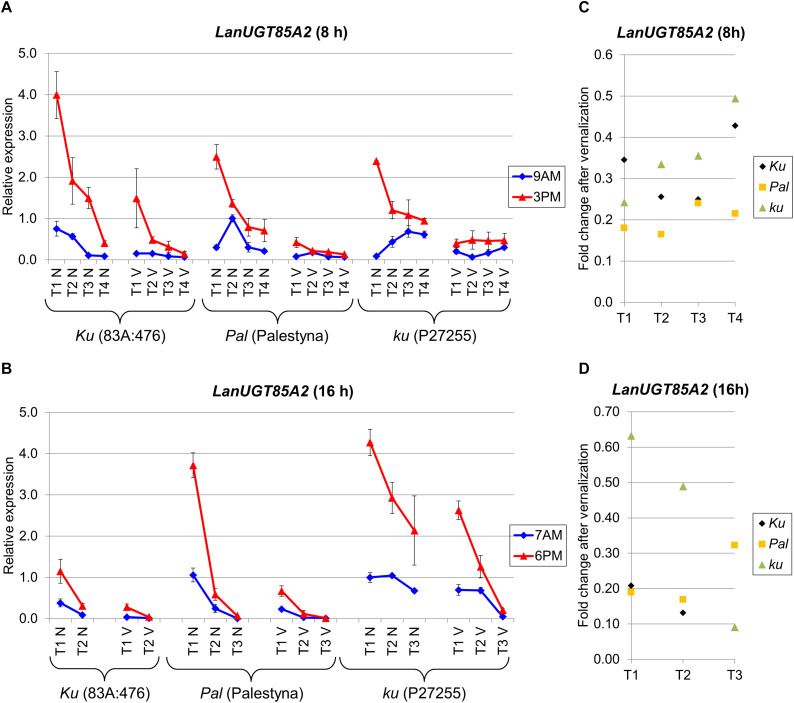
Gene expression profile of the *LanUGT85A2* gene in response to photoperiod and vernalization in three lines (83A:476, Palestyna, and P27255) carrying different *LanFTc1* alleles (*Ku*, *Pal*, and *ku*). **(A)** expression under 8-hphotoperiod, **(B)** expression under 16-h photoperiod, **(C)** vernalization response under 8-h photoperiod, **(D)** vernalization response under 16-h photoperiod. T1-T4 stands for sampling terms (Supplementary Table 5), V for vernalized plants, and N for non-vernalized plants. Timespan of photoperiods: 8-h from 4 AM to 8 PM, 16-h from 4 AM to 8 PM. Two references were used for normalization (*LanDExH7* and *LanTUB6*) and one sample (*LanTUB6*) for inter-run calibration. Error bars indicate a standard deviation of 3 biological replicates, each representing a mean of 3 technical replicates.

Under SD, the highest first-term *LanUGT85A2* expression was observed in 83A:476, about 1.7-fold higher than in Palestyna (*P* = 0.0448) and P27255 (NS) (Figure 7A). These differences were doubled by vernalization. Contrary, under LD, the highest first-term expression was in P27255, 1.1-fold higher than in Palestyna (NS) and 3.7-fold higher than in 83A:476 (*P* = 0.0006), and these differences were tripled by vernalization (Figure 7B).

Vernalization was observed to have a stable negative effect on *LanUGT85A2* gene expression, highlighted by an average 0.29-fold decrease under SD (Figure 7C) and a 0.28-fold decrease under LD (Figure 7D). Response to vernalization was the weakest in P27255 (0.38-fold) and the strongest in Palestyna (0.21-fold). Such a disproportion in vernalization response, combined with the observed difference in decreasing trend during plant growth, resulted in the highest *LanUGT85A2* expression in the P27255 line at the end of the experiment under both photoperiods (up to 31-fold increase compared to 83A:476 under LD, *P* = 0.0233).

The direction of circadian clock control was coherent across all genotypes and environments. *LanUGT85A2* expression was higher in the evening than in the morning for all terms, both with and without vernalization. Under SD, it increased during the day up to 13.9-fold in 83A:476 (*P* = 0.0165), 8.3-fold in Palestyna (*P* = 0.0075) and 28.2-fold in P27255 (*P* = 0.0000). Under LD, it increased up to 7.4-fold in 83A:476 (*P* = 0.0326), 12.8-fold in Palestyna (*P* = 0.0005) and 4.4-fold in P27255 (*P* = 0.002).

### *LanVIN3-1*, *LanVIN3-2*, and *LanVIN3-3* Genes Revealed High Expression in *Ku* Without Vernalization

The *L. angustifolius* genome contains three homologs (named here as *LanVIN3-1*, *LanVIN3-2*, and *LanVIN3-3*) a *VIN3* gene that is involved in the vernalization response in *A. thaliana* (Sung and Amasino, 2004). Therefore, the transcriptional activity of these genes was also profiled. In general, *LanVIN3-1* revealed the highest expression, whereas *LanVIN3-3* the lowest (see Supplementary Figures 3–5).

All three copies revealed differences in expression levels between genotypes, usually in descending order, 83A:476 – Palestyna – P27255. Maximum differences between 83A:476 and P27255 expression under LD reached 5.9-fold (*LanVIN3-1*, *P* = 0.0016), 25.6-fold (*LanVIN3-2*, *P* = 0.0061) and 8.4-fold (*LanVIN3-3*, *P* = 0.0059), whereas under SD these values were as follows: 4.8-fold (*LanVIN3-1*, *P* = 0.0135), 10.8-fold (*LanVIN3-2*, *P* = 0.0036) and 5.5-fold (*LanVIN3-3*, *P* = 0.0072). The expression levels of all three genes under LD were usually significantly higher in the evening than in the morning in all lines, whereas under SD, diurnal variations were frequently not significant or variable. Photoperiod conditions had a significant influence on expression changes during plant growth, especially for 83A:476. The expression levels of these genes in non-vernalized 83A:476 under LD increased by 2.3-fold (*LanVIN3-1*, *P* = 0.0008), 3.3-fold (*LanVIN3-2*, *P* = 0.0048) and 2.5-fold (*LanVIN3-3*, *P* = 0.0036) whereas under SD they decreased by up to 0.32-fold (*LanVIN3-1*, *P* = 0.0000), 0.37-fold (*LanVIN3-2*, *P* = 0.0110), and 0.24-fold (*LanVIN3-3*, *P* = 0.0009). The vernalization effect differed between genotypes and photoperiods. Under LD it was significant for 83A:476 (repression of all three genes, up to 0.43-fold) and Palestyna (induction of *LanVIN3-2* and *LanVIN3-3*, up to 2.0-fold), whereas under SD it was significant for 83A:476 (changing from repression, up to 0.18-fold, to induction, up to 2.3-fold) and P27255 (induction, up to 3.6-fold). To summarize, all *LanVIN3* homologs revealed different transcriptomic responses to vernalization than the *LanFTc1* and *LanAGL8* genes.

Besides VIN3, VRN5 protein is also considered to participate in the vernalization-induced epigenetic silencing in *A. thaliana* (Greb et al., 2007). Therefore, the expression of the latter gene was also profiled to supplement the analysis. All three *L. angustifolius* copies, *LanVRN5-1*, *LanVRN5-2*, and *LanVRN5-3*, were analyzed using one universal primer pair (Supplementary Figure 6). *LanVRN5* clade revealed an expression profile relatively similar to *LanVIN3*, especially to *LanVIN3-2*. Like *LanVIN3* genes, *LanVRN5* in 83A:476 revealed an increase of expression during plant growth under LD and decrease under SD. All lines showed a significant decrease of expression during the light phase under SD and usually not a significant diurnal trend under LD. In both photoperiods without pre-sowing vernalization, 83A:476 revealed a significantly higher expression of *LanVRN5* than Palestyna and P27255. However, the vernalization responsiveness of *LanVRN5* considerably differed between photoperiods and genotypes. Under LD, only P27255 was responsive (showing repression), whereas under SD all lines responded but in different ways and with changing response during plant growth: namely from repression to induction in 83A:476, from induction to repression in Palestyna, and by continuously increasing induction in P27255. In vernalized plants under SD, *LanVRN5* expression was the highest in P27255.

## Discussion

### INDEL Polymorphism in the *LanFTc1* Promoter as a Legume Model for Functional Studies Aiming Vernalization Responsiveness

*FT* is a major flowering time integrator gene, gathering signals from several pathways that detect environmental conditions (Turck et al., 2008). First studies on the conservation of *A. thaliana* genes in legumes have revealed the presence of the well-preserved homologs of many flowering time regulatory genes, including some representatives of *FT* clade found in *Pisum sativum* L., *Medicago truncatula* L., and *Glicyne max* (L.), Merrill (Hecht et al., 2005). When the first gene-based linkage map of *L. angustifolius* was published, it highlighted a conserved collinear block shared between the fragment of *M. truncatula* chromosome 7 and the so-called linkage group LG01 (currently NLL-10), carrying major early flowering locus *Ku* (Nelson et al., 2006). It was later revealed that the syntenic region in *M. truncatula* contained several *FT* homologs (Young et al., 2011). The development of a bacterial artificial chromosome library for the *L. angustifolius* nuclear genome opened a possibility of gene cloning by DNA hybridization and the sequencing of selected clones (Kasprzak et al., 2006). Such an approach, combined with novel high-throughput sequencing techniques and gene expression profiling, resulted in the identification of a candidate gene for *Ku* and enabled the formulation of the hypothesis that a 1423 bp deletion in the promoter region of this gene is a causal mutation conferring early flowering phenotype (Nelson et al., 2017). Then, two other overlapping deletion variants, covering 1208 bp (*Pal*) and 5162 bp (*Jul*) were identified (Taylor et al., 2019). In the *A. thaliana*, a variation in promoter length was found to modulate the photoperiodic response of *FT*, and some important regulatory blocks contributing to this response were identified (Adrian et al., 2010; Liu et al., 2014). However, comparative mapping revealed a low sequence similarity between *LanFTc1* and *A. thaliana FT* promoters, except RE-alpha and CCAAT boxes which were found at expected positions and indicated that *FT* promoter length in *L. angustifolius* may be at least as big as in *A. thaliana* (up to 7 kb) (Ksia̧żkiewicz et al., 2016). All recognized *LanFTc1* deletions conferring *Ku*, *Pal*, and *Jul* alleles are located downstream of the pair of CCAAT boxes marking a putative beginning of the functional promoter (Ksia̧żkiewicz et al., 2016; Nelson et al., 2017; Taylor et al., 2019). Thus, *L. angustifolius Ku*, *Pal*, and *Jul* alleles have both distal and proximal regions preserved (Taylor et al., 2019). However, deletion sequences encompassed candidate binding sites for many transcription factors, including those already evidenced to be involved in *FT* regulation in model plants (Nelson et al., 2017; Taylor et al., 2019). In *A. thaliana*, similarly to *L. angustifolius*, the functional *FT* promoter indels also retained distal and proximal regions (Adrian et al., 2010; Liu et al., 2014). On the contrary, the study involving the *FT* promoter from cotton provided evidence that the proximal region might play an important role in this species (Sang et al., 2019). To our knowledge, a model revealed for *L. angustifolius* is the only known legume example of *FT* promoter indel variation. However, in many legumes, *FT* genes were found to be associated with flowering traits. In the economically most important legume crop worldwide, a soybean, *Glycine max*, mutations in *FT* genes are responsible for at least three loci conferring early/late flowering, namely *E9* (*GmFT2a*), *E10* (*GmFT4*), and *qDTF-J1* (*GmFT5a*) (Takeshima et al., 2016; Zhao et al., 2016; Samanfar et al., 2017). Moreover, natural variations of the *GmFT2b* sequence are associated with soybean adaption to high−latitude regions (Chen et al., 2020). In *M. truncatula*, vernalization responsiveness and early flowering are conferred by the *FTa1* gene, whereas photoperiod response by the *FTb* gene (Laurie et al., 2011; Putterill et al., 2013). In pea (*Pisum sativum*), the *FTa1* gene corresponds to the pea *GIGAS* locus, which is essential for flowering under LD and promoting flowering under SD (Hecht et al., 2011). In chickpea, a major quantitative trait locus (QTL) for the flowering time under SD conditions was mapped in the region containing a cluster of three *FT* genes (*FTa1*-*FTa2*-*FTc*), which collectively showed upregulated expression in domesticated germplasm (Ortega et al., 2019). In the sister lupin crop species, white lupin (*L. albus* L.), one of the four major QTLs conferring early flowering and partial vernalization independence was found associated with the *FTa1* gene (Rychel et al., 2019). *L. angustifolius* genome contains two *FTa* and two *FTc* genes, which putatively arose from single copies by lineage-specific duplication, whereas the whole *FTb* subclade is absent (Ksia̧żkiewicz et al., 2016). Indeed, *L. angustifolius* was recently used as a reference species in several phylogenetic studies addressing the influence of whole-genome and local duplications on the evolutionary fate of selected legume-specific and plant-wide gene clades (Przysiecka et al., 2015; Narożna et al., 2017; Szczepaniak et al., 2018; Czyż et al., 2020). The differences in the expression profiles for *FTa* and *FTc* genes, as established in the present study, provided novel evidence supporting the hypothesis on a functional divergence of particular duplicates.

### *Pal* Allele Carrying Intermediate Phenology and Light Vernalization Responsiveness Provides Worldwide Opportunities for *L. angustifolius* Breeding

In the present study, based on experiments performed in a greenhouse under natural LD (12–17 h photoperiod), *Ku* and *Jul* alleles were found to be associated with early flowering and vernalization independence, *Pal* with slightly delayed flowering and partial vernalization responsiveness, and *ku* with late flowering and high vernalization dependence. The same phenomenon was reported for the recent study performed in phytotron under natural 10–12 h photoperiod (intermediate between SD and LD) in Australia (Taylor et al., 2019). However, we observed relatively high differences in flowering time for early lines (*Ku*, *Pal*, and *Jul*) in a greenhouse between 2014 and 2015 repeats, accounting for about two weeks on average (earlier in 2014). These differences can be attributed to the variation in temperature which occurred during these experiments. During the first 70 days of the 2014 experiment, we observed 31 days with maximum temperature ≥25°C and 9 days with ≥30°C, whereas in ‘2015 these numbers were much lower, 3 and 0 days, respectively. The higher temperature could advance flowering because it is shown to strongly accelerate flowering in model plants as well as in many other plant species (Parmesan and Yohe, 2003; Thines et al., 2014). Moreover, the average photoperiod in 2014 was about 2 h longer than in 2015 due to differences in sowing terms. Indeed, our subsequent controlled-environment study revealed that even early *L. angustifolius* germplasm is responsive to LD conditions and accelerated transition between particular developmental phases by about 18–25 days compared to SD. The observed phenology of the *Pal* allele can be very beneficial for *L. angustifolius* cultivation in the era of changing climate, especially in Europe and Australia where the majority of worldwide lupin production occurs. Thus, the European land climate experienced rapid warming in recent decades, resulting in the mean year temperature surge to approximately 2°C above the 1910–1960 average (NOAA, 2020). Climate warming raised multiple challenging issues for grain legume breeders, including higher water deficits and severe drought periods, propagation of pests and diseases as well as de-regulation of temperature-based control of growth and development processes (Vadez et al., 2012; Scheelbeek et al., 2018; Lippmann et al., 2019). Affected regulatory pathways include, among others, the flowering time control (Nelson et al., 2010). The rapid flowering of domesticated germplasm may favor drought escape and adaptation for spring sowing in higher latitudes (Annicchiarico et al., 2010, 2018; Berger et al., 2017). However, the observed extension of the vegetation period has raised the demand for germplasm with intermediate phenology and cross-environment adaptation. Such research was recently initiated in *L. albus* in three European locations contrasting sowing time (autumn or spring) and climate type (Annicchiarico et al., 2019). Climatic variables were also addressed in an *L. angustifolius* genome-wide association study, providing some candidate polymorphisms that await further exploitation (Mousavi-Derazmahalleh et al., 2018a). *L. angustifolius Pal* allele confers flowering time and vernalization responsiveness phenotype intermediating between domesticated and wild lines. As this phenotype is consistent within the large range of photoperiod conditions (8, 10–12, and 16–17 h), it may be found applicable for all regions where lupins are currently cultivated.

### *LanFTc1*, *LanAGL8*, *LanCRLK1*, and *LanUGT85A2* Are Candidate Genes Involved in the Vernalization Responsiveness of *L. angustifolius*

Previous studies have highlighted the negative association of *LanFTc1* and *LanAGL8* gene expression with the number of days to flowering in *L. angustifolius*, mapping population and the positive direction of such association for *LanFD*, *LanCRLK1*, and *LanUGT85A2* genes (Nelson et al., 2017; Plewiński et al., 2019). The present study revealed that these genes differ in vernalization responsiveness between genotypes and photoperiods. *LanFTc1* and *LanAGL8* genes were found to be highly induced by vernalization in wild germplasm, whereas *LanUGT85A2* was found to be significantly suppressed (Table 4). *LanAGL8* protein sequence revealed the highest similarity to *A. thaliana FRUITFULL* (*FUL*, *AGAMOUS-LIKE 8*, AT5G60910) and *APETALA1* (*AP1*, *AGAMOUS-LIKE 7*, AT1G69120) genes. Both *AP1* and *FUL* play a role in floral meristem identity but have different functions. *AP1* controls the formation of sepals and petals whereas *FUL* is involved in cauline leaf and fruit development (Irish and Sussex, 1990; Gu et al., 1998). These genes revealed tissue-specific expression during generative organ development (Irish and Sussex, 1990; Mandel and Yanofsky, 1995; Klepikova et al., 2016). In the present study, high levels of *LanAGL8* expression were revealed in leaf tissue. Moreover, the expression profiles and vernalization responsiveness of *LanAGL8* and *LanFTc1* were very similar. Both genes revealed comparable circadian clock control, i.e., morning induction in *Pal* line under short days. Confronting these observations with the information on a 100% association between the *LanFTc1* genotype and flowering time phenotype in a large germplasm collection (Nelson et al., 2017; Taylor et al., 2019), the conclusion can be raised that *LanAGL8* acts putatively downstream of *LanFTc1* in *L. angustifolius*, in which *LanAGL8* may perform a similar function, like its homolog in cereals, an *AP1*-like gene called *VRN1*, which regulates the transition from vegetative to generative phase in response to vernalization and is expressed in many organs, including leaves (Trevaskis et al., 2003; Yan et al., 2003). Indeed, the wheat homolog of *FT*, (*VRN3*), activates expression of *VRN1* in leaves and shoot apical meristem, promoting flowering under inductive long days (Li et al., 2015). As a MADS box transcription factor, VRN1 binds to many targets in the genome and may regulate many genes, linking vernalization and photoperiod pathways (Deng et al., 2015). Moreover, the allelic diversity of *VRN1* copies provides wide plasticity of temperature-based responses in winter wheat (Dixon et al., 2019).

In this study, we also revealed differences in the vernalization responsiveness of a *LanFD* gene between early and late flowering germplasm. In wheat, FD−like, VRN3, and 14−3−3 proteins form together a florigen activation complex which can bind the *VRN1* promoter, therefore a variation in *FD* expression may modulate the effect of mobile florigen signal (Li et al., 2015). Results obtained in this study, are supported by a significant correlation between the *LanFD* gene expression profile and vernalization responsiveness in the *L. angustifolius* mapping population (Plewiński et al., 2019), which indicates that *LanFD* may contribute to *FT* regulatory function, especially in the wild, vernalization-responsive germplasm.

Our recent expression quantitative trait loci (eQTL) mapping study provided transcriptomic evidence for the contribution of several genes acting in C-repeat binding factor (CBF) cold responsiveness (*LanCRLK1)*, and in UDP-glycosyltransferases (*LanUGT85A2)* pathways in the vernalization response via *LanFTc1* in *L. angustifolius* (Plewiński et al., 2019). *LanCRLK1* is a homolog of *A. thaliana CALCIUM/CALMODULIN-REGULATED RECEPTOR-LIKE KINASE 1*, which is the first component in the cold responsiveness pathway (Yang et al., 2010). Downstream genes in this pathway, the C-repeat binding factors (*CBF*) and *INDUCER OF CBF EXPRESSION 1* (*ICE1*), provide regulatory links to *FLOWERING LOCUS C* (*FLC*) (Kim et al., 2004; Lee et al., 2015). The present study has highlighted the positive vernalization responsiveness of the *LanCRLK1* gene but only in wild germplasm under SD. In other genotype x environment combinations, the response was *quasi* thermoneutral. This finding is coherent with the general observation that CBF cold responsiveness pathway is downregulated and less effective under LD conditions than under SD (Lee and Thomashow, 2012). The expression profile of *LanCRLK1* did not provide convincing evidence on the contribution of this gene in the vernalization responsiveness of *L. angustifolius*. Nevertheless, the reduction of *LanCRLK1* expression in the evening, combined with the decreasing trend during development that was revealed in this study for vernalized *ku* line under LD, may explain the direction of the association between the *LanCRLK1* expression pattern and the vernalization responsiveness observed in the *L. angustifolius* mapping population e-QTL study, which was also performed under LD with partial vernalization (Plewiński et al., 2019). The question arises as to whether these differences in the *LanCRLK1* gene expression profile between the *Ku*/*Pal* and the *ku* lines, may have consequences in terms of cold acclimation and freezing tolerance of early flowering lines. A negative correlation between early phenology and cold acclimation could be a very undesirable trait hampering the autumn sowing of *L. angustifolius* in many regions of Southern Europe.

This study evidenced the negative response of *LanUGT85A2* to vernalization in all genotypes under both photoperiods. The genotypes explored in this study revealed different responses to photoperiod, and under SD, the *LanUGT85A2* expression was highest in the early flowering line, whereas under LD, it was in late flowering. Indeed, during the *L. angustifolius* mapping population e-QTL assay, which was performed under LD with mild vernalization, *LanUGT85A2* expression revealed a significant positive correlation with the late-flowering phenotype (Plewiński et al., 2019). *LanUGT85A2* is a representative of the UDP-glycosyltransferases protein family. In *A. thaliana*, a relatively close homolog of this gene, *UGT87A2*, promotes flowering in the vernalization and gibberellin pathways by repression of *FLC* (Wang B. et al., 2012). Similarly, ectopic over-expression in tobacco of a putative glycosyltransferase gene 1, *PtGT1*, derived from poplar (*Populus tomentosa* Carr.), resulted in an early flowering phenotype (Wang Y.-W. et al., 2012). Contrary, another *A. thaliana* homolog, *UGT84A2*, delays flowering by activation of the indole-3-butyric acid (IBA) pathway, leading to down-regulation of *AUXIN RESPONSE FACTOR 6* (*ARF6*) and *ARF8* genes, and, consequently *FT* (Zhang et al., 2017). Taking into consideration the direction of the association between *LanUGT85A2* expression and time to flowering, the latter mechanism seems to be more probable in *L. angustifolius* than those of *UGT87A2* and *PtGT1*.

This research highlighted the hypothetical involvement of *FLC*-related genes in *L. angustifolius* vernalization-dependent flowering time regulation. However, legume genomes, except for soybean, generally do not have *FLC* homologs (Hecht et al., 2005; Lyu et al., 2020). Nevertheless, other genes from the vernalization pathway, including some potential activators and repressors of *FLC*, are present (Hane et al., 2017). Interestingly, soybean has retained one *FLC* copy within its genome and does not require vernalization to initiate flowering. *FLC* in this species is involved in long-term low temperature-triggered late flowering by inhibiting *FT* gene expression (Lyu et al., 2020).

In *Arabidopsis*, vernalization-dependent silencing of the *FLC* gene is performed by *VIN3* and *VRN5* proteins which contribute to H3K27me3 and H3K9me2 enrichments during cold periods (Sung and Amasino, 2004; Greb et al., 2007; Kim and Sung, 2013). Despite their similar function in *A. thaliana*, *VIN3* and *VRN5* genes differ in expression profiles and vernalization responsiveness. Namely, *VIN3* expression in *A. thaliana* is very low and highly induced during vernalization, but soon after the end of the cold period, it decreases again to pre-vernalization level; whereas the *VRN5* gene is constitutively expressed at much higher levels than *VIN3* with additional high upregulation after the end of vernalization (Kim and Sung, 2013). In our study, *LanVIN3* genes revealed significantly higher relative expression than *LanVRN5*. Apart from this difference, expression profiles of *LanVRN5* and *LanVIN3* homologs were similar to each other. Moreover, this research provided lines of evidence for the high expression of three *LanVIN3* homologs in early flowering 83A:476 lines without vernalization, highlighting the opposite effects of vernalization for particular genotypes. The observed differences in vernalization responses and expression profiles of *LanVIN3* and *LanVRN5* genes did not match observed differences in phenotypes (time to flowering). This may indicate that *LanVIN3* and *La*n*VRN5* genes are not involved in the vernalization response in *L. angustifolius*.

## Data Availability Statement

All datasets presented in this study are included in the article/Supplementary Material.

## Author Contributions

SR-B designed experiments and performed plant phenotyping for time to flowering and vernalization responsiveness, plant genotyping with molecular markers, and gene expression profiling by quantitative PCR. PP performed RNA isolation and quantification. RG participated in the concept of the study and the interpretation of the results. BK contributed to data analysis and statistics. MK analyzed the data, interpreted the results, prepared the figures, and drafted the manuscript. All authors approved the final manuscript.

## Conflict of Interest

The authors declare that the research was conducted in the absence of any commercial or financial relationships that could be construed as a potential conflict of interest.

## Supplementary Material

Supplementary Table 1 Accession numbers and information on domestication status and country of origin of *L. angustifolius* lines used in the study.

Supplementary Table 2 Primers used in the study.

Supplementary Table 3 Air temperature (daily mean and maximum) and daily sunshine hours recorded by the local/nearest meteorological station (Poznań-Ławica, 5.1 km) and theoretical photoperiod hours calculated for this location during the 2014 experiment.

Supplementary Table 4 Air temperature (daily mean and maximum) and daily sunshine hours recorded by the local/nearest meteorological station (Poznań-Ławica, 5.1 km) and theoretical photoperiod hours calculated for this location during the 2015 experiment.

Supplementary Table 5 Sampling terms selected for gene expression profiling.

Supplementary Table 6 Leaf expression of selected flowering-related genes (reads per kilobase million, RPKM) derived from the transcriptome sequencing of *L. angustifolius* mapping population and correlation with vernalization responsiveness (*ku*).

Supplementary Table 7 R2 and PCR efficiency values obtained for standard curves.

Supplementary Table 8 Comparison of LanFTc1_INDEL2 marker scores and time to flowering in response to vernalization observed during ‘2014 experiment.

Supplementary Table 9 Comparison of LanFTc1_INDEL2 marker scores and time to flowering in response to vernalization observed during ‘2015 experiment.

Supplementary Figure 1 Gene expression profile of the *LanFTa2* gene in response to photoperiod and vernalization in three lines (83A:476, Palestyna, and P27255) carrying different *LanFTc1* alleles (*Ku*, *Pal*, and *ku*).

Supplementary Figure 2 Gene expression profile of the *LanFTc2* gene in response to photoperiod and vernalization in three lines (83A:476, Palestyna, and P27255) carrying different *LanFTc1* alleles (*Ku*, *Pal*, and *ku*).

Supplementary Figure 3 Gene expression profile of the *LanVIN3-1* gene in response to photoperiod and vernalization in three lines (83A:476, Palestyna, and P27255) carrying different *LanFTc1* alleles (*Ku*, *Pal*, and *ku*).

Supplementary Figure 4 Gene expression profile of the *LanVIN3-2* gene in response to photoperiod and vernalization in three lines (83A:476, Palestyna, and P27255) carrying different *LanFTc1* alleles (*Ku*, *Pal*, and *ku*).

Supplementary Figure 5 Gene expression profile of the *LanVIN3-3* gene in response to photoperiod and vernalization in three lines (83A:476, Palestyna, and P27255) carrying different *LanFTc1* alleles (*Ku*, *Pal*, and *ku*).

Supplementary Figure 6 Gene expression profile of *LanVRN5* genes in response to photoperiod and vernalization in three lines (83A:476, Palestyna, and P27255) carrying different *LanFTc1* alleles (*Ku*, *Pal*, and *ku*).

Supplementary Data Sheet 1 Gene expression data and calculations for *LanAGL8*, *LanCRLK1*, *LanFD*, *LanFTa1*, *LanFTa2*, *LanFTc1*, *LanFTc2*, and *LanUGT85A2* genes, obtained for two photoperiod variants (8- and 16-h) in response to vernalization in three lines (83A:476, Palestyna, and P27255) carrying different *LanFTc1* alleles (*Ku*, *Pal*, and *ku*).
