# Supplementary material for: Photoperiod and Vernalization Control of Flowering-Related Genes: A Case Study of the Narrow-Leafed Lupin (Lupinus angustifolius L.)
Source: Front Plant Sci. 2020 Oct 26;11:572135. doi: 10.3389/fpls.2020.572135 (PMC7663182; doi:10.3389/fpls.2020.572135)
Supplement: Supplementary Table 1 — Accession numbers and information on domestication status and country of origin of L. angustifolius lines used in the study. [file Table_1.DOCX]

**Photoperiod and vernalization control of flowering-related genes: a case study of the narrow-leafed lupin (*Lupinus angustifolius* L.)**

**Sandra Rychel-Bielska^1,2^, Piotr Plewiński^2^, Bartosz Kozak^1^, Renata Galek^1^, Michał Książkiewicz^2*^**

^1^ Department of Genetics, Plant Breeding and Seed Production, Wroclaw University of Environmental and Life Sciences, Wrocław, Poland

^2^ Department of Genomics, Institute of Plant Genetics, Polish Academy of Sciences, Poznań, Poland

Supplementary Material

[1 Supplementary Tables 2](#_Toc49525802)

[1.1 Supplementary Table S1. Accession numbers and information on domestication status and country of origin of *L. angustifolius* lines used in the study. 2](#_Toc49525803)

[1.2 Supplementary Table S2. Primers used in the study. 4](#_Toc49525804)

[1.3 Supplementary Table S3. Air temperature (daily mean and maximum) and daily sunshine hours recorded by the nearby localized meteorological station (Poznań-Ławica, 5.1 km) and theoretical photoperiod hours calculated for this location during ‘2014 experiment. 5](#_Toc49525805)

[1.4 Supplementary Table S4. Air temperature (daily mean and maximum) and daily sunshine hours recorded by the nearby localized meteorological station (Poznań-Ławica, 5.1 km) and theoretical photoperiod hours calculated for this location during ‘2015 experiment. 8](#_Toc49525806)

[1.5 Supplementary Table S5. Sampling terms selected for gene expression profiling. 11](#_Toc49525807)

[1.6 Supplementary Table S6. Leaf expression of *LFY*, *VIN3*, *AP1* and *VRN5* genes (reads per kilobase million, RPKM) derived from transcriptome sequencing of *L. angustifolius* mapping population. 12](#_Toc49525808)

[1.7 Supplementary Table S7. R2 and PCR efficiency values obtained for standard curves. 13](#_Toc49525809)

[1.8 Supplementary Table S8. Comparison of LanFTc1_INDEL2 marker scores and time to flowering in response to vernalization observed during ‘2014 experiment. 14](#_Toc49525810)

[1.9 Supplementary Table S9. Comparison of LanFTc1_INDEL2 marker scores and time to flowering in response to vernalization observed during ‘2015 experiment. 16](#_Toc49525811)

[2 Supplementary Figures 18](#_Toc49525812)

# Supplementary Tables

# 1.1 Supplementary Table S1. Accession numbers and information on domestication status and country of origin of *L. angustifolius* lines used in the study.

| **Accession** | **Name** | **Domestication** | **Country** |
| --- | --- | --- | --- |
| 96160 | Orlowskij Mutant | cultivar | Russia |
| 96194 | Borweta | cultivar | Germany |
| 96375 | W-179 | cross derivative or breeding line | Poland |
| 96150 | WTD-7031 | cross derivative or breeding line | Poland |
| 96182 | Biały Murzyn | mutant | Poland |
| 96131 | Stadoliszczienskij L-667 | cross derivative or breeding line | Russia |
| 96161 | Yorrel | cultivar | Australia |
| 96209 | Elf | cultivar | Poland |
| 96134 | Kupala | cross derivative or breeding line | Russia |
| 95705 | Cinded Rodrigo 1 | wild or primitive | Spain |
| 96129 | Stadoliszczienskij L-610 | cross derivative or breeding line | Russia |
| 96178 | Silena | cultivar | Belarus |
| 95796 | Mirela | cultivar | Poland |
| 95765 | St.Treb.-8 | cross derivative or breeding line | Germany |
| 96158 | Gelena | cultivar | Russia |
| 95932 | Population 25097 | cross derivative or breeding line | Russia |
| 96195 | Bolivio | cultivar | Germany |
| 96216 | Bora | cultivar | Germany |
| 96217 | Borlu | cultivar | Germany |
| 96148 | WTD-386 | cross derivative or breeding line | Poland |
| 96101 | Uniwhite | cultivar | Australia |
| 96162 | Gunguru | cultivar | Australia |
| 95931 | Population 25087 | cross derivative or breeding line | Russia |
| 95916 | BRGC-10271 | cross derivative or breeding line | Italy |
| 96179 | Determinant-4 | cross derivative or breeding line | Belarus |
| 96180 | Determinant-5 | cross derivative or breeding line | Belarus |
| 96164 | Helios (WTD-7035) | cross derivative or breeding line | Poland |
| 96155 | R-7009 x Chittick | cross derivative or breeding line | Poland |
| 96188 | Reduced Branching | cross derivative or breeding line | Australia |
| 96120 | Kazan | cultivar | Poland |
| 96132 | Stadoliszczienskij L-663 | cross derivative or breeding line | Russia |
| 95903 | Population Aqueb. Jordan Valley | wild or primitive | Israel |
| 95929 | WTD-793 (Ernani) | cultivar | Poland |
| 96184 | Sur | cultivar | Poland |
| 96173 | R 85L,482 | cross derivative or breeding line | Australia |
| 96192 | Bordako | cultivar | Germany |
| 96196 | W-89 | cross derivative or breeding line | Poland |
| 96116 | Illyarie | cultivar | Australia |
| 96117 | Yandee | cultivar | Australia |
| 96128 | Stadoliszczienskij L-569 | cross derivative or breeding line | Russia |
| 96143 | Danja | cultivar | Australia |
| 96145 | AN-2547 | cultivar | Spain |
| 96102 | Unicrop | cultivar | Australia |
| 95845 | Population 22873 | wild or primitive | Morocco |
| 95799 | Palestyna | wild or primitive | Palestine |
| 95826 | Brianskij 35 | cross derivative or breeding line | Russia |
| 95842 | Population 22661 | wild or primitive | Israel |
| 95944 | Population 22695 | wild or primitive | Spain |
| 95852 | Population 22708 | wild or primitive | Spain |
| 95963 | Jakkalsfontein | cultivar | Republic of South Africa |
| 95782 | Turkus x Swaloef Boerre | cross derivative or breeding line | Poland |
| 96105 | St.Treb.-1 | cross derivative or breeding line | Germany |
| 95722 | Badajoz 7 | wild or primitive | Spain |
| 95736 | Population B-540/79 | wild or primitive | Spain |
| 95706 | Vitigudino-1 | wild or primitive | Spain |
| 95942 | P.22745 | wild or primitive | Australia |
| 95961 | Semilla de conlor | wild or primitive | unknown |
| 95730 | Population B-519/79 | wild or primitive | Spain |
| 95701 | Hinojoso de Duero 1 | wild or primitive | Spain |
| 95703 | Hinojoso de Duero 3 | wild or primitive | Spain |
| 95711 | Badajoz 4 | wild or primitive | Spain |
| 95744 | Population B-551/79 | wild or primitive | Spain |
| 95737 | Population B-541/79 | wild or primitive | Spain |
| 95754 | Population B-575/79 | wild or primitive | Spain |
| 95948 | Population 1130 | wild or primitive | Algeria |
| 95726 | Near Salamanca-b | wild or primitive | Spain |
| 96372 | Porto | wild or primitive | Portugal |
| 95750 | Population B-555/79 | wild or primitive | Spain |
| 95759 | Population B-598/79 | wild or primitive | Spain |
| 95896 | R-1192 | wild or primitive | Spain |
| 95919 | BRGC-10275 | wild or primitive | Italy |
| 95734 | Population B-532/79 | wild or primitive | Spain |
| 95950 | Population B-1008 | wild or primitive | Italy |
| 95756 | Population B-585/79[DP] | wild or primitive | Spain |
| 95702 | Hinojoso de Duero 2 | wild or primitive | Spain |
| 95729 | Population B-518/79 | wild or primitive | Spain |
| 95735 | Population B-538/79 | wild or primitive | Spain |
| 95883 | Sieviernyj 2 | cultivar | Russia |
| 95947 | Chocho Buerro | wild or primitive | Spain |
| 95746 | Population B-552/79b | wild or primitive | Spain |
| 95704 | Ejeme Salamanca | wild or primitive | Spain |
| 95745 | Population B-552/79a | wild or primitive | Spain |
| 95889 | A-1069 | wild or primitive | Spain |
| 95716 | Hinojoso de Duero 7 | wild or primitive | Spain |
| 95915 | BRGC-10270 | wild or primitive | Italy |
| 95853 | Population 22759 | wild or primitive | Spain |
| 95753 | Population B-570/79 | wild or primitive | Spain |
| 95738 | Population B-542/79 | wild or primitive | Spain |
| 96185 | Sonet | cultivar | Poland |
| 96245 | Regent | cultivar | Poland |
| 96233 | 83A:476 | cross derivative or breeding line | Australia |
| 96234 | P27255 | wild or primitive | Morocco |

# 1.2 Supplementary Table S2. Primers used in the study.

| **Origin of primer** | **Gene name** | **Usage** | **Primer sequence** | **Product length** |
| --- | --- | --- | --- | --- |
| Taylor et al. 2019 | *LanFTc1* INDEL2 | Genotyping | TCATATGGCTGCAACCTGAA GCTTCCTTCCTTTGCCTTCT | ku 5969 bp Pal 4761 bp Ku 4546 bp Jul 757 bp |
| This study | *LanAGL8* | RT-PCR | ACAACAACTTGATTCTGCTCTCA TTTGCACTCTCTGTTGCTCCT | 179 bp |
| This study | *LanCRLK1* | RT-PCR | GTGGTTATTGGAGTTGTTTTAGCA GCCATTTCGTCCTGACTTCAC | 195 bp |
| This study | *LanFD* | RT-PCR | TCTTCTTCTTTTGCTGACTTGCTG TGGGGAAAGGAGAAACAAGCG | 191 bp |
| This study | *LanFTa1* | RT-PCR | GAAACCCTCTTGTTGTTGGACG ATCATCTCCACCAACACTCACC | 164 bp |
| This study | *LanFTa2* | RT-PCR | GGAACCCTCTAGTTGTAGGACG GTCATCGCCACCAATAGTCACT | 164 bp |
| Nelson et al. 2017 | *LanFTc1* | RT-PCR | ATCCCAGCTACCACAAGTGC ACGCCATTCTGGAGCAAAGA | 141 bp |
| Nelson et al. 2017 | *LanFTc2* | RT-PCR | TATTCCAGCTACCACAAGTG AAAACATTGCGATGAATCCC | 90 bp |
| Taylor et al. 2016 | *LanDExH7* | RT-PCR | TTGTACGAGGTCGGTGCTCT ACAAGCAACCAAATATTGCACCATA | 127 bp |
| Taylor et al. 2016 | *LanTUB6* | RT-PCR | TTTGCACCCCTTACCTCCC GCAGCACACATCATGTTTTTGG | 101 bp |
| This study | *LanUGT85A2* | RT-PCR | AGAGTTTGTGTTTTGGACTGCC AGTGTCTCTAATGAAGCTAGGAAGG | 196 bp |
| This study | *LanVIN3-1* | RT-PCR | GATGAGGGGTGTGGTGATCG  ATTGATGTTCTCTTGCTTGATATACATTCT | 245 bp |
| This study | *LanVIN3-2* | RT-PCR | GATGAAGGGTGTACTGATCGTGA  CACCATTGAAGTTCTCTTGCTTGATATG | 249 bp |
| This study | *LanVIN3-3* | RT-PCR | CATGATGGGTGTGCTGATCGT  GTTGACGTTCTCTTGCTTGATATACATT | 245 bp |
| This study | *LanVRN5* (bulk) | RT-PCR | TCCTTTTGAGTCTTCCAGGGATG  CTCCCATGTGATCTTGCTAAACC | 120 bp |

Taylor CM et al. (2019) INDEL variation in the regulatory region of the major flowering time gene *LanFTc1* is associated with vernalization response and flowering time in narrow-leafed lupin (*Lupinus angustifolius* L.) Plant, Cell Environ 42:174-187 doi:10.1111/pce.13320

Nelson MN et al. (2017) The loss of vernalization requirement in narrow-leafed lupin is associated with a deletion in the promoter and de-repressed expression of a *Flowering Locus T* (*FT*) homologue New Phytol 213:220-232 doi:10.1111/nph.14094

# 1.3 Supplementary Table S3. Air temperature (daily mean and maximum) and daily sunshine hours recorded by the nearby localized meteorological station (Poznań-Ławica, 5.1 km) and theoretical photoperiod hours calculated for this location during ‘2014 experiment.

| **Date** | **Day from sowing** | **Maximum temperature** | **Daily mean temperature** | **Sunshine hours** | **Photoperiod hours** |
| --- | --- | --- | --- | --- | --- |
| 2014-05-14 | 0 | 14.4 | 10.2 | 6.7 | 15.7 |
| 2014-05-15 | 1 | 16.2 | 11.2 | 13.4 | 15.7 |
| 2014-05-16 | 2 | 16.8 | 11.7 | 2.3 | 15.8 |
| 2014-05-17 | 3 | 16.3 | 12.4 | 0.0 | 15.8 |
| 2014-05-18 | 4 | 17.3 | 13.5 | 2.0 | 15.9 |
| 2014-05-19 | 5 | 21.3 | 15.5 | 11.2 | 15.9 |
| 2014-05-20 | 6 | 25.7 | 19.2 | 14.8 | 16.0 |
| 2014-05-21 | 7 | 27.1 | 20.9 | 15.3 | 16.0 |
| 2014-05-22 | 8 | 28.7 | 23.2 | 13.9 | 16.1 |
| 2014-05-23 | 9 | 30.0 | 24.1 | 14.1 | 16.1 |
| 2014-05-24 | 10 | 27.8 | 20.0 | 7.3 | 16.2 |
| 2014-05-25 | 11 | 24.5 | 19.4 | 13.4 | 16.2 |
| 2014-05-26 | 12 | 24.6 | 19.6 | 13.1 | 16.3 |
| 2014-05-27 | 13 | 25.4 | 18.6 | 10.0 | 16.3 |
| 2014-05-28 | 14 | 18.0 | 10.5 | 0.0 | 16.3 |
| 2014-05-29 | 15 | 10.9 | 9.2 | 0.6 | 16.4 |
| 2014-05-30 | 16 | 17.4 | 12.4 | 9.9 | 16.4 |
| 2014-05-31 | 17 | 21.3 | 15.0 | 14.8 | 16.4 |
| 2014-06-01 | 18 | 19.5 | 14.8 | 13.9 | 16.5 |
| 2014-06-02 | 19 | 17.7 | 12.7 | 1.8 | 16.5 |
| 2014-06-03 | 20 | 15.5 | 13.2 | 0.4 | 16.5 |
| 2014-06-04 | 21 | 18.3 | 14.4 | 3.8 | 16.6 |
| 2014-06-05 | 22 | 22.0 | 16.8 | 9.0 | 16.6 |
| 2014-06-06 | 23 | 22.8 | 18.1 | 11.9 | 16.6 |
| 2014-06-07 | 24 | 26.7 | 20.6 | 15.9 | 16.6 |
| 2014-06-08 | 25 | 31.0 | 24.8 | 14.7 | 16.7 |
| 2014-06-09 | 26 | 30.5 | 24.4 | 13.1 | 16.7 |
| 2014-06-10 | 27 | 26.8 | 21.5 | 7.3 | 16.7 |
| 2014-06-11 | 28 | 28.3 | 22.9 | 11.0 | 16.7 |
| 2014-06-12 | 29 | 26.2 | 19.1 | 14.1 | 16.7 |
| 2014-06-13 | 30 | 19.4 | 14.3 | 2.7 | 16.8 |
| 2014-06-14 | 31 | 19.0 | 14.0 | 9.2 | 16.8 |
| 2014-06-15 | 32 | 20.4 | 14.7 | 10.7 | 16.8 |
| 2014-06-16 | 33 | 21.4 | 15.4 | 14.4 | 16.8 |
| 2014-06-17 | 34 | 22.1 | 16.7 | 12.6 | 16.8 |
| 2014-06-18 | 35 | 23.2 | 17.2 | 15.9 | 16.8 |
| 2014-06-19 | 36 | 21.7 | 14.8 | 3.4 | 16.8 |
| 2014-06-20 | 37 | 16.4 | 13.9 | 3.0 | 16.8 |
| 2014-06-21 | 38 | 17.8 | 13.2 | 2.9 | 16.8 |
| 2014-06-22 | 39 | 18.6 | 13.8 | 7.4 | 16.8 |
| 2014-06-23 | 40 | 17.0 | 12.9 | 8.8 | 16.8 |
| 2014-06-24 | 41 | 18.9 | 13.6 | 6.7 | 16.8 |
| 2014-06-25 | 42 | 20.2 | 14.8 | 5.9 | 16.8 |
| 2014-06-26 | 43 | 21.1 | 15.4 | 14.2 | 16.8 |
| 2014-06-27 | 44 | 23.4 | 17.7 | 13.7 | 16.8 |
| 2014-06-28 | 45 | 25.6 | 19.7 | 5.6 | 16.8 |
| 2014-06-29 | 46 | 24.7 | 19.0 | 1.9 | 16.8 |
| 2014-06-30 | 47 | 19.3 | 16.0 | 1.2 | 16.7 |
| 2014-07-01 | 48 | 22.2 | 16.8 | 9.3 | 16.7 |
| 2014-07-02 | 49 | 22.6 | 17.2 | 14.1 | 16.7 |
| 2014-07-03 | 50 | 24.0 | 18.0 | 14.1 | 16.7 |
| 2014-07-04 | 51 | 30.5 | 23.3 | 15.2 | 16.7 |
| 2014-07-05 | 52 | 29.6 | 23.3 | 7.7 | 16.6 |
| 2014-07-06 | 53 | 30.2 | 24.8 | 12.9 | 16.6 |
| 2014-07-07 | 54 | 33.2 | 25.5 | 13.0 | 16.6 |
| 2014-07-08 | 55 | 32.2 | 24.4 | 8.8 | 16.6 |
| 2014-07-09 | 56 | 28.7 | 22.1 | 8.6 | 16.5 |
| 2014-07-10 | 57 | 25.4 | 20.5 | 8.2 | 16.5 |
| 2014-07-11 | 58 | 25.7 | 20.7 | 14.3 | 16.5 |
| 2014-07-12 | 59 | 22.7 | 17.8 | 15.4 | 16.4 |
| 2014-07-13 | 60 | 24.3 | 18.1 | 9.5 | 16.4 |
| 2014-07-14 | 61 | 26.2 | 21.0 | 7.9 | 16.4 |
| 2014-07-15 | 62 | 26.8 | 21.6 | 12.8 | 16.3 |
| 2014-07-16 | 63 | 27.7 | 22.4 | 12.5 | 16.3 |
| 2014-07-17 | 64 | 28.9 | 22.8 | 8.7 | 16.3 |
| 2014-07-18 | 65 | 30.2 | 24.6 | 14.4 | 16.2 |
| 2014-07-19 | 66 | 31.5 | 25.5 | 12.7 | 16.2 |
| 2014-07-20 | 67 | 34.3 | 26.3 | 11.6 | 16.1 |
| 2014-07-21 | 68 | 29.5 | 24.2 | 12.0 | 16.1 |
| 2014-07-22 | 69 | 29.5 | 23.6 | 13.7 | 16.0 |
| 2014-07-23 | 70 | 28.6 | 21.7 | 11.1 | 16.0 |
| 2014-07-24 | 71 | 21.8 | 17.7 | 0.0 | 15.9 |
| 2014-07-25 | 72 | 22.1 | 19.5 | 0.5 | 15.9 |
| 2014-07-26 | 73 | 28.6 | 22.9 | 12.6 | 15.8 |
| 2014-07-27 | 74 | 31.3 | 25.2 | 11.5 | 15.8 |
| 2014-07-28 | 75 | 32.1 | 24.7 | 11.8 | 15.7 |
| 2014-07-29 | 76 | 29.9 | 23.9 | 13.1 | 15.7 |
| 2014-07-30 | 77 | 30.2 | 23.3 | 8.7 | 15.6 |
| 2014-07-31 | 78 | 23.5 | 20.7 | 0.4 | 15.6 |
| 2014-08-01 | 79 | 25.4 | 21.2 | 10.5 | 15.5 |
| 2014-08-02 | 80 | 30.6 | 23.8 | 8.7 | 15.5 |
| 2014-08-03 | 81 | 32.8 | 23.6 | 9.8 | 15.4 |
| 2014-08-04 | 82 | 25.4 | 20.9 | 3.6 | 15.4 |
| 2014-08-05 | 83 | 23.8 | 19.4 | 4.7 | 15.3 |
| 2014-08-06 | 84 | 25.2 | 19.9 | 12.7 | 15.3 |
| 2014-08-07 | 85 | 27.0 | 21.9 | 13.6 | 15.2 |
| 2014-08-08 | 86 | 26.3 | 21.7 | 10.7 | 15.1 |
| 2014-08-09 | 87 | 31.1 | 23.5 | 11.8 | 15.1 |
| 2014-08-10 | 88 | 29.4 | 21.9 | 10.8 | 15.0 |
| 2014-08-11 | 89 | 22.9 | 20.6 | 0.5 | 15.0 |
| 2014-08-12 | 90 | 23.3 | 18.2 | 12.3 | 14.9 |
| 2014-08-13 | 91 | 24.3 | 19.1 | 6.0 | 14.8 |
| 2014-08-14 | 92 | 22.6 | 18.4 | 10.3 | 14.8 |
| 2014-08-15 | 93 | 22.5 | 17.2 | 11.8 | 14.7 |
| 2014-08-16 | 94 | 19.7 | 15.7 | 7.1 | 14.7 |
| 2014-08-17 | 95 | 20.8 | 16.5 | 4.8 | 14.6 |
| 2014-08-18 | 96 | 21.2 | 17.8 | 0.0 | 14.5 |
| 2014-08-19 | 97 | 21.4 | 16.1 | 13.4 | 14.5 |
| 2014-08-20 | 98 | 18.9 | 14.8 | 12.6 | 14.4 |
| 2014-08-21 | 99 | 19.9 | 14.0 | 9.5 | 14.3 |
| 2014-08-22 | 100 | 22.7 | 15.6 | 9.1 | 14.3 |
| 2014-08-23 | 101 | 19.9 | 15.9 | 3.8 | 14.2 |
| 2014-08-24 | 102 | 18.6 | 14.4 | 3.2 | 14.2 |
| 2014-08-25 | 103 | 19.0 | 13.5 | 10.9 | 14.1 |
| 2014-08-26 | 104 | 16.4 | 12.5 | 4.3 | 14.0 |
| 2014-08-27 | 105 | 21.2 | 13.2 | 7.7 | 14.0 |
| 2014-08-28 | 106 | 22.8 | 16.0 | 13.1 | 13.9 |
| 2014-08-29 | 107 | 23.8 | 16.6 | 7.5 | 13.8 |
| 2014-08-30 | 108 | 22.1 | 17.7 | 3.0 | 13.8 |
| 2014-08-31 | 109 | 19.8 | 16.3 | 0.2 | 13.7 |
| 2014-09-01 | 110 | 17.2 | 15.2 | 0.0 | 13.6 |
| 2014-09-02 | 111 | 14.4 | 13.5 | 0.0 | 13.6 |
| 2014-09-03 | 112 | 21.0 | 16.1 | 12.0 | 13.5 |
| 2014-09-04 | 113 | 22.9 | 16.9 | 11.5 | 13.4 |
| 2014-09-05 | 114 | 23.6 | 17.5 | 11.7 | 13.4 |
| 2014-09-06 | 115 | 24.3 | 17.0 | 11.5 | 13.3 |
| 2014-09-07 | 116 | 25.6 | 19.1 | 10.3 | 13.2 |
| 2014-09-08 | 117 | 22.5 | 18.3 | 6.3 | 13.2 |
| 2014-09-09 | 118 | 20.6 | 16.4 | 6.5 | 13.1 |
| 2014-09-10 | 119 | 17.4 | 14.2 | 1.3 | 13.0 |
| 2014-09-11 | 120 | 19.0 | 15.5 | 2.1 | 13.0 |

# 1.4 Supplementary Table S4. Air temperature (daily mean and maximum) and daily sunshine hours recorded by the nearby localized meteorological station (Poznań-Ławica, 5.1 km) and theoretical photoperiod hours calculated for this location during ‘2015 experiment.

| **Date** | **Day from sowing** | **Maximum temperature** | **Daily mean temperature** | **Sunshine hours** | **Photoperiod hours** |
| --- | --- | --- | --- | --- | --- |
| 2015-03-25 | 0 | 17.4 | 9.3 | 8.0 | 12.5 |
| 2015-03-26 | 1 | 13.7 | 10.2 | 0.0 | 12.6 |
| 2015-03-27 | 2 | 9.0 | 7.1 | 0.3 | 12.6 |
| 2015-03-28 | 3 | 9.3 | 6.2 | 1.8 | 12.7 |
| 2015-03-29 | 4 | 10.8 | 6.4 | 0.0 | 12.8 |
| 2015-03-30 | 5 | 9.2 | 7.0 | 3.3 | 12.8 |
| 2015-03-31 | 6 | 8.2 | 4.3 | 0.0 | 12.9 |
| 2015-04-01 | 7 | 7.8 | 3.2 | 3.0 | 13.0 |
| 2015-04-02 | 8 | 4.5 | 1.6 | 2.6 | 13.0 |
| 2015-04-03 | 9 | 8.3 | 3.6 | 5.6 | 13.1 |
| 2015-04-04 | 10 | 9.5 | 2.2 | 8.5 | 13.2 |
| 2015-04-05 | 11 | 8.1 | 2.5 | 4.6 | 13.2 |
| 2015-04-06 | 12 | 7.2 | 3.0 | 2.0 | 13.3 |
| 2015-04-07 | 13 | 10.7 | 4.5 | 6.3 | 13.4 |
| 2015-04-08 | 14 | 11.5 | 8.1 | 0.5 | 13.4 |
| 2015-04-09 | 15 | 13.1 | 7.1 | 10.9 | 13.5 |
| 2015-04-10 | 16 | 18.7 | 10.5 | 11.9 | 13.6 |
| 2015-04-11 | 17 | 21.7 | 14.0 | 9.1 | 13.6 |
| 2015-04-12 | 18 | 18.9 | 10.7 | 8.5 | 13.7 |
| 2015-04-13 | 19 | 11.5 | 8.6 | 5.4 | 13.8 |
| 2015-04-14 | 20 | 11.4 | 7.3 | 2.9 | 13.8 |
| 2015-04-15 | 21 | 20.4 | 14.5 | 5.7 | 13.9 |
| 2015-04-16 | 22 | 17.6 | 10.7 | 12.0 | 14.0 |
| 2015-04-17 | 23 | 11.9 | 7.0 | 11.6 | 14.0 |
| 2015-04-18 | 24 | 9.6 | 4.8 | 6.8 | 14.1 |
| 2015-04-19 | 25 | 14.0 | 7.6 | 10.7 | 14.2 |
| 2015-04-20 | 26 | 16.8 | 9.6 | 13.2 | 14.2 |
| 2015-04-21 | 27 | 20.5 | 11.2 | 13.9 | 14.3 |
| 2015-04-22 | 28 | 16.0 | 9.4 | 13.7 | 14.4 |
| 2015-04-23 | 29 | 19.6 | 12.2 | 13.2 | 14.4 |
| 2015-04-24 | 30 | 20.1 | 13.4 | 13.0 | 14.5 |
| 2015-04-25 | 31 | 22.5 | 15.8 | 9.2 | 14.6 |
| 2015-04-26 | 32 | 20.4 | 16.5 | 8.1 | 14.6 |
| 2015-04-27 | 33 | 24.5 | 16.5 | 9.6 | 14.7 |
| 2015-04-28 | 34 | 18.2 | 7.5 | 0.0 | 14.7 |
| 2015-04-29 | 35 | 12.8 | 7.3 | 9.2 | 14.8 |
| 2015-04-30 | 36 | 15.5 | 10.5 | 7.5 | 14.9 |
| 2015-05-01 | 37 | 14.3 | 9.5 | 7.4 | 14.9 |
| 2015-05-02 | 38 | 14.6 | 8.8 | 12.0 | 15.0 |
| 2015-05-03 | 39 | 18.3 | 11.6 | 13.1 | 15.0 |
| 2015-05-04 | 40 | 21.0 | 14.9 | 4.5 | 15.1 |
| 2015-05-05 | 41 | 25.5 | 19.2 | 3.4 | 15.2 |
| 2015-05-06 | 42 | 23.1 | 16.5 | 4.1 | 15.2 |
| 2015-05-07 | 43 | 19.6 | 14.1 | 12.5 | 15.3 |
| 2015-05-08 | 44 | 18.7 | 12.6 | 9.0 | 15.3 |
| 2015-05-09 | 45 | 21.5 | 14.7 | 5.9 | 15.4 |
| 2015-05-10 | 46 | 15.8 | 11.4 | 4.4 | 15.5 |
| 2015-05-11 | 47 | 18.4 | 12.4 | 11.6 | 15.5 |
| 2015-05-12 | 48 | 24.3 | 17.3 | 6.9 | 15.6 |
| 2015-05-13 | 49 | 21.6 | 12.8 | 11.9 | 15.6 |
| 2015-05-14 | 50 | 15.8 | 10.6 | 7.0 | 15.7 |
| 2015-05-15 | 51 | 17.7 | 11.6 | 13.9 | 15.7 |
| 2015-05-16 | 52 | 18.0 | 11.8 | 7.0 | 15.8 |
| 2015-05-17 | 53 | 14.5 | 11.3 | 7.6 | 15.8 |
| 2015-05-18 | 54 | 19.3 | 13.7 | 9.6 | 15.9 |
| 2015-05-19 | 55 | 22.9 | 17.6 | 7.7 | 15.9 |
| 2015-05-20 | 56 | 19.4 | 12.9 | 0.0 | 16.0 |
| 2015-05-21 | 57 | 17.1 | 11.3 | 2.5 | 16.0 |
| 2015-05-22 | 58 | 19.2 | 13.2 | 13.0 | 16.1 |
| 2015-05-23 | 59 | 20.6 | 14.7 | 4.2 | 16.1 |
| 2015-05-24 | 60 | 20.9 | 14.8 | 11.5 | 16.2 |
| 2015-05-25 | 61 | 23.2 | 16.3 | 10.6 | 16.2 |
| 2015-05-26 | 62 | 19.4 | 14.1 | 7.5 | 16.2 |
| 2015-05-27 | 63 | 14.6 | 10.8 | 5.1 | 16.3 |
| 2015-05-28 | 64 | 19.8 | 14.3 | 13.2 | 16.3 |
| 2015-05-29 | 65 | 20.4 | 15.3 | 11.6 | 16.4 |
| 2015-05-30 | 66 | 19.1 | 14.6 | 10.6 | 16.4 |
| 2015-05-31 | 67 | 20.2 | 14.3 | 11.4 | 16.4 |
| 2015-06-01 | 68 | 23.3 | 17.3 | 5.4 | 16.5 |
| 2015-06-02 | 69 | 25.1 | 18.4 | 11.5 | 16.5 |
| 2015-06-03 | 70 | 29.1 | 21.5 | 13.4 | 16.5 |
| 2015-06-04 | 71 | 21.9 | 16.0 | 15.9 | 16.6 |
| 2015-06-05 | 72 | 26.3 | 19.2 | 16.1 | 16.6 |
| 2015-06-06 | 73 | 31.9 | 24.1 | 15.2 | 16.6 |
| 2015-06-07 | 74 | 30.1 | 14.4 | 1.2 | 16.6 |
| 2015-06-08 | 75 | 20.9 | 15.3 | 7.3 | 16.7 |
| 2015-06-09 | 76 | 15.7 | 11.6 | 1.9 | 16.7 |
| 2015-06-10 | 77 | 21.2 | 15.0 | 13.6 | 16.7 |
| 2015-06-11 | 78 | 22.8 | 16.6 | 16.0 | 16.7 |
| 2015-06-12 | 79 | 24.2 | 17.3 | 6.0 | 16.7 |
| 2015-06-13 | 80 | 27.5 | 20.2 | 9.8 | 16.8 |
| 2015-06-14 | 81 | 26.1 | 21.2 | 13.9 | 16.8 |
| 2015-06-15 | 82 | 22.6 | 16.0 | 13.4 | 16.8 |
| 2015-06-16 | 83 | 17.3 | 13.0 | 4.1 | 16.8 |
| 2015-06-17 | 84 | 20.5 | 15.3 | 14.4 | 16.8 |
| 2015-06-18 | 85 | 19.4 | 15.1 | 1.9 | 16.8 |
| 2015-06-19 | 86 | 18.0 | 12.2 | 5.2 | 16.8 |
| 2015-06-20 | 87 | 17.9 | 13.0 | 6.0 | 16.8 |
| 2015-06-21 | 88 | 16.9 | 13.1 | 3.9 | 16.8 |
| 2015-06-22 | 89 | 18.3 | 13.7 | 3.4 | 16.8 |
| 2015-06-23 | 90 | 15.7 | 12.2 | 1.5 | 16.8 |
| 2015-06-24 | 91 | 15.8 | 12.6 | 2.3 | 16.8 |
| 2015-06-25 | 92 | 20.3 | 15.4 | 5.9 | 16.8 |
| 2015-06-26 | 93 | 22.4 | 16.8 | 2.7 | 16.8 |
| 2015-06-27 | 94 | 22.9 | 17.7 | 3.9 | 16.8 |
| 2015-06-28 | 95 | 17.5 | 15.7 | 0.1 | 16.8 |
| 2015-06-29 | 96 | 22.0 | 16.4 | 5.2 | 16.8 |
| 2015-06-30 | 97 | 24.9 | 19.5 | 11.6 | 16.7 |
| 2015-07-01 | 98 | 24.9 | 19.2 | 16.1 | 16.7 |
| 2015-07-02 | 99 | 27.8 | 21.1 | 16.0 | 16.7 |
| 2015-07-03 | 100 | 29.9 | 23.4 | 16.0 | 16.7 |
| 2015-07-04 | 101 | 33.0 | 25.7 | 15.7 | 16.7 |
| 2015-07-05 | 102 | 33.9 | 27.1 | 7.2 | 16.6 |
| 2015-07-06 | 103 | 30.5 | 23.0 | 8.5 | 16.6 |
| 2015-07-07 | 104 | 31.4 | 23.6 | 15.7 | 16.6 |
| 2015-07-08 | 105 | 30.2 | 20.2 | 8.0 | 16.6 |
| 2015-07-09 | 106 | 20.0 | 15.7 | 8.4 | 16.5 |
| 2015-07-10 | 107 | 16.7 | 13.4 | 4.4 | 16.5 |
| 2015-07-11 | 108 | 22.3 | 16.0 | 14.3 | 16.5 |
| 2015-07-12 | 109 | 24.7 | 17.9 | 6.3 | 16.4 |
| 2015-07-13 | 110 | 20.7 | 16.9 | 2.2 | 16.4 |
| 2015-07-14 | 111 | 21.0 | 17.3 | 5.2 | 16.4 |
| 2015-07-15 | 112 | 23.2 | 18.6 | 10.8 | 16.3 |
| 2015-07-16 | 113 | 25.4 | 19.0 | 8.8 | 16.3 |
| 2015-07-17 | 114 | 28.5 | 22.7 | 11.5 | 16.3 |
| 2015-07-18 | 115 | 32.5 | 25.8 | 12.8 | 16.2 |
| 2015-07-19 | 116 | 29.7 | 21.6 | 9.1 | 16.2 |
| 2015-07-20 | 117 | 23.4 | 18.8 | 14.3 | 16.1 |
| 2015-07-21 | 118 | 28.7 | 20.3 | 6.9 | 16.1 |
| 2015-07-22 | 119 | 29.8 | 23.5 | 12.8 | 16.0 |
| 2015-07-23 | 120 | 27.2 | 20.9 | 8.6 | 16.0 |

# 1.5 Supplementary Table S5. Sampling terms selected for gene expression profiling.

| **Line** | **Term** | **Days from sowing** | **Days from flowering** | **Photoperiod** | **Vernalization** | **Flowering day** |
| --- | --- | --- | --- | --- | --- | --- |
| Palestyna | T1 | 50 | -21 | 8h | No | 71 |
| Palestyna | T2 | 58 | -13 | 8h | No | 71 |
| Palestyna | T3 | 65 | -7 | 8h | No | 71 |
| Palestyna | T4 | 72 | 1 | 8h | No | 71 |
| Palestyna | T1 | 40 | -16 | 8h | Yes | 56 |
| Palestyna | T2 | 44 | -12 | 8h | Yes | 56 |
| Palestyna | T3 | 50 | -6 | 8h | Yes | 56 |
| Palestyna | T4 | 58 | 2 | 8h | Yes | 56 |
| 83A:476 | T1 | 40 | -17 | 8h | No | 57 |
| 83A:476 | T2 | 44 | -13 | 8h | No | 57 |
| 83A:476 | T3 | 50 | -7 | 8h | No | 57 |
| 83A:476 | T4 | 58 | 1 | 8h | No | 57 |
| 83A:476 | T1 | 35 | -17 | 8h | Yes | 52 |
| 83A:476 | T2 | 40 | -12 | 8h | Yes | 52 |
| 83A:476 | T3 | 44 | -8 | 8h | Yes | 52 |
| 83A:476 | T4 | 54 | 2 | 8h | Yes | 52 |
| P27255 | T1 | 72 | - | 8h | No | - |
| P27255 | T2 | 79 | - | 8h | No | - |
| P27255 | T3 | 86 | - | 8h | No | - |
| P27255 | T4 | 93 | - | 8h | No | - |
| P27255 | T1 | 72 | - | 8h | Yes | - |
| P27255 | T2 | 79 | - | 8h | Yes | - |
| P27255 | T3 | 86 | [-](file:///C:\) | 8h | Yes | - |
| P27255 | T4 | 93 | - | 8h | Yes | - |
| Palestyna | T1 | 35 | -13 | 16h | No | 48 |
| Palestyna | T2 | 44 | -4 | 16h | No | 48 |
| Palestyna | T3 | 48 | 0 | 16h | No | 48 |
| Palestyna | T1 | 29 | -15 | 16h | Yes | 44 |
| Palestyna | T2 | 39 | -5 | 16h | Yes | 44 |
| Palestyna | T3 | 44 | 0 | 16h | Yes | 44 |
| 83A:476 | T1 | 25 | -14 | 16h | No | 39 |
| 83A:476 | T2 | 39 | 0 | 16h | No | 39 |
| 83A:476 | T1 | 21 | -14 | 16h | Yes | 35 |
| 83A:476 | T2 | 35 | 0 | 16h | Yes | 35 |
| P27255 | T1 | 42 | - | 16h | No | - |
| P27255 | T2 | 52 | - | 16h | No | - |
| P27255 | T3 | 59 | - | 16h | No | - |
| P27255 | T1 | 42 | -17 | 16h | Yes | 59 |
| P27255 | T2 | 52 | -7 | 16h | Yes | 59 |
| P27255 | T3 | 59 | 0 | 16h | Yes | 59 |

# 1.6 Supplementary Table S6. Leaf expression of selected flowering-related genes (reads per kilobase million, RPKM) derived from transcriptome sequencing of *L. angustifolius* mapping population and correlation with vernalization responsiveness (*ku*).

| **Name** | **Gene** | **NCBI protein** | **Mean RPKM** | **Standard deviation** | **Correlation with *ku*** | **FDR-corrected significance** |
| --- | --- | --- | --- | --- | --- | --- |
| *LanLFY-1* | Lup006312 | OIW18228.1 | 0.057 | 0.155 | -0.090 | - |
| *LanLFY-2* | Lup012189 | OIV98153.1 | 0.040 | 0.117 | 0.090 | - |
| *LanLFY-3* | Lup027481 | OIW00230.1 | 0.035 | 0.127 | 0.090 | - |
| *LanVIN3-1* | Lup009440 | OIW19420.1 | 43.328 | 16.004 | -0.150 | - |
| *LanVIN3-2* | Lup013437 | OIW18685.1 | 17.330 | 3.754 | 0.090 | - |
| *LanVIN3-3* | Lup026125 | OIV99787.1 | 14.544 | 4.943 | -0.340 | - |
| *LanAP1-1* | Lup021855 | OIW02202.1 | 0.052 | 0.135 | -0.050 | - |
| *LanAP1-2* | Lup024348 | OIW11654.1 | 0.176 | 0.338 | -0.280 | - |
| *LanAP1-3* | Lup006876 | OIW16836.1 | 0.057 | 0.165 | -0.030 | - |
| *LanVRN5-1* | Lup009144 | OIW18950.1 | 0.381 | 0.427 | 0.110 | - |
| *LanVRN5-2* | Lup018692 | OIV93476.1 | 1.212 | 0.747 | 0.110 | - |
| *LanVRN5-3* | Lup032778 | OIW10038.1 | 0.087 | 0.202 | -0.070 | - |
| *LanFTa1* | Lup021189 | OIV90057.1 | 0.769 | 1.099 | -0.370 | - |
| *LanFTc1* | Lup015264 | OIW03334.1 | 4.845 | 5.448 | -0.510 | + |
| *LanFTc2* | Lup005674 | OIV93971.1 | 0.070 | 0.156 | -0.150 | - |
| *LanUGT85A2* | Lup002110 | OIW20134.1 | 11.006 | 6.688 | 0.520 | + |
| *LanCRLK1* | Lup011808 | OIW03171.1 | 14.230 | 12.631 | 0.850 | + |
| *LanAGL8* | Lup018485 | OIW19675.1 | 3.818 | 3.542 | -0.560 | + |
| *LanFD* | Lup018024 | OIV92673.1 | 1.352 | 0.996 | 0.530 | + |

Data extracted from Supplementary Tables S6 and S7 (Plewiński et al. 2019).

Plewiński P, Książkiewicz M, Rychel-Bielska S, Rudy E, Wolko B (2019) Candidate domestication-related genes revealed by expression quantitative trait loci mapping of narrow-leafed lupin (*Lupinus angustifolius* L.) Int J Mol Sci 20:5670 doi:10.3390/ijms20225670

# 1.7 Supplementary Table S7. R2 and PCR efficiency values obtained for standard curves.

| **Gene** | **PCR efficiency** | **R-Squared** |
| --- | --- | --- |
| *LanAGL8* | 94.5% | 0.999 |
| *LanCRLK1* | 77.0% | 0.998 |
| *LanFD* | 93.4% | 1.000 |
| *LanFTa1* | 87.2% | 0.998 |
| *LanFTa2* | 94.6% | 0.999 |
| *LanFTc1* | 84.0% | 0.998 |
| *LanFTc2* | 86.1% | 0.999 |
| *LanDExH7* | 97.5% | 0.999 |
| *LanTUB6* | 90.5% | 0.999 |
| *LanUGT85A2* | 95.5% | 1.000 |
| *LanVIN3-1* | 88.7% | 0.999 |
| *LanVIN3-2* | 96.5% | 0.997 |
| *LanVIN3-3* | 96.5% | 0.999 |
| *LanVRN5* (bulk) | 95.0% | 0.999 |

# 1.8 Supplementary Table S8. Comparison of LanFTc1_INDEL2 marker scores and time to flowering in response to vernalization observed during ‘2014 experiment.

| **Line** | **LanFTc1 INDEL2 marker** | **Days to flowering of non-vernalized plants '2014 (Mean)** | **Days to flowering of non-vernalized plants '2014 (Standard deviation)** | **Days to flowering of vernalized plants '2014 (Mean)** | **Days to flowering of vernalized plants '2014 (Standard deviation)** | **Change in days to flowering after vernalization '2014 (Mean)** |
| --- | --- | --- | --- | --- | --- | --- |
| 95896 | A | 116.25 | 3.03 | 49.86 | 4.73 | -66.39 |
| 96372 | A | 113.80 | 5.23 | 55.57 | 9.29 | -58.23 |
| 95919 | A | 113.75 | 1.09 | 52.14 | 3.48 | -61.61 |
| 95948 | A | 113.25 | 4.32 | 50.57 | 8.73 | -62.68 |
| 95705 | A | 111.40 | 5.04 | 51.86 | 1.25 | -59.54 |
| 95754 | A | 110.20 | 5.08 | 56.14 | 10.11 | -54.06 |
| 95703 | A | 109.80 | 7.68 | 62.40 | 7.61 | -47.40 |
| 95702 | A | 109.00 | 4.97 | 46.14 | 2.85 | -62.86 |
| 95852 | A | 107.00 | 3.41 | 43.29 | 1.75 | -63.71 |
| 95734 | A | 106.33 | 4.19 | 52.43 | 15.62 | -53.90 |
| 96234 | A | 105.67 | 5.56 | 50.14 | 4.55 | -55.52 |
| 95704 | A | 103.00 | 1.79 | 45.86 | 2.23 | -57.14 |
| 95711 | A | 102.00 | 8.17 | 47.14 | 3.04 | -54.86 |
| 95947 | A | 100.20 | 9.11 | 43.14 | 3.00 | -57.06 |
| 95701 | A | 100.20 | 9.11 | 56.71 | 9.38 | -43.49 |
| 95716 | A | 98.60 | 4.45 | 46.71 | 2.81 | -51.89 |
| 95889 | A | 95.20 | 5.71 | 53.86 | 12.09 | -41.34 |
| 95745 | A | 94.60 | 4.92 | 44.00 | 4.31 | -50.60 |
| 95853 | A | 93.75 | 11.14 | 48.29 | 3.37 | -45.46 |
| 95753 | A | 93.50 | 9.93 | 56.14 | 14.34 | -37.36 |
| 95744 | A | 91.40 | 18.61 | 47.50 | 8.44 | -43.90 |
| 95756 | A | 89.40 | 6.97 | 49.14 | 4.49 | -40.26 |
| 95961 | A | 88.00 | 12.07 | 45.14 | 4.29 | -42.86 |
| 95750 | A | 85.50 | 9.74 | 44.29 | 3.19 | -41.21 |
| 95729 | A | 83.60 | 4.18 | 48.29 | 3.81 | -35.31 |
| 95826 | A | 83.33 | 15.73 | 47.57 | 8.50 | -35.76 |
| 95759 | A | 83.00 | 6.36 | 43.00 | 3.12 | -40.00 |
| 95883 | A | 82.40 | 3.72 | 47.86 | 8.51 | -34.54 |
| 95726 | A | 81.40 | 2.42 | 49.86 | 4.85 | -31.54 |
| 95738 | A | 81.33 | 8.50 | 47.14 | 3.04 | -34.19 |
| 95915 | A | 80.40 | 9.00 | 45.14 | 3.36 | -35.26 |
| 95730 | A | 80.00 | 11.02 | 48.00 | 10.64 | -32.00 |
| 95737 | A | 79.33 | 9.41 | 44.43 | 3.70 | -34.90 |
| 95746 | A | 78.50 | 12.01 | 41.57 | 3.42 | -36.93 |
| 95942 | A | 78.50 | 14.61 | 46.71 | 3.53 | -31.79 |
| 95842 | A | 76.60 | 8.94 | 42.86 | 1.64 | -33.74 |
| 95929 | A | 76.00 | 7.09 | 39.00 | 5.32 | -37.00 |
| 95845 | A | 75.80 | 11.58 | 38.00 | 2.83 | -37.80 |
| 95782 | A | 74.60 | 6.89 | 44.71 | 4.62 | -29.89 |
| 95722 | A | 74.33 | 8.18 | 60.43 | 15.40 | -13.90 |
| 95950 | A | 71.40 | 5.54 | 38.86 | 4.16 | -32.54 |
| 95706 | A | 70.20 | 7.88 | 56.29 | 11.58 | -13.91 |
| 96105 | A | 70.17 | 4.02 | 42.57 | 1.99 | -27.60 |
| 95735 | A | 70.00 | 9.43 | 46.14 | 2.85 | -23.86 |
| 95963 | A | 69.00 | 4.16 | 41.14 | 3.94 | -27.86 |
| 95736 | A | 68.67 | 8.90 | 50.86 | 6.47 | -17.81 |
| 95903 | A | 63.00 | 7.70 | 39.14 | 3.31 | -23.86 |
| 95944 | A | 61.17 | 0.90 | 41.43 | 3.96 | -19.74 |
| 95799 | B | 53.50 | 1.89 | 39.00 | 3.59 | -14.50 |
| 96192 | C | 46.17 | 8.88 | 37.57 | 2.82 | -8.60 |
| 96143 | C | 45.33 | 9.36 | 38.14 | 2.03 | -7.19 |
| 95916 | C | 44.00 | 2.71 | 39.00 | 2.14 | -5.00 |
| 96145 | C | 43.50 | 2.50 | 38.57 | 2.26 | -4.93 |
| 96117 | C | 43.00 | 3.51 | 36.14 | 0.64 | -6.86 |
| 96102 | C | 42.67 | 5.31 | 37.43 | 1.84 | -5.24 |
| 96188 | C | 42.00 | 1.41 | 40.86 | 3.14 | -1.14 |
| 96194 | C | 41.50 | 0.96 | 39.33 | 3.45 | -2.17 |
| 96116 | C | 41.33 | 2.13 | 37.57 | 2.82 | -3.76 |
| 96129 | D | 41.00 | 5.86 | 40.00 | 2.39 | -1.00 |
| 96375 | C | 40.67 | 3.59 | 36.29 | 0.88 | -4.38 |
| 96217 | C | 40.50 | 2.75 | 35.86 | 1.46 | -4.64 |
| 96184 | D | 40.50 | 2.14 | 38.57 | 3.11 | -1.93 |
| 96196 | D | 40.33 | 5.12 | 36.86 | 0.99 | -3.48 |
| 96179 | D | 40.17 | 5.08 | 37.43 | 2.82 | -2.74 |
| 96161 | C | 40.00 | 4.97 | 36.71 | 2.49 | -3.29 |
| 96162 | C | 40.00 | 2.58 | 36.71 | 1.58 | -3.29 |
| 96128 | D | 39.83 | 2.91 | 42.29 | 7.07 | 2.45 |
| 96131 | D | 39.83 | 2.85 | 45.86 | 7.49 | 6.02 |
| 96245 | C | 39.50 | 3.64 | 33.43 | 2.32 | -6.07 |
| 96209 | C | 39.33 | 2.87 | 38.57 | 3.46 | -0.76 |
| 96185 | C | 39.00 | 2.65 | 34.14 | 1.46 | -4.86 |
| 96233 | C | 39.00 | 2.65 | 34.43 | 1.40 | -4.57 |
| 96132 | D | 38.83 | 2.34 | 36.29 | 0.45 | -2.55 |
| 96160 | D | 38.83 | 1.67 | 37.29 | 2.25 | -1.55 |
| 95796 | D | 38.50 | 2.93 | 37.57 | 4.27 | -0.93 |
| 95932 | D | 38.50 | 2.93 | 38.83 | 2.11 | 0.33 |
| 96173 | C | 38.33 | 2.43 | 36.86 | 1.88 | -1.48 |
| 96158 | D | 38.33 | 2.56 | 37.57 | 4.87 | -0.76 |
| 96120 | D | 38.17 | 2.27 | 36.29 | 1.16 | -1.88 |
| 96182 | D | 38.17 | 1.86 | 38.67 | 2.05 | 0.50 |
| 96180 | C | 38.00 | 1.63 | 36.29 | 0.45 | -1.71 |
| 95931 | D | 38.00 | 2.16 | 39.71 | 1.91 | 1.71 |
| 96216 | C | 37.83 | 1.46 | 36.43 | 1.40 | -1.40 |
| 96134 | C | 37.83 | 0.90 | 37.71 | 2.19 | -0.12 |
| 96101 | C | 37.83 | 1.07 | 39.43 | 4.24 | 1.60 |
| 95765 | D | 37.83 | 1.46 | 36.57 | 1.59 | -1.26 |
| 96164 | D | 37.83 | 1.57 | 38.43 | 3.25 | 0.60 |
| 96195 | C | 37.67 | 0.75 | 37.00 | 2.62 | -0.67 |
| 96155 | D | 37.67 | 0.94 | 38.14 | 2.53 | 0.48 |
| 96150 | D | 37.67 | 2.29 | 40.00 | 3.70 | 2.33 |
| 96178 | C | 37.17 | 0.37 | 37.43 | 2.61 | 0.26 |
| 96148 | D | 37.00 | 1.29 | 36.57 | 0.90 | -0.43 |

# 1.9 Supplementary Table S9. Comparison of LanFTc1_INDEL2 marker scores and time to flowering in response to vernalization observed during ‘2015 experiment.

| **Line** | **LanFTc1 INDEL2 marker** | **Days to flowering of non-vernalized plants '2015 (Mean)** | **Days to flowering of non-vernalized plants '2015 (Standard deviation)** | **Days to flowering of vernalized plants '2015 (Mean)** | **Days to flowering of vernalized plants '2015 (Standard deviation)** | **Change in days to flowering after vernalization '2015 (Mean)** |
| --- | --- | --- | --- | --- | --- | --- |
| 95896 | A | 101.50 | 3.91 | 68.00 | 0.89 | -33.50 |
| 95919 | A | 101.00 | 1.73 | 71.00 | 2.45 | -30.00 |
| 95702 | A | 98.50 | 4.09 | 70.80 | 3.19 | -27.70 |
| 96372 | A | 97.25 | 4.32 | 68.80 | 2.40 | -28.45 |
| 95948 | A | 96.80 | 5.46 | 61.20 | 0.98 | -35.60 |
| 95703 | A | 96.75 | 3.27 | 67.00 | 2.28 | -29.75 |
| 95711 | A | 96.25 | 3.90 | 63.20 | 2.79 | -33.05 |
| 95754 | A | 96.25 | 4.38 | 64.00 | 2.53 | -32.25 |
| 95947 | A | 94.80 | 0.98 | 67.80 | 1.60 | -27.00 |
| 95705 | A | 93.00 | 5.62 | 69.00 | 1.67 | -24.00 |
| 95852 | A | 92.50 | 4.97 | 66.20 | 4.21 | -26.30 |
| 95734 | A | 92.00 | 6.63 | 67.40 | 2.24 | -24.60 |
| 95704 | A | 91.40 | 4.59 | 73.80 | 1.47 | -17.60 |
| 96234 | A | 90.80 | 7.00 | 58.80 | 3.19 | -32.00 |
| 95883 | A | 90.75 | 2.49 | 68.40 | 4.50 | -22.35 |
| 95729 | A | 90.00 | 0.00 | 68.60 | 2.73 | -21.40 |
| 95716 | A | 90.00 | 4.00 | 71.40 | 1.62 | -18.60 |
| 95750 | A | 89.75 | 2.95 | 72.00 | 2.83 | -17.75 |
| 95889 | A | 88.75 | 2.05 | 72.60 | 1.96 | -16.15 |
| 95726 | A | 88.00 | 2.28 | 67.80 | 0.98 | -20.20 |
| 95753 | A | 88.00 | 3.69 | 70.60 | 3.14 | -17.40 |
| 95929 | A | 87.75 | 4.82 | 65.40 | 5.75 | -22.35 |
| 95915 | A | 87.60 | 2.33 | 70.80 | 4.07 | -16.80 |
| 95744 | A | 87.20 | 2.86 | 62.00 | 3.41 | -25.20 |
| 95853 | A | 87.00 | 3.00 | 66.60 | 4.08 | -20.40 |
| 95737 | A | 86.75 | 0.83 | 63.00 | 2.37 | -23.75 |
| 95745 | A | 85.60 | 3.14 | 70.60 | 2.42 | -15.00 |
| 95730 | A | 85.20 | 7.73 | 62.00 | 2.19 | -23.20 |
| 95735 | A | 85.20 | 2.71 | 72.60 | 4.84 | -12.60 |
| 95701 | A | 85.00 | 1.26 | 67.60 | 0.80 | -17.40 |
| 95706 | A | 84.60 | 2.94 | 65.00 | 1.90 | -19.60 |
| 95950 | A | 84.60 | 1.96 | 67.60 | 15.20 | -17.00 |
| 95759 | A | 84.50 | 0.87 | 61.60 | 1.50 | -22.90 |
| 95756 | A | 83.60 | 1.96 | 64.80 | 1.17 | -18.80 |
| 95782 | A | 83.50 | 2.06 | 66.40 | 1.50 | -17.10 |
| 95961 | A | 83.20 | 3.31 | 67.40 | 4.03 | -15.80 |
| 96105 | A | 83.20 | 0.98 | 69.20 | 5.27 | -14.00 |
| 95738 | A | 83.20 | 4.49 | 69.60 | 1.74 | -13.60 |
| 95845 | A | 82.75 | 6.65 | 59.60 | 3.01 | -23.15 |
| 95746 | A | 82.40 | 4.96 | 66.00 | 3.74 | -16.40 |
| 95942 | A | 82.40 | 5.68 | 68.20 | 4.07 | -14.20 |
| 95736 | A | 82.00 | 0.00 | 70.40 | 1.96 | -11.60 |
| 95963 | A | 81.40 | 1.20 | 59.20 | 2.14 | -22.20 |
| 95826 | A | 81.00 | 2.45 | 58.40 | 4.72 | -22.60 |
| 95722 | A | 80.80 | 1.47 | 62.20 | 2.99 | -18.60 |
| 95842 | A | 80.60 | 2.06 | 59.80 | 9.15 | -20.80 |
| 95944 | A | 80.20 | 1.60 | 57.20 | 2.99 | -23.00 |
| 95903 | A | 79.00 | 5.80 | 54.20 | 2.04 | -24.80 |
| 95799 | B | 68.20 | 5.60 | 53.40 | 1.02 | -14.80 |
| 96188 | C | 60.80 | 3.87 | 60.60 | 1.02 | -0.20 |
| 96129 | D | 60.80 | 1.83 | 56.40 | 2.80 | -4.40 |
| 96143 | C | 60.60 | 1.62 | 56.40 | 4.41 | -4.20 |
| 96192 | C | 59.60 | 2.58 | 55.80 | 1.17 | -3.80 |
| 96145 | C | 58.40 | 2.87 | 52.80 | 0.75 | -5.60 |
| 96217 | C | 58.40 | 5.54 | 58.40 | 7.42 | 0.00 |
| 96162 | C | 58.20 | 0.98 | 53.60 | 6.83 | -4.60 |
| 96194 | C | 58.20 | 6.68 | 55.00 | 1.41 | -3.20 |
| 96102 | C | 57.60 | 1.85 | 51.40 | 2.87 | -6.20 |
| 95916 | C | 57.60 | 4.59 | 52.00 | 1.26 | -5.60 |
| 96131 | D | 57.50 | 3.50 | 53.20 | 1.60 | -4.30 |
| 96160 | D | 57.40 | 2.24 | 52.00 | 1.26 | -5.40 |
| 96117 | C | 57.20 | 4.26 | 54.80 | 4.71 | -2.40 |
| 96161 | C | 57.20 | 3.71 | 49.80 | 0.98 | -7.40 |
| 96116 | C | 56.80 | 4.53 | 53.20 | 2.04 | -3.60 |
| 96184 | D | 56.60 | 5.12 | 56.40 | 2.42 | -0.20 |
| 96185 | C | 56.40 | 5.54 | 52.20 | 1.47 | -4.20 |
| 96196 | D | 56.40 | 3.88 | 54.60 | 3.38 | -1.80 |
| 95931 | D | 56.40 | 0.49 | 52.60 | 4.54 | -3.80 |
| 96209 | C | 56.25 | 3.90 | 51.40 | 3.38 | -4.85 |
| 96375 | C | 56.20 | 3.49 | 52.40 | 1.74 | -3.80 |
| 96132 | D | 56.20 | 4.92 | 51.20 | 5.46 | -5.00 |
| 96182 | D | 56.20 | 0.40 | 56.20 | 7.91 | 0.00 |
| 96245 | C | 56.00 | 4.20 | 52.00 | 1.10 | -4.00 |
| 96158 | D | 56.00 | 4.15 | 56.80 | 3.92 | 0.80 |
| 96128 | D | 55.50 | 1.50 | 53.40 | 1.96 | -2.10 |
| 96179 | D | 55.40 | 2.15 | 53.00 | 0.89 | -2.40 |
| 96173 | C | 55.20 | 1.17 | 56.40 | 3.14 | 1.20 |
| 96134 | C | 55.20 | 1.94 | 55.80 | 1.17 | 0.60 |
| 95796 | D | 55.20 | 0.98 | 48.40 | 0.80 | -6.80 |
| 95932 | D | 55.20 | 3.87 | 52.40 | 7.39 | -2.80 |
| 96101 | C | 54.80 | 1.94 | 55.40 | 3.01 | 0.60 |
| 96155 | D | 54.80 | 1.94 | 54.40 | 1.20 | -0.40 |
| 96233 | C | 54.50 | 2.60 | 50.40 | 0.49 | -4.10 |
| 96120 | D | 54.40 | 2.06 | 53.60 | 1.02 | -0.80 |
| 96195 | C | 54.20 | 0.40 | 50.40 | 3.01 | -3.80 |
| 96150 | D | 54.20 | 1.33 | 52.60 | 2.87 | -1.60 |
| 96216 | C | 54.00 | 2.19 | 51.00 | 2.45 | -3.00 |
| 96178 | C | 54.00 | 0.00 | 55.40 | 5.68 | 1.40 |
| 96180 | C | 53.60 | 1.96 | 52.20 | 4.49 | -1.40 |
| 96164 | D | 53.20 | 0.98 | 51.80 | 1.17 | -1.40 |
| 96148 | D | 52.60 | 1.20 | 52.40 | 3.61 | -0.20 |
| 95765 | D | 52.20 | 1.47 | 52.40 | 2.33 | 0.20 |

# Supplementary Figures


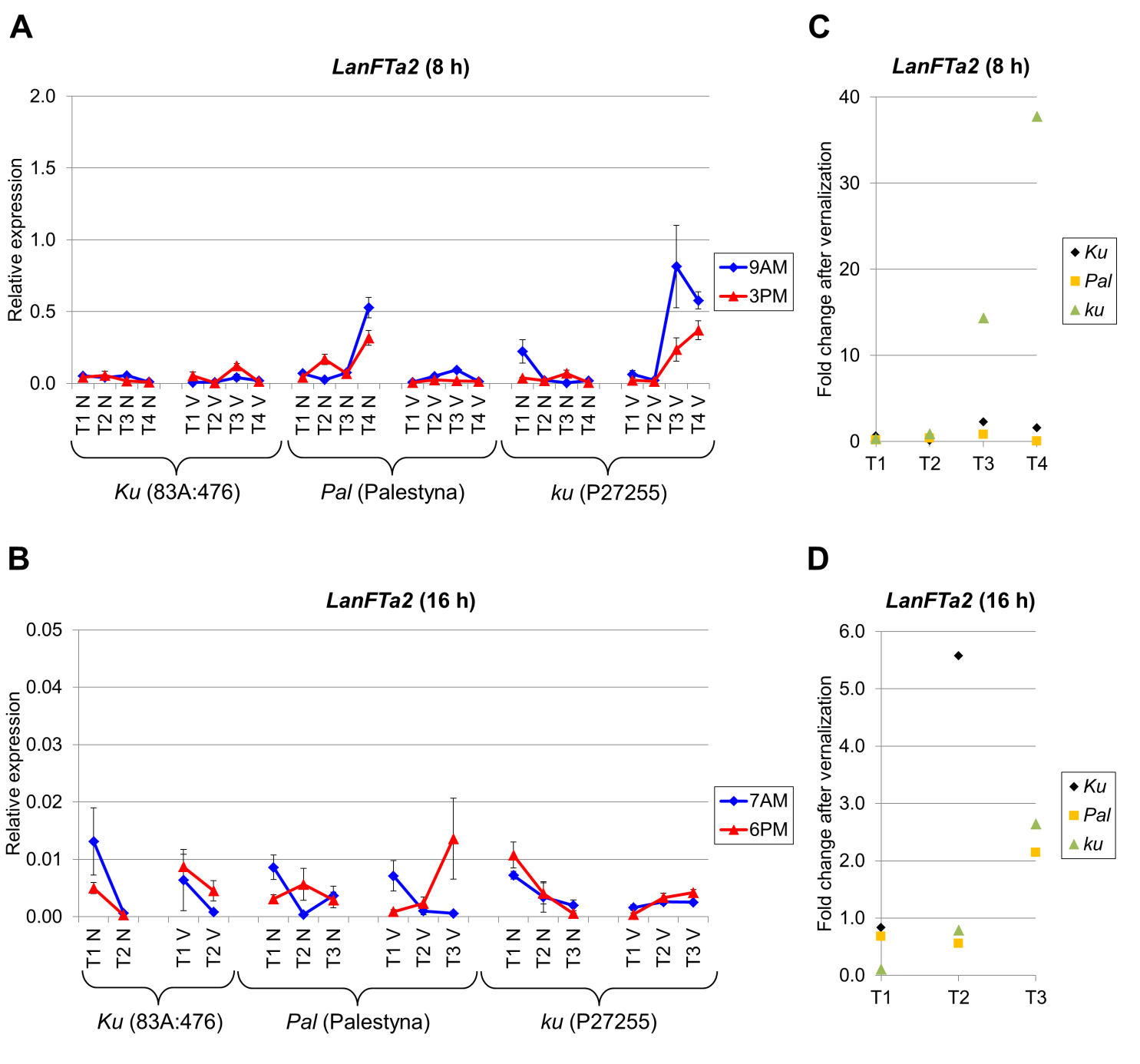


**Supplementary Figure 1.** Gene expression profile of *LanFTa2* gene in response to photoperiod and vernalization in three lines (83A:476, Palestyna and P27255) carrying different *LanFTc1* alleles (*Ku*, *Pal* and *ku*). (**A**) expression under 8-hour photoperiod, (**B**) expression under 16-hour photoperiod, (**C**) vernalization response under 8-hour photoperiod, (**D**) vernalization response under 16-hour photoperiod. T1-T4 stand for sampling terms (Supplementary Table S5), V for vernalized plants and N for non-vernalized plants. Time span of photoperiods: 8-hour from 4 AM to 8 PM, 16-hour from 4 AM to 8 PM. Two references were used for normalization (*LanDExH7* and *LanTUB6*) and one sample (*LanTUB6*) for inter-run calibration. Error bars indicate standard deviation of 3 biological replicates, each representing a mean of 3 technical replicates.


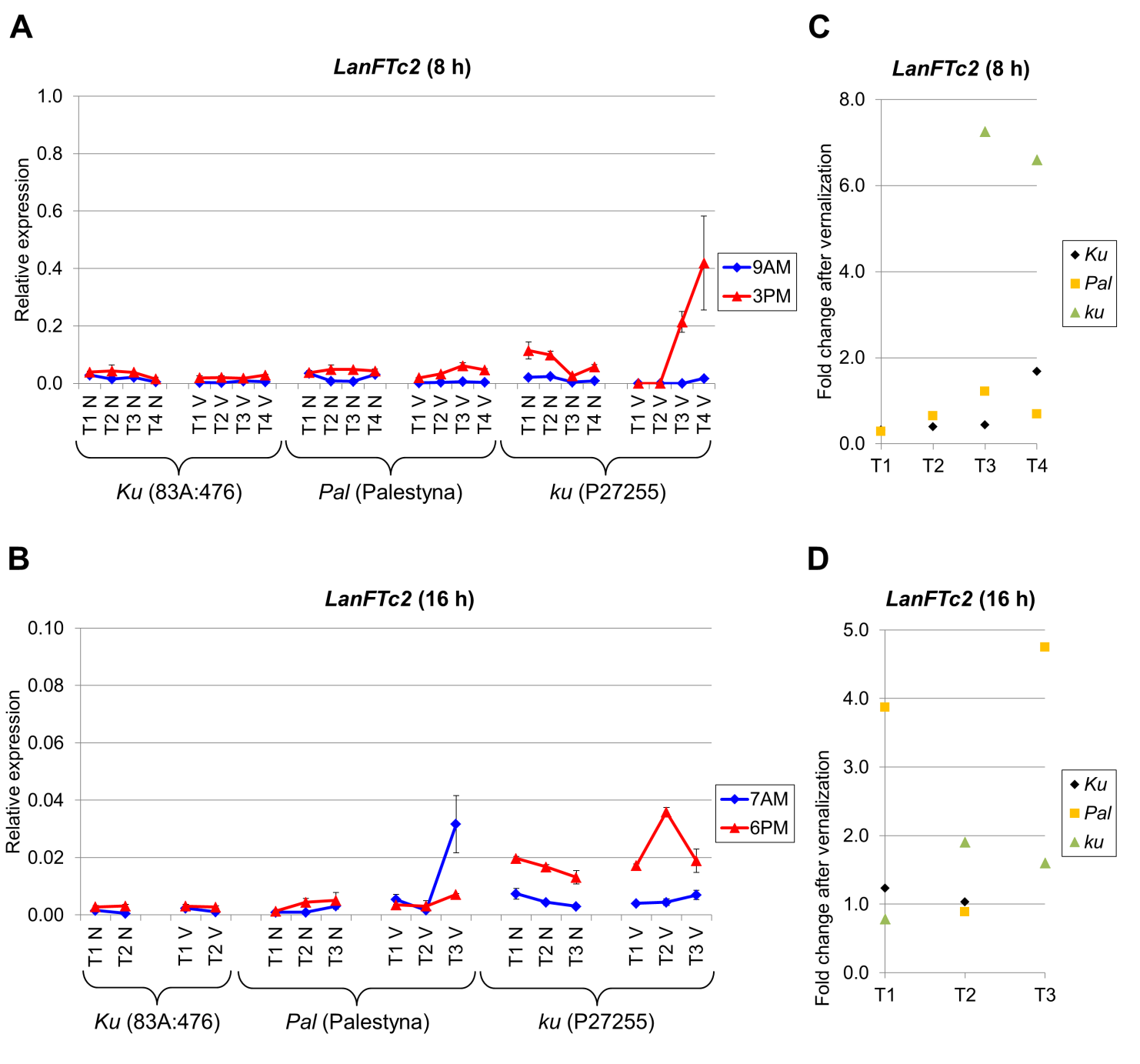


**Supplementary Figure 2.** Gene expression profile of *LanFTc2* gene in response to photoperiod and vernalization in three lines (83A:476, Palestyna and P27255) carrying different *LanFTc1* alleles (*Ku*, *Pal* and *ku*). (**A**) expression under 8-hour photoperiod, (**B**) expression under 16-hour photoperiod, (**C**) vernalization response under 8-hour photoperiod, (**D**) vernalization response under 16-hour photoperiod. T1-T4 stand for sampling terms (Supplementary Table S5), V for vernalized plants and N for non-vernalized plants. Time span of photoperiods: 8-hour from 4 AM to 8 PM, 16-hour from 4 AM to 8 PM. Two references were used for normalization (*LanDExH7* and *LanTUB6*) and one sample (*LanTUB6*) for inter-run calibration. Error bars indicate standard deviation of 3 biological replicates, each representing a mean of 3 technical replicates.


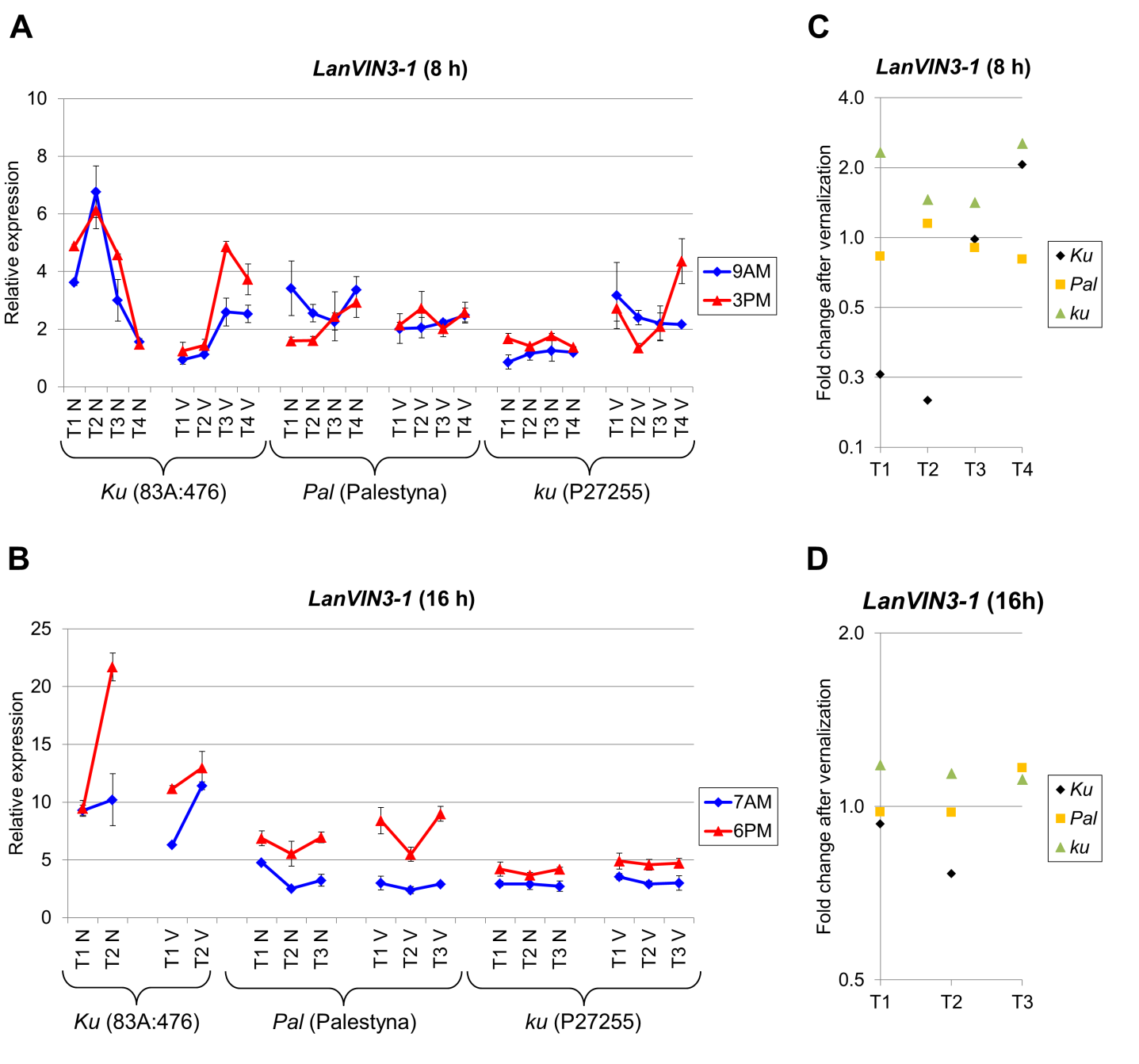


**Supplementary Figure 3.** Gene expression profile of *LanVIN3-1* gene in response to photoperiod and vernalization in three lines (83A:476, Palestyna and P27255) carrying different *LanFTc1* alleles (*Ku*, *Pal* and *ku*). (**A**) expression under 8-hour photoperiod, (**B**) expression under 16-hour photoperiod, (**C**) vernalization response under 8-hour photoperiod, (**D**) vernalization response under 16-hour photoperiod. T1-T4 stand for sampling terms (Supplementary Table S5), V for vernalized plants and N for non-vernalized plants. Time span of photoperiods: 8-hour from 4 AM to 8 PM, 16-hour from 4 AM to 8 PM. Two references were used for normalization (*LanDExH7* and *LanTUB6*) and one sample (*LanTUB6*) for inter-run calibration. Error bars indicate standard deviation of 3 biological replicates, each representing a mean of 3 technical replicates.


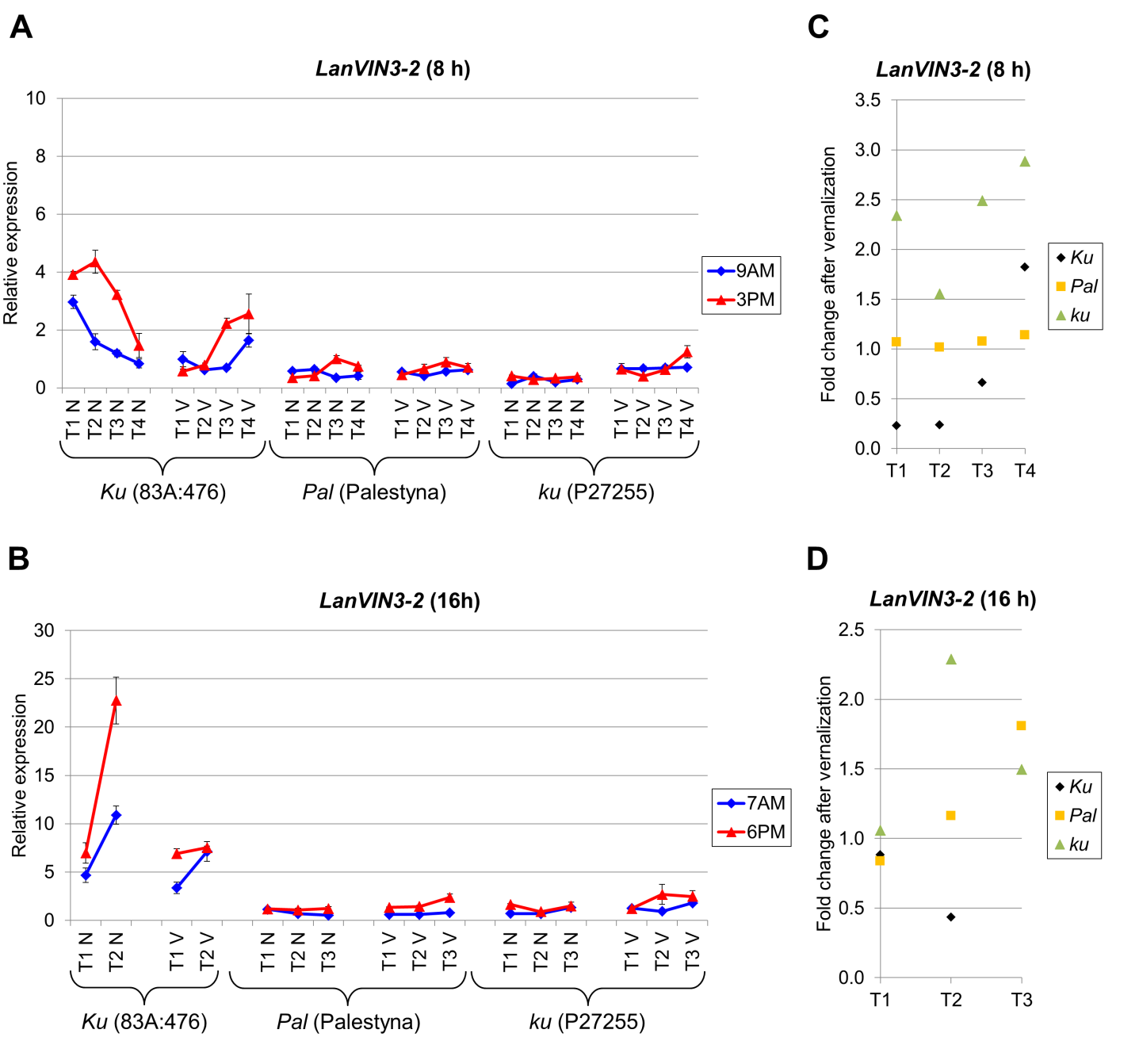


**Supplementary Figure 4.** Gene expression profile of *LanVIN3-2* gene in response to photoperiod and vernalization in three lines (83A:476, Palestyna and P27255) carrying different *LanFTc1* alleles (*Ku*, *Pal* and *ku*). (**A**) expression under 8-hour photoperiod, (**B**) expression under 16-hour photoperiod, (**C**) vernalization response under 8-hour photoperiod, (**D**) vernalization response under 16-hour photoperiod. T1-T4 stand for sampling terms (Supplementary Table S5), V for vernalized plants and N for non-vernalized plants. Time span of photoperiods: 8-hour from 4 AM to 8 PM, 16-hour from 4 AM to 8 PM. Two references were used for normalization (*LanDExH7* and *LanTUB6*) and one sample (*LanTUB6*) for inter-run calibration. Error bars indicate standard deviation of 3 biological replicates, each representing a mean of 3 technical replicates.


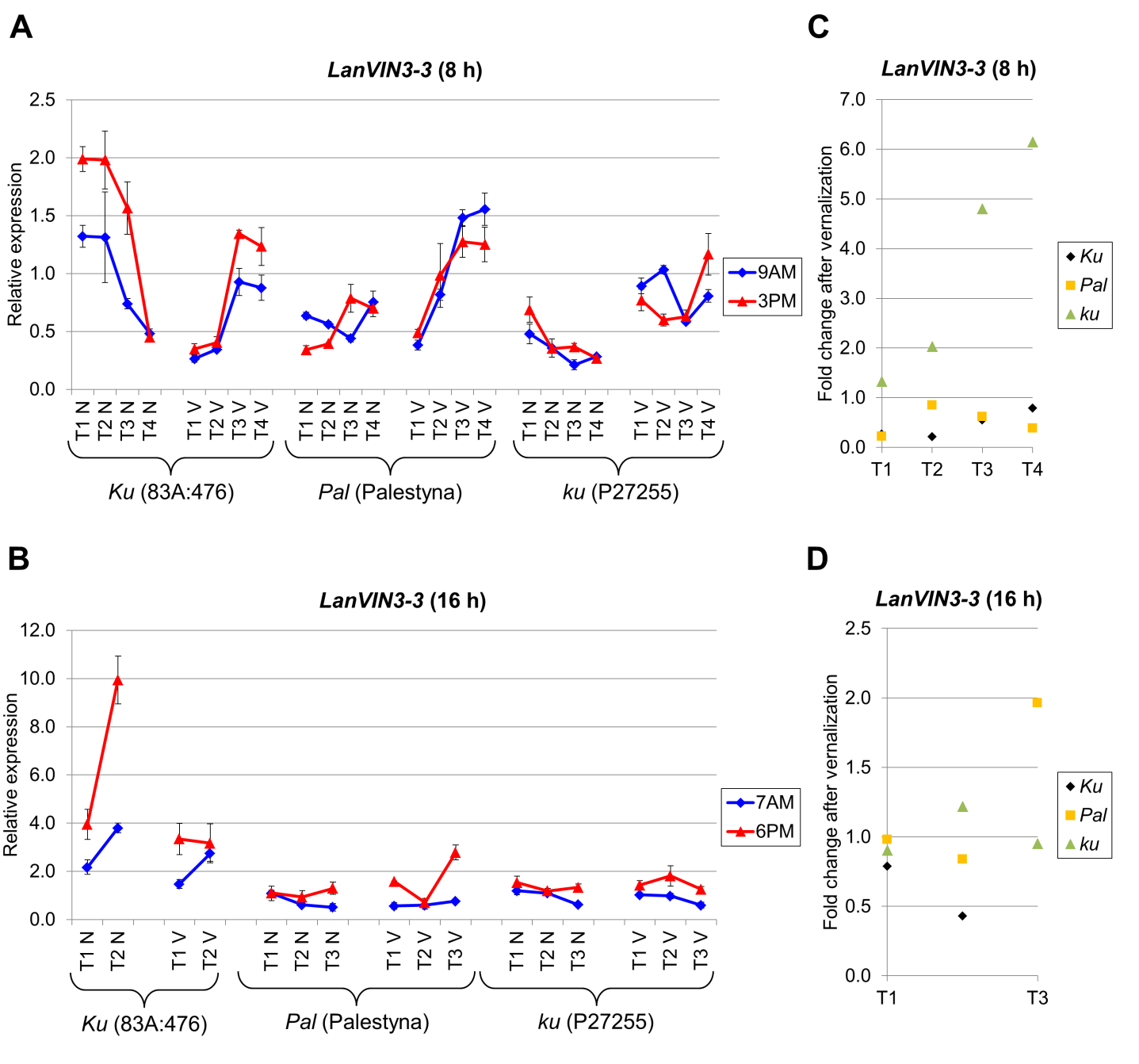


**Supplementary Figure 5.** Gene expression profile of *LanVIN3-3* gene in response to photoperiod and vernalization in three lines (83A:476, Palestyna and P27255) carrying different *LanFTc1* alleles (*Ku*, *Pal* and *ku*). (**A**) expression under 8-hour photoperiod, (**B**) expression under 16-hour photoperiod, (**C**) vernalization response under 8-hour photoperiod, (**D**) vernalization response under 16-hour photoperiod. T1-T4 stand for sampling terms (Supplementary Table S5), V for vernalized plants and N for non-vernalized plants. Time span of photoperiods: 8-hour from 4 AM to 8 PM, 16-hour from 4 AM to 8 PM. Two references were used for normalization (*LanDExH7* and *LanTUB6*) and one sample (*LanTUB6*) for inter-run calibration. Error bars indicate standard deviation of 3 biological replicates, each representing a mean of 3 technical replicates.


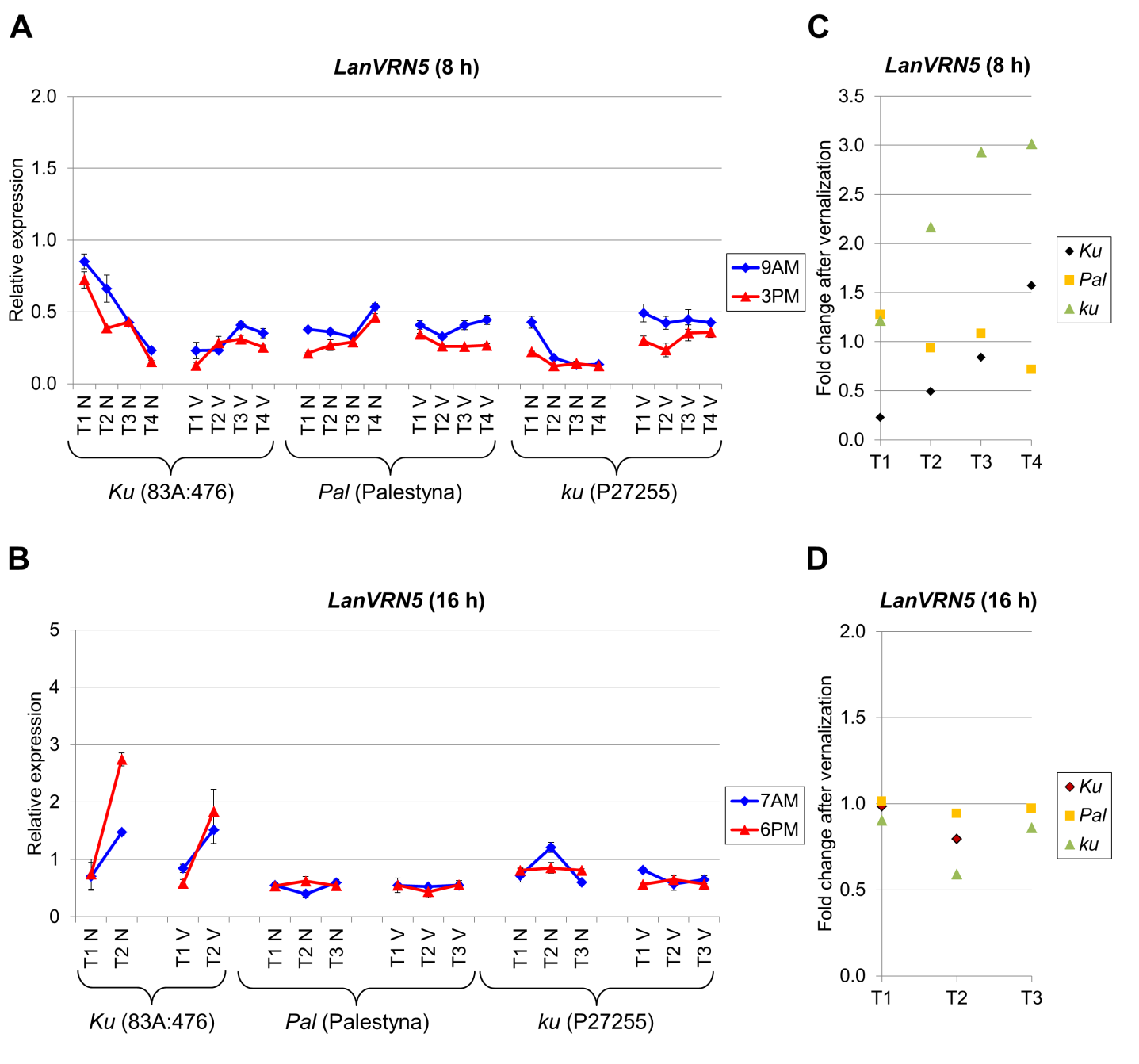


**Supplementary Figure 6.** Gene expression profile of *LanVRN5* gene in response to photoperiod and vernalization in three lines (83A:476, Palestyna and P27255) carrying different *LanFTc1* alleles (*Ku*, *Pal* and *ku*). (**A**) expression under 8-hour photoperiod, (**B**) expression under 16-hour photoperiod, (**C**) vernalization response under 8-hour photoperiod, (**D**) vernalization response under 16-hour photoperiod. T1-T4 stand for sampling terms (Supplementary Table S5), V for vernalized plants and N for non-vernalized plants. Time span of photoperiods: 8-hour from 4 AM to 8 PM, 16-hour from 4 AM to 8 PM. Two references were used for normalization (*LanDExH7* and *LanTUB6*) and one sample (*LanTUB6*) for inter-run calibration. Error bars indicate standard deviation of 3 biological replicates, each representing a mean of 3 technical replicates.
