# Supplementary figures and images for: Photoperiod and Vernalization Control of Flowering-Related Genes: A Case Study of the Narrow-Leafed Lupin (Lupinus angustifolius L.)
Source: Front Plant Sci. 2020 Oct 26;11:572135. doi: 10.3389/fpls.2020.572135 (PMC7663182; doi:10.3389/fpls.2020.572135)

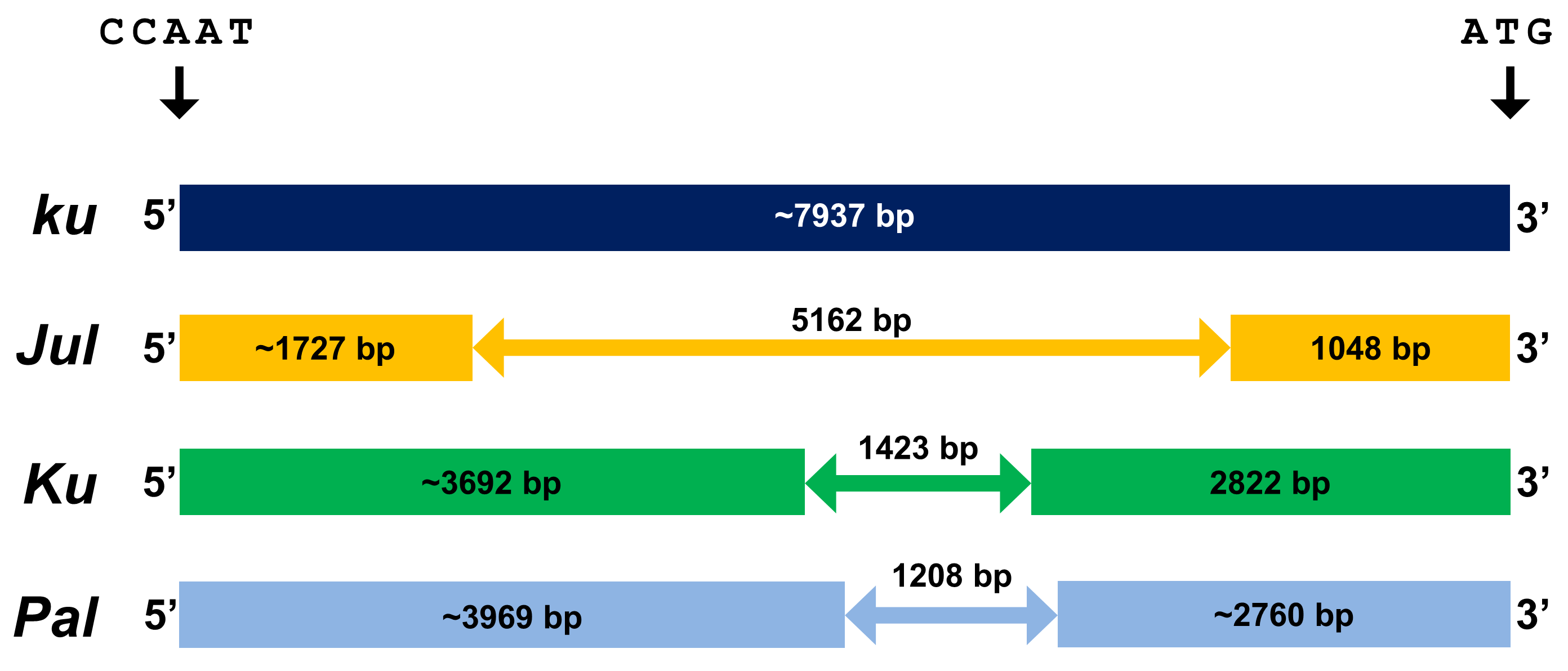

Supplement: Supplementary Figure 1 — Gene expression profile of the LanFTa2 gene in response to photoperiod and vernalization in three lines (83A:476, Palestyna, and P27255) carrying different LanFTc1 alleles (Ku, Pal, and ku). [file Figure_1.TIF]

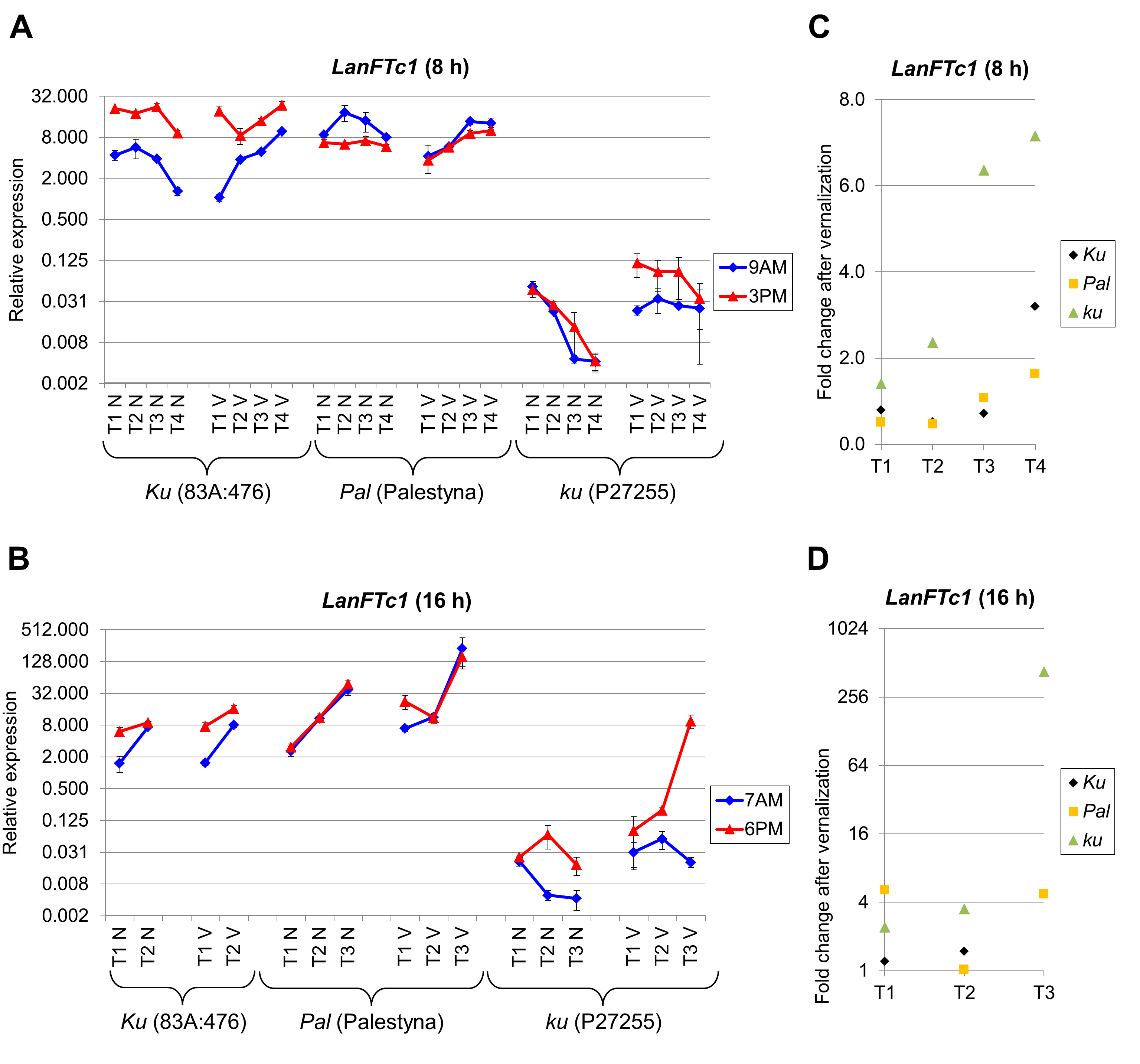

Supplement: Supplementary Figure 2 — Gene expression profile of the LanFTc2 gene in response to photoperiod and vernalization in three lines (83A:476, Palestyna, and P27255) carrying different LanFTc1 alleles (Ku, Pal, and ku). [file Figure_2.TIF]

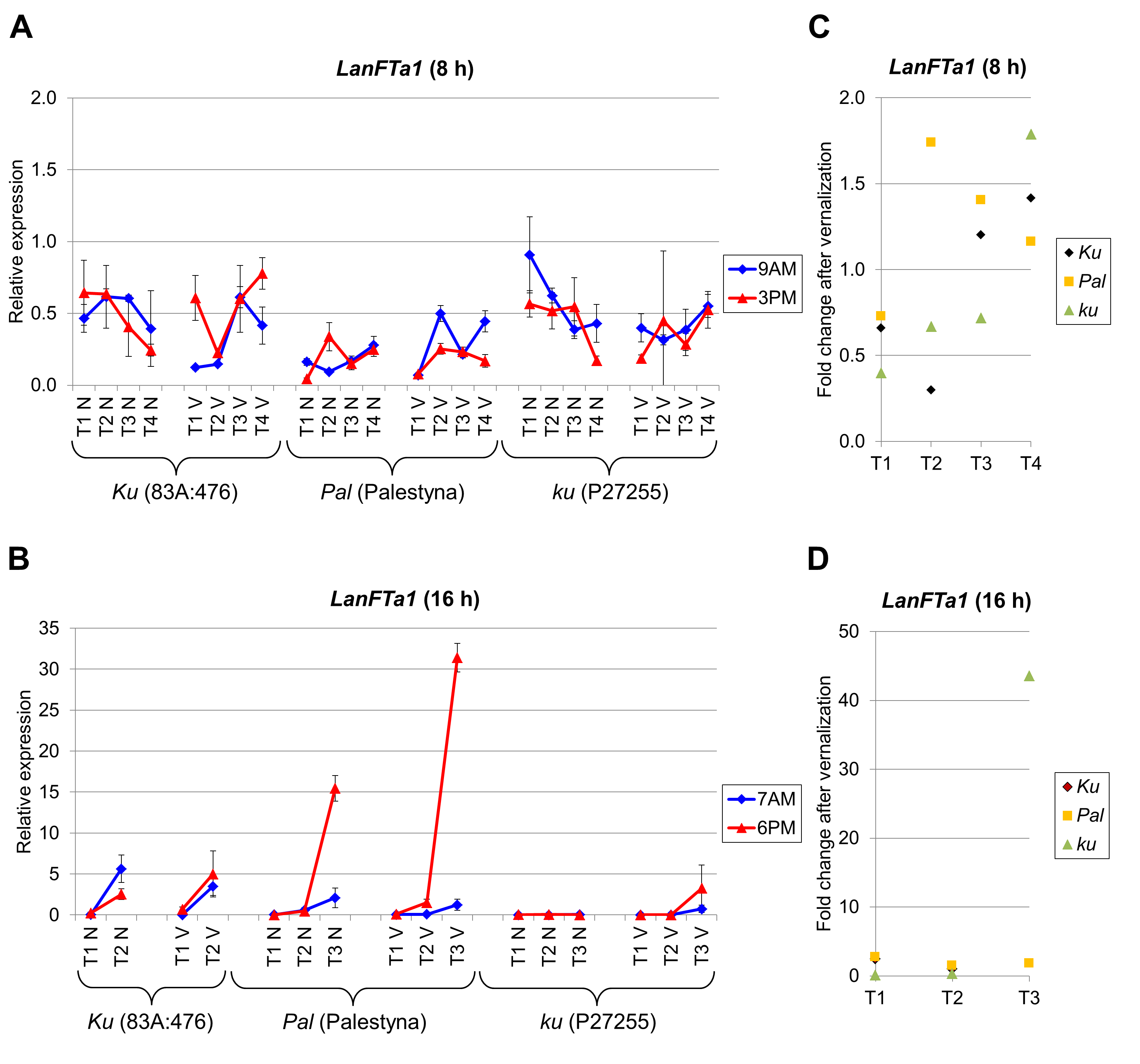

Supplement: Supplementary Figure 3 — Gene expression profile of the LanVIN3-1 gene in response to photoperiod and vernalization in three lines (83A:476, Palestyna, and P27255) carrying different LanFTc1 alleles (Ku, Pal, and ku). [file Figure_3.TIF]

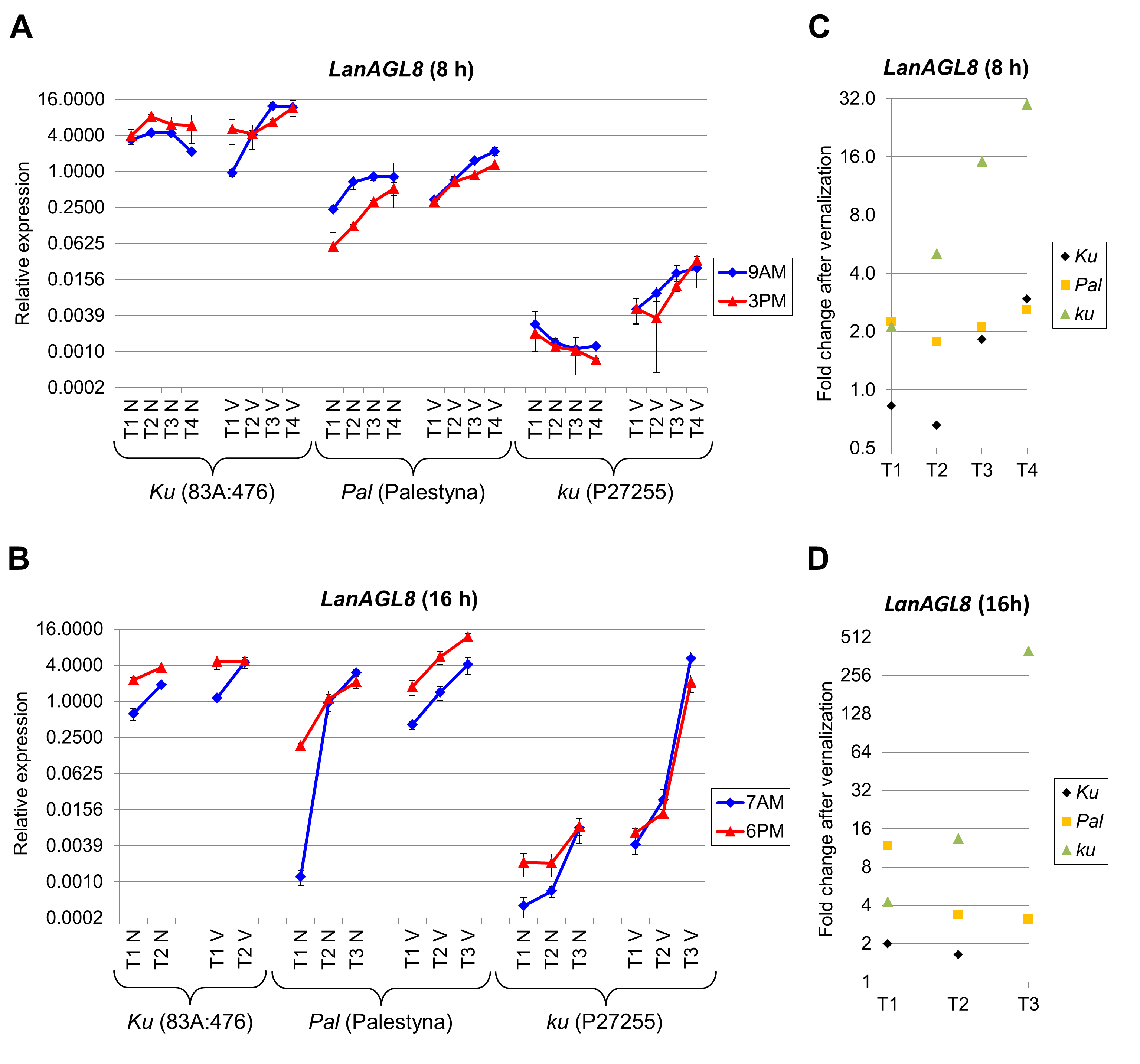

Supplement: Supplementary Figure 4 — Gene expression profile of the LanVIN3-2 gene in response to photoperiod and vernalization in three lines (83A:476, Palestyna, and P27255) carrying different LanFTc1 alleles (Ku, Pal, and ku). [file Figure_4.TIF]

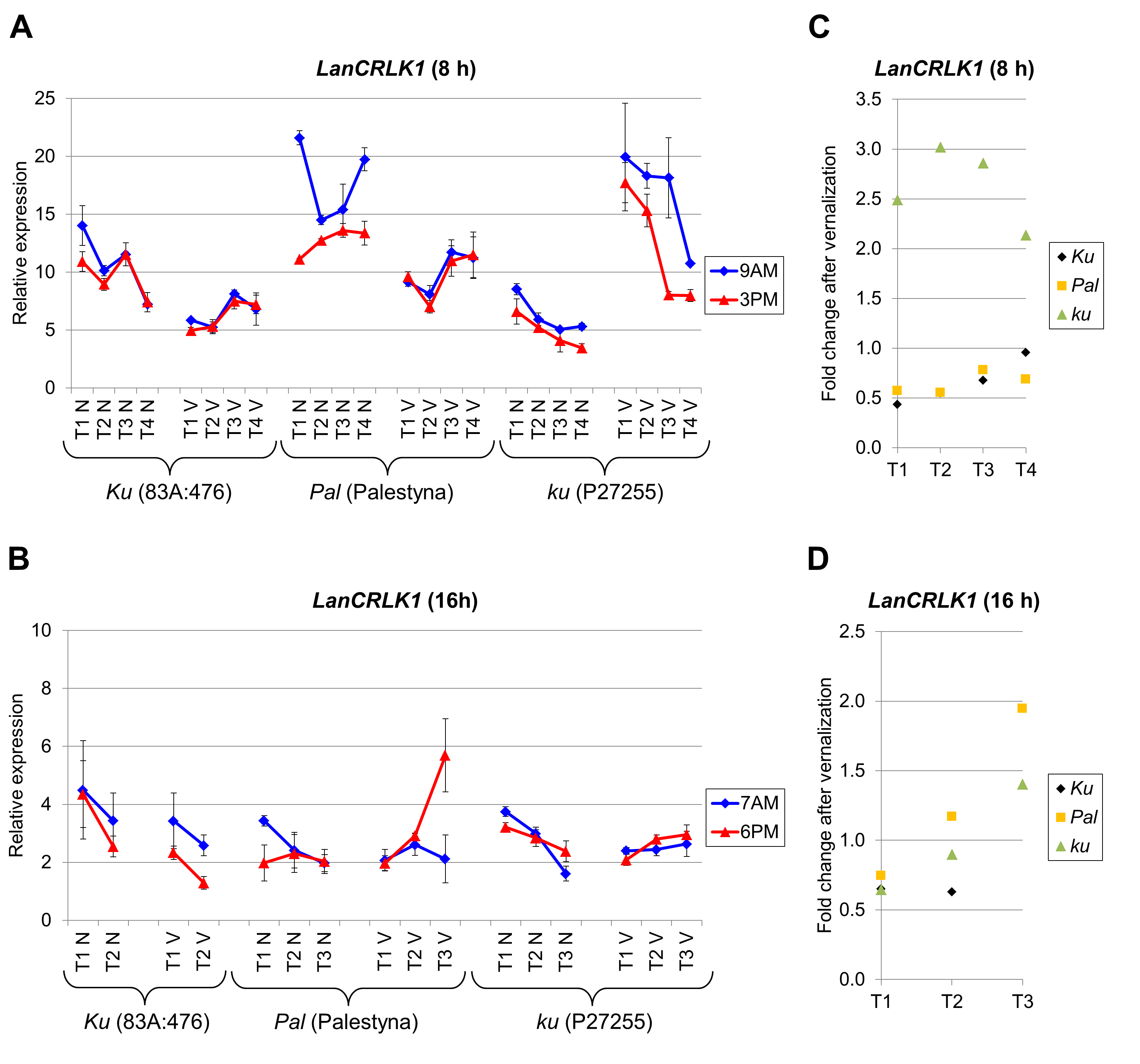

Supplement: Supplementary Figure 5 — Gene expression profile of the LanVIN3-3 gene in response to photoperiod and vernalization in three lines (83A:476, Palestyna, and P27255) carrying different LanFTc1 alleles (Ku, Pal, and ku). [file Figure_5.TIF]

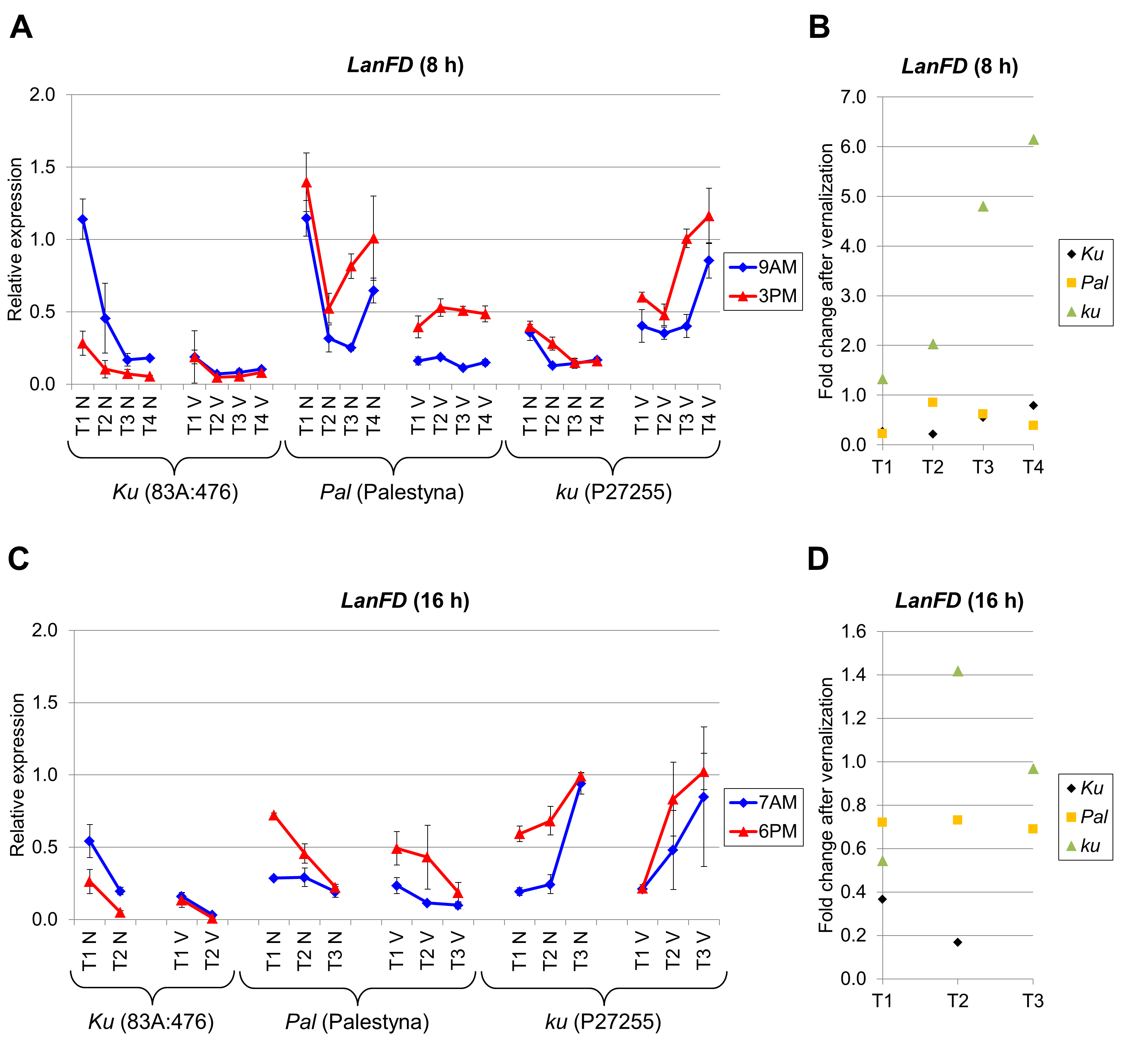

Supplement: Supplementary Figure 6 — Gene expression profile of LanVRN5 genes in response to photoperiod and vernalization in three lines (83A:476, Palestyna, and P27255) carrying different LanFTc1 alleles (Ku, Pal, and ku). [file Figure_6.TIF]

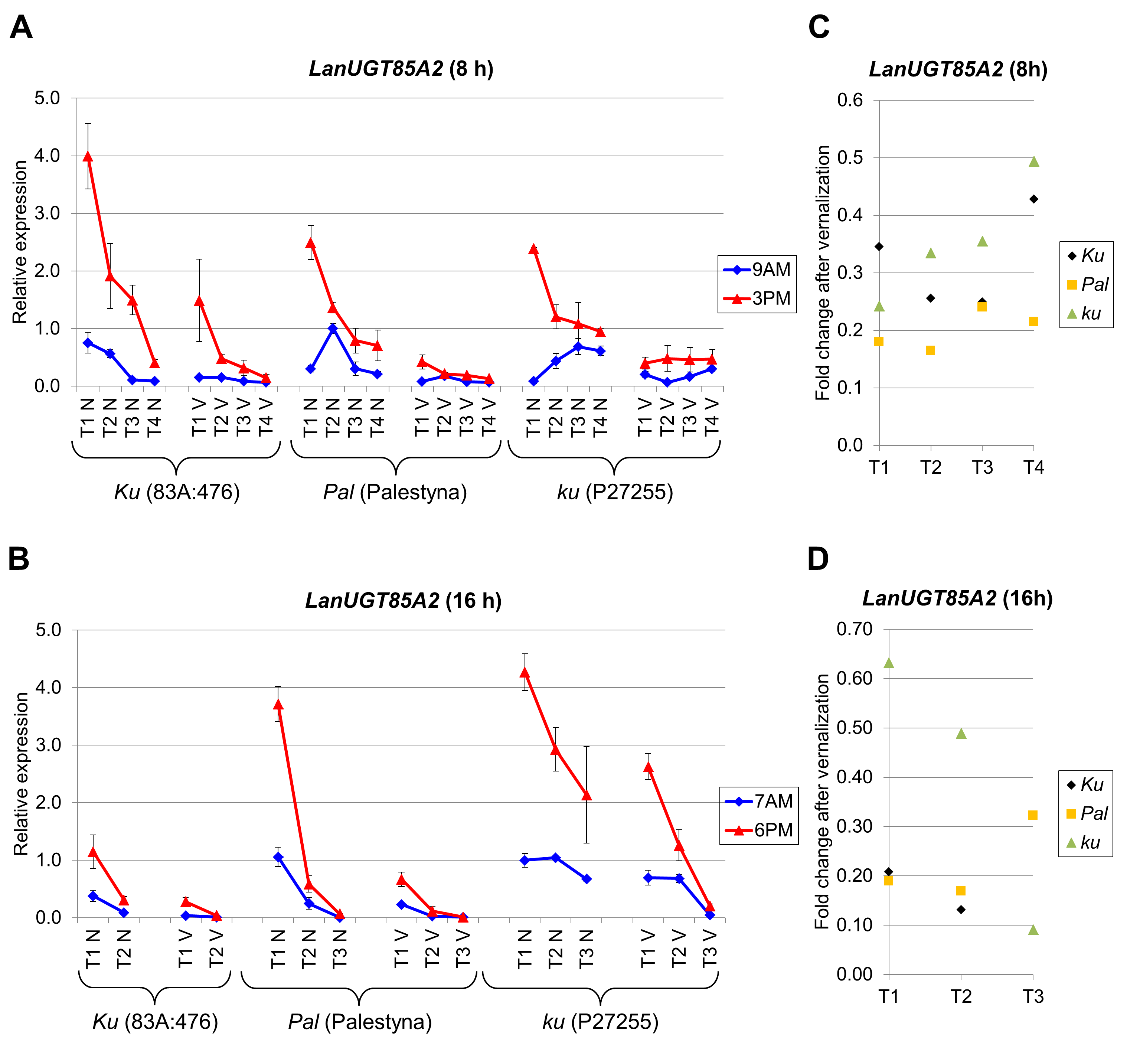

Supplement: Supplementary file 9 [file Figure_7.TIF]
